# Discovery of Q-Markers in traditional Chinese medicine based on machine learning: Application and verification in Xiaoyao Heji

**DOI:** 10.1016/j.chmed.2026.08.020

**Published:** 2026-09-02

**Authors:** Guotao Chen, Jing Hu, Aijing Li, Yu Zeng, Qingmei Mo, Kairui Tang, Jiaxu Chen, Xinsheng Yao, Yongxia Guan, Guimin Zhang, Liangliang He, Zhihong Yao

**Affiliations:** a State Key Laboratory of Bioactive Molecules and Druggability Assessment/International Cooperative Laboratory of Traditional Chinese Medicine Modernization and Innovative Drug Development of Ministry of Education (MOE) of China/Guangdong Province Key Laboratory of Pharmacodynamic Constituents of TCM and New Drugs Research, College of Pharmacy, Jinan University, Guangzhou 510632, China; b Guangzhou Key Laboratory of Formula-Pattern of Traditional Chinese Medicine, School of Traditional Chinese Medicine, Jinan University, Guangzhou 510632, China; c State Key Laboratory of Integration and Innovation of Classic Formula and Modern Chinese Medicine, Lunan Pharmaceutical Group Co., Ltd., Linyi 276006, China

**Keywords:** machine learning, multi-dimensional features, Q-Marker, TCM prescription, Xiaoyao Heji

## Abstract

**Objective:**

Identification of quality marker (Q-Marker) is central to the modernization and quality control of traditional Chinese medicine (TCM). Existing methods, though diverse, lack a unified and scalable strategy to address the chemical and pharmacological complexity of TCM prescriptions. This study established a machine learning-driven framework to integrate multi-source data and systematically prioritize Q-Marker, using Xiaoyao Heji (XYHJ), a modern antidepressant formulation derived from Xiaoyao San, as a case study.

**Methods:**

Firstly, a dataset of 302 compounds was constructed by summarizing 14 reported Q-Marker discovery strategies and annotated with five core dimensions-compatibility, specificity, measurability, traceability, and effectiveness. Subsequently, five ranking models (RankNet, Random Forest, XGBoost, Linear Regression and K-Nearest Neighbors) were trained with 8-fold GroupKFold cross-validation and evaluated by normalized discounted cumulative gain (NDCG), mean average precision (MAP), and Recall. Thirdly, the optimal model was applied to XYHJ, followed by systematic characterization of chemical profiles, quantitative analysis, drug metabolism and pharmacokinetics (DMPK), quality transitivity, and bioactivity. Finally, Q-Markers were further validated through radar chart modeling, Monte Carlo simulations, and chemometric analyses.

**Results:**

Through a comparison of the machine learning models, XGBoost outperformed other models, exhibiting high and stable performance with NDCG, MAP, and Recall values ranging from 0.84 to 0.95. For the first time, the XYHJ prescription was comprehensively evaluated across its chemical profile, DMPK properties, quantitative content, manufacturing traceability, and biological efficacy. Using the optimal XGBoost ranking model, 15 components were screened and ranked, successfully identifying paeoniflorin, albiflorin, and glycyrrhizic acid as critical Q-Marker. The reliability of the ranking approach was further validated by integrating a previously published radar chart model with Monte Carlo simulations with over 10 000 sampling iterations. Furthermore, fingerprint similarity and chemometric analysis demonstrated the effectiveness of these Q-Markers in distinguishing XYHJ produced through different manufacturing processes.

**Conclusion:**

This study developed a machine learning-driven ranking framework that unifies multiple Q-Marker discovery strategies. Using XYHJ as a case study, this approach not only enhances its quality control system but also provides a valuable reference for the standardization and modernization of other TCM prescriptions.

## 1 Introduction

Traditional Chinese medicine (TCM) prescriptions have gained global attention for their holistic therapeutic potential, particularly in managing complex multi-system disorders (Ji et al., 2022). However, the absence of robust quality control systems aligned with modern pharmaceutical standards continues to impede their international acceptance (Xie & Leung, 2009). Against this backdrop, the concept of quality marker (Q-Marker), defined as chemically traceable constituents intrinsically linked to therapeutic efficacy and production consistency, has been introduced as a novel approach to reinforce TCM standardization (Liu et al., 2016, Wang et al., 2023). According to the team standard issued by the China Association of Chinese Medicine (CACM), which is named “the technical guide for research on quality marker of traditional Chinese medicine” (T/CACM 1638–2025), the features of Q-Marker were defined as compatibility environment, specificity, measurability, traceability, and effectiveness. As a result, Q-Markers serve as critical indicators for evaluating the safety, efficacy, and batch-to-batch reproducibility of TCM preparations across diverse dosage forms, including decoctions, extracts, and finished products (Hu et al., 2024, Wang et al., 2025).

Nowadays, various methodologies have been employed to screen Q-Marker, such as quantitative ternary network, “radar chart” mode, network pharmacology, entropy weight method, “spider-web” mode, and so on (He et al., 2021, Hu et al., 2024, Huang et al., 2021, Wang et al., 2023, Zhang et al., 2020). These approaches offer multiple perspectives and tools for Q-Marker identification, enriching the analytical strategies available for evaluating complex herbal systems. However, the diversity of these methodologies has also brought certain challenges. Firstly, each method is grounded in distinct theoretical frameworks and data models, often leading to inconsistent screening results. Secondly, the absence of unified evaluation criteria hinders cross-study comparability. For instance, components identified via the quantitative ternary network may differ significantly from those highlighted by the radar chart approach, making it difficult to determine which components are truly Q-Marker. Therefore, there is an urgent need to establish a unified analytical framework that can ensure methodological consistency and provide more robust evidence for the accurate identification of Q-Marker.

Machine learning is an artificial intelligence technique that utilizes algorithms to automatically learn patterns from data and make predictions or decisions (Ray, 2019). It has been widely applied across various fields, including medicine (Hamet & Tremblay, 2017), finance (Ahmed, Alshater, Ammari, & Hammami, 2022), and image recognition (Tian, 2020). In the field of TCM research, machine learning offers the capability to efficiently process complex, high-dimensional, and large-scale datasets, uncovering hidden nonlinear relationships among variables and exhibiting strong abilities in pattern recognition and feature extraction (Pan et al., 2024). For example, machine learning has been used to discover bioactive compounds from Chinese herbal medicines, elucidate their pharmacological mechanisms, and evaluate the therapeutic efficacy of herbal formulations (Khanal et al., 2025, Zhang et al., 2025). Particularly in Q-Marker screening tasks that involve multi-source data integration and multi-factor comprehensive evaluation, the incorporation of techniques such as supervised learning, ensemble learning, and learning to rank enables the organic integration of features, facilitates comprehensive evaluation and ranking of candidate Q-Marker, and enhances model consistency, reproducibility, and generalizability.

Xiaoyao San (XYS), a classical TCM prescription with well-documented clinical efficacy in alleviating depression and anxiety, was first recorded in the Song Dynasty’s “*Formulary of the Bureau of Taiping People’s Welfare Pharmacy*” (Chen et al., 2022). This formula consists of *Bupleuri Radix* (Chaihu in Chinese), *Paeoniae Radix Alba* (Baishao in Chinese), *Atractylodis Macrocephalae Rhizoma* (Baizhu in Chinese), *Glycyrrhizae Radix* et *Rhizoma* (Ganzao in Chinese), *Zingiberis Rhizoma Recens* (Shengjiang in Chinese), *Angelicae Sinensis Radix* (Danggui in Chinese), *Poria* (Fuling in Chinese) and *Menthae Haplocalycis Herba* (Bohe in Chinese), which collectively exert effects to relieve liver depression and regulate *qi*, producing an overall regulatory effect (He et al., 2024, Wu et al., 2022). Over the past millennium, XYS has been widely used in clinical practice, especially in the fields of gynecology and mental health. In recent decades, its antidepressant properties have been increasingly acknowledged, with evidence from randomized, double-blind clinical trials leading to its inclusion in integrated treatment guidelines combining traditional Chinese and Western medicine (Guo et al., 2020, Zhao, 2020). Nevertheless, despite its notable therapeutic outcomes, the current quality standard for XYS-related preparations relies solely on paeoniflorin as a quantitative indicator (Commission, 2020), which is far from guaranteeing efficacy and safety. Xiaoyao Heji (XYHJ), derived from traditional XYS, is a modern TCM preparation designed to maintain the rapid absorption and therapeutic efficiency of traditional decoctions while offering enhanced convenience (Luo et al., 2012). However, firstly, as a novel formulation derived from XYS, it currently lacks a well-defined chemical basis study and standardized quality control method, which highly hinders its clinical application and market competitiveness. Secondly, variability in component profiles across different manufacturing processes compromises therapeutic consistency, underscoring the need for rigorous quality consistency assessments in TCM prescription (Li et al., 2010, Liang et al., 2019). Conducting quality consistency assessments on XYHJ and XYS prescription, particularly for clinically effective, commercially available prescription, is therefore essential. Recent studies emphasize that a comprehensive quality assessment of TCM preparations must integrate *in vitro* compositional analysis with *in vivo* metabolic profiling to ensure therapeutic reliability. Thirdly, the key to establishing a robust quality control method for XYHJ lies in the identification of Q-Marker, but the research on Q-Marker for XYS or XYHJ remains limited.

In response to these challenges, a machine learning-driven method was developed to unify diverse Q-Marker identification strategies, enabling the automated ranking and prioritization of Q-Marker in TCM prescriptions. Firstly, this study systematically reviewed 14 distinct Q-Marker discovery methodologies reported in the literature and constructed a dataset comprising 302 compounds, each annotated with multidimensional features relevant to Q-Marker characteristics. Based on this dataset, three machine learning-based ranking models—RankNet, Random Forest, and XGBoost—were developed and evaluated through cross-validation to determine the optimal performing model. XYHJ, a representative modern Chinese medicinal formulation, was selected as a case study. The core feature dimensions of its constituent compounds were systematically characterized for the first time, and the optimized model was applied to identify potential Q-Marker. Finally, the reliability and robustness of the predicted Q-Marker were assessed by integrating a radar chart model and Monte Carlo simulations, as well as chemometric analysis. This study aims to establish a unified machine learning framework for Q-Marker identification, offering a novel perspective for standardizing Q-Marker screening and quality control in TCM (Fig. 1).

**Fig. 1:**
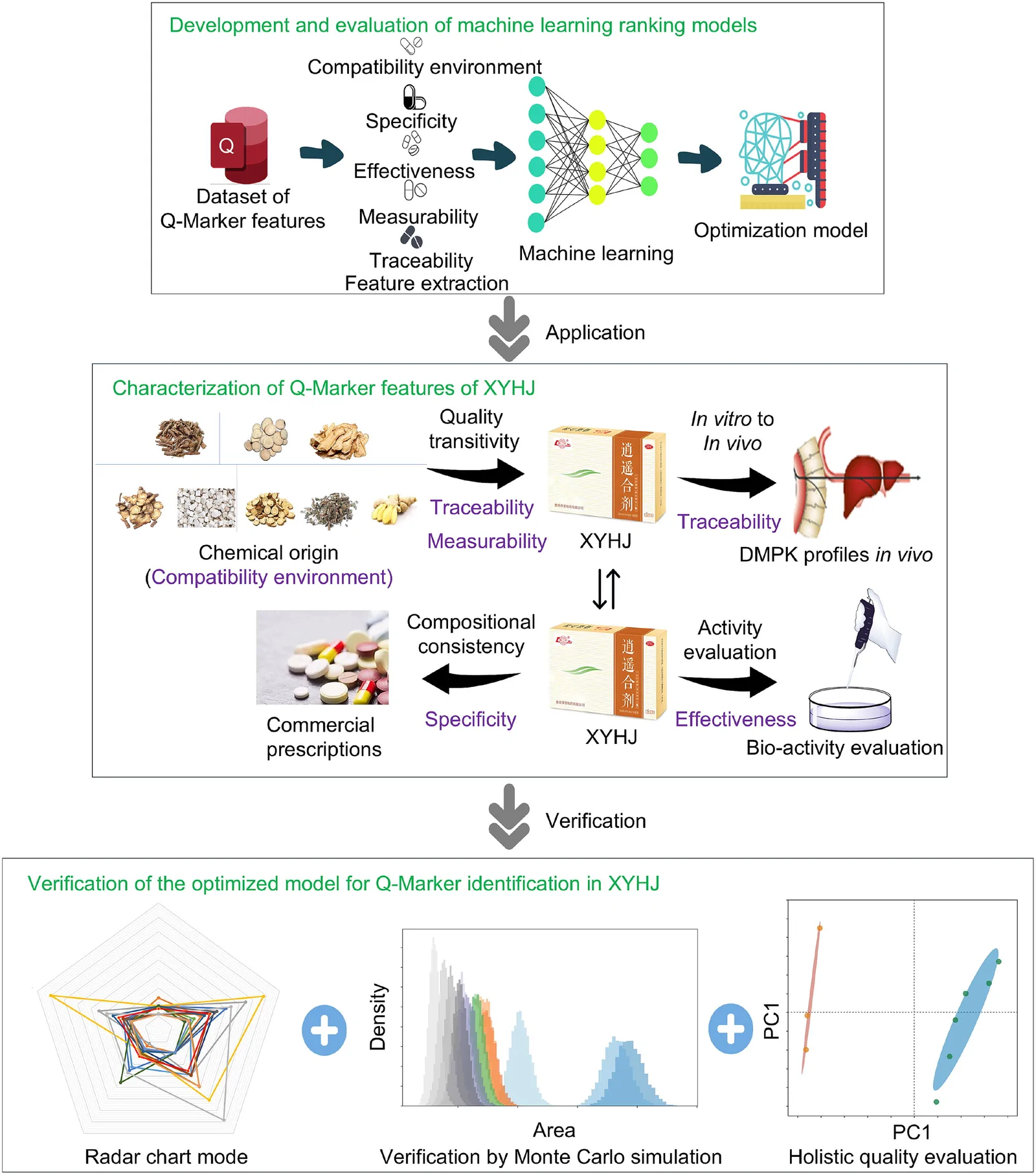
Graphic abstract of machine learning-driven identification and verification of Q-Markers in TCM prescription. DMPK: drug metabolism and pharmacokinetics.

## 2 Materials and methods

### 2.1 Materials and reagents

Reference standards, including paeoniflorin (Lot: CHB201205), isoliquiritin apioside (Lot: CHB161228), ferulic acid (Lot: CHB160913), chlorogenic acid (Lot: CHB90121), isoliquiritin (Lot: CHB201205), saikosaponin A (Lot: CHB180326), pentagalloylglucose (Lot: CHB190125), liquiritigenin (Lot: CHB180609), neoliquiritin (Lot: CHB201125), atractylenolide I (Lot: CHB201119), atractylenolide II (Lot: CHB170227), atractylenolide III (Lot: CHB201121), 6-gingerol (Lot: CHB201157), violanthin (Lot: CHB201141), gallic acid (Lot: CHB180114), *L*-tryptophan (Lot: CHB210115) were purchased from Chengdu Chroma-Biotechnology Co., Ltd. (Chengdu, China) and isoliquiritigenin (Lot: PS02340025), formononetin (Lot: PS02150020), benzoylpaeoniflorin (Lot: PS04690020), ononin (Lot: PS04410020), senkyunolide H (Lot: PS22560010), senkyunolide I (Lot: PS21690010), albiflorin (Lot: PS11680020), liquiritin (Lot: PS012028), ligustilide (Lot: PS000288), schaftoside (Lot: PS012445), glycyrrhizic acid (Lot: PS010448), glycyrrhetinic acid (Lot: PS010225), liquiritin apioside (Lot: PS011457), lactiflorin (Lot: PS307811) were obtained from Chengdu Push Bio-Technology Co., Ltd. (Chengdu, China). The purity of all reference standards exceeded 98% as confirmed by HPLC analysis. XYHJ was obtained from Lunan Pharmaceutical Group Co., Ltd. (Linyi, China), Xiaoyao Wan (XYW, concentrated pills) and Xiaoyao Keli (XYKL) were over-the-counter drugs collected from multiple pharmacies in China. LC/MS grade acetonitrile, methanol, and formic acid were purchased from Fisher Scientific (Fair Lawn, New Jersey, USA). Other chemicals and reagents used were of analytical grade. Heparin sodium was purchased from Shenzhen Huashi Science and Technology Co., Ltd. (Shenzhen, China), sodium chloride injection was obtained from Anhui Shuanghe Pharmaceutical Co., Ltd. (Hefei, China).

### 2.2 Development and evaluation of machine learning ranking models

The Q-Marker related features dataset construction: By summarizing 14 distinct Q-Marker discovery methods from the literatures, a dataset comprising 302 compounds was constructed, each annotated with multi-dimensional features relevant to Q-Marker properties, including compatibility environment, specificity, measurability, traceability, and effectiveness based on the technical guide of T/CACM 1638-2025 (Table S1). The dataset was stored in CSV format. To improve ranking effectiveness, this study engineered derivative features guided by domain knowledge, including (1) consolidation/standardization of semantically identical variables, (2) synergy captures such as linear combinations (e.g., Effectiveness + Traceability), and (3) a composite indicator Key_Features_Sum (sum of the core metrics) to reflect overall Q-Marker potential. To ensure comparability across features while preserving intra-group ranking structure, feature standardization was conducted within each material group using Standard Scaler (He et al., 2021, Ray, 2019).

The development of machine learning ranking models: To identify and rank Q-Marker in the datasets, this study developed and evaluated five machine learning ranking models, including RankNet, Random Forest, XGBoost, and two baseline models (Linear Regression and K-Nearest Neighbors. Prior to model training, all compounds were grouped by their origin to ensure group-aware learning and evaluation. This study employed 8-Fold GroupKFold Cross-Validation for robust evaluation and to prevent data leakage. Approximately 7/8 of the materials (groups) were used for training, and 1/8 of the materials (groups) served as the test set.

XGBoost, a gradient boosting framework with pairwise ranking objective, was implemented to learn relative rankings of compounds within each herbal material group. The model was optimized using hyperparameters including learning_rate = 0.005, max_depth = 6, and n_estimators = 1 000, with regularization terms to enhance generalization (reg_alpha = 1.0, reg_lambda = 0.05). Random Forest regression models were trained in parallel to provide baseline ranking scores by predicting the Q-Marker label as a continuous outcome. Feature importances from Random Forest were used to assess the relative contribution of each Q-Marker attribute. RankNet, a neural network-based pairwise ranking model, was implemented using Keras. A shared multilayer perceptron network with dense layers (64, 32, and 1 neuron) was applied to pairs of input compounds. The output layer utilized a sigmoid activation function to predict the probability that one compound is a stronger Q-Marker candidate than another. Training pairs were generated within each group based on relative Q-Marker scores, and the model was trained using binary cross-entropy loss. Two baseline models, Linear Regression and K-Nearest Neighbors, were also applied to provide additional reference points for model performance.

Performance evaluation of machine learning ranking models: To systematically evaluate model performance and enhance its predictive power, a comprehensive approach was adopted integrating both ranking metrics and domain-informed feature engineering. The evaluation employed multiple metrics, including normalized discounted cumulative gain (NDCG), which assesses the quality of top-5 rankings by accounting for both relevance and order. In addition, mean average precision (MAP) quantified the accuracy and relevance of the top-ranked compounds, while Recall captured the proportion of true Q-Marker successfully identified within the top-5 predictions. These metrics were computed per material group and averaged across 8-fold cross-validation.

Feature importance evaluation based on SHapley Additive exPlanations (SHAP) analysis: To interpret the internal decision logic of the machine learning models and quantify the relative contribution of each descriptor to Q-Marker identification, SHAP analysis was performed on the trained models. Unlike model-internal importance scores, the SHAP value is based on game theory, assigning a fair contribution to each feature for model output and providing a more unbiased and interpretable feature ranking. In particular, the TreeExplainer module of SHAP was applied to the models. During cross-validation, SHAP values were computed for each test fold and aggregated across all samples to obtain global importance distributions. The SHAP value of each feature was then used to measure its overall contribution to the model output (Ray, 2019).

### 2.3 Characterization of Q-Marker related features of XYHJ

Chemical profile characterization based on ultra-performance liquid chromatography-quadrupole/time-of-flight mass spectrometry (UPLC-Q/TOF-MS): For qualitative analysis, precise amounts of XYHJ (1 mL, equivalent to 1 g of raw herbal mass), XYW powder (1.0 g) and XYKL (3.0 g, equivalent to 2 g of raw herbal mass) were placed in stoppered conical flasks. Each sample was extracted with 4 mL, 10 mL, and 10 mL of distilled water, respectively, followed by ultrasonication (Kun Shan Ultrasonic Instruments Co., Ltd., Kunshan, China) at 25 °C (100 kHz, 250 W) for 10 min to prepare test solutions with a final herbal mass concentration of 0.2 g/mL. The solutions were centrifuged at 4 °C and 14 000 r/min for 10 min using a centrifuge (Thermo Fisher Scientific, Waltham, MA, USA), and the supernatants were analyzed using UPLC-Q/TOF-MS (Waters Corporation, Milford, MA, USA). The detailed information about UPLC-Q/TOF-MS has been listed in Table S9 (Hu et al., 2024).

*In vivo* drug metabolism and pharmacokinetics characterization: The metabolism study was conducted as published previously (Zhu et al., 2025). SPF grade male SD rats were provided by Guangdong Zhiyuan Biopharmaceutical Technology Co., Ltd. (Guangzhou, China, SCXK(YUE)2022-0063). Animals were firstly acclimated for one week under controlled conditions (temperature: 25 °C, humidity: ∼35%, 12-hour light/dark cycle) with ad libitum access to food and water. For *in vivo* xenobiotic analysis, 18 SD rats (200 ± 10 g, seven weeks old) were randomly divided into four groups: XYHJ group (*n* = 5), XYW group (*n* = 5), XYKL group (*n* = 5), and a control group (*n* = 3). Each treatment group received 16.8 g/(kg·d) (based on raw medicinal materials) twice daily for three days, while the control group received an equivalent volume of distilled water. For pharmacokinetics study, another 18 SD rats (200 ± 10 g, seven weeks old) were divided into the same three groups (*n* = 6 each) and received a single dose of 16.8 g/(kg·d) before pharmacokinetic sampling. All the procedures for animal experiments were conducted in accordance with the guidelines approved by the Laboratory Animal Committee of Jinan University (No: 20210616-05).

During dosing, urine and feces were collected continuously; feces were air-dried. Following the final dose, rats underwent bile collection (0−4 h) via common bile duct cannulation, and portal vein blood was sampled at 0.5, 1, 2, and 4 h. Plasma was separated (4 °C, 14 000 r/min, 10 min), pooled (2 mL/group), and precipitated with three volumes of acetonitrile, then dried under nitrogen. Urine (10 mL) and dried feces (1 g) were concentrated, extracted with methanol (24 h soak, 2 h sonication), and dried. All residues (plasma, urine, bile, feces) were reconstituted in water and purified via solid phase extraction (SPE). After washing and methanol elution, eluates were dried, reconstituted in methanol, centrifuged, and analyzed by UPLC-Q/TOF-MS.

The identification of chemical constituents was primarily achieved by direct comparison of retention times, accurate precursor masses (with mass errors typically < 5 × 10^−6^), and MS/MS fragmentation patterns against available authentic reference standards. For compounds without reference standards, tentative annotations were performed based on high-resolution accurate mass data, characteristic fragmentation behaviors, predicted elution order using Clog *P* values, and cross-referencing with in-house TCM compound libraries, published literature, and publicly available databases. Regarding *in vivo* metabolites, prototype compounds were first confirmed using the criteria described above. Subsequent screening for biotransformation products involved comparison of post-dose biological samples with blank controls to exclude endogenous interferences. Potential metabolites were extracted using tools such as mass defect filtering (MDF) and extracted ion chromatography (XIC). Structural elucidation was then accomplished by interpreting changes in accurate masses and MS/MS spectra relative to prototypes, in accordance with common metabolic rules (Phase I reactions: e.g., hydrolysis, oxidation, reduction, hydroxylation; Phase II reactions: e.g., glucuronidation, sulfation, methylation, and glutathione conjugation).

Quantitative analysis of 31 components by ultra-performance liquid chromatography-triple quadrupole tandem mass spectrometry (UPLC-QqQ-MS/MS): For quantitative analysis, 31 components (gallic acid, tryptophan, chlorogenic acid, albiflorin, paeoniflorin, schaftoside, violanthin, neoliquiritin, liquiritin apioside, liquiritin, ferulic acid, pentagalloylglucose, senkyunolide I, lactiflorin, senkyunolide H, ononin, isoliquiritin apioside, isoliquiritin, liquiritigenin, benzoylpaeoniflorin, formonentin, 6-gingerol, saikosaponin A, glycyrrhetinic acid, atractylenolide III, isoliquiritigenin, glycycoumarin, acacetin, ligustilide, atractylenolide II, atractylenolide I) were configured at different concentrations and subjected to methodological investigation using UPLC-QqQ-MS/MS (Waters Corporation, Milford, USA). Precise volumes of 0.2 mL of XYHJ and 0.1 g of XYW powder and 0.3 g of XYKL granules were similarly extracted with 9.8 mL, 10 mL, and 10 mL of distilled water, respectively. The mixtures underwent ultrasonication (25°C, 100 kHz, 250 W, 10 min), cooled to room temperature, and were centrifuged (4 °C, 14 000 r/min, 10 min). The supernatants were then diluted fivefold before analysis using UPLC-QqQ-MS/MS. The detailed information about UPLC-QqQ-MS/MS has been listed in Table S3 (Hu et al., 2024).

For pharmacokinetics study, jugular vein blood (0.5 mL) was collected at 13 time points up to 72 h. Plasma was isolated, and 100 µL was mixed with 50 µL methanol and 50 µL internal standard (Epimedin C, 22.8 ng/mL), followed by protein precipitation with 300 µL methanol. After centrifugation and drying, samples were reconstituted in 100 µL methanol, centrifuged, and analyzed using UPLC-QqQ-MS/MS. The detailed information about LC-MS condition has been listed in Table S11 (Hu et al., 2024).

Development of quantitative analysis method of XYHJ using quantitative analysis of multi-components by single marker (QAMS): This study conducted a detailed investigation of the external standard method to ensure the method’s specificity, linearity and range, intra-day and inter-day precision, accuracy, and stability after 24 h of solution placement, all in accordance with the Chinese Pharmacopoeia (2020 edition) (Hu et al., 2024). This study also explored the feasibility of the QAMS method, including its durability of relative correction factors (RCF), involving factors such as column temperature, detection wavelength, injection volume, mobile phase time program, flow rate, chromatographic column, and mobile phase ratio. Additionally, the mixed control solutions of different gradient concentrations of 15 quantified components were analyzed, choosing pentagalloyl glucose as the internal reference. Using formula (1 and 2), this study calculated the RCF of the internal reference relative to the other 14 quantified components, repeating the process three times to verify the accuracy of the results. Based on both the external standard method and QAMS, this study further conducted quantitative analysis on multiple batches of XYHJ samples, ensuring the accuracy and reliability of the analysis.

(1) $$\mathrm{RCF}=f_{\mathrm{si}}=\frac{f_{s}}{f_{i}}=\frac{A_{s}/C_{s}}{A_{i}/C_{i}}\;$$

(2) $$C_{i}=f_{\mathrm{si}}\times C_{s}\times A_{i}/A_{s}$$

where “*f*” is Relative correction factors, “*s*” is the internal reference standard, “*i*” is one of the other tested components, “*A*” is the peak area of the component under test, and “*C*” is the concentration of the component under test.

Quality transitivity assessment of XYHJ: This study employed the ultra performance liquid chromatography-photodiode array detector (UPLC-PDA) quantitative analysis method to measure the component content at various stages of the XYHJ preparation process (Hu et al., 2024). The samples included individual herbal pieces, intermediates (percolate, decoction, concentrated liquid), and the final product. This study calculated the segmental transfer rates and overall transfer rates for 15 key components to reveal the mass transfer characteristics during the preparation process. Following the prescription and preparation process of XYHJ, this study commissioned the Livzon Group Li Min Pharmaceutical Factory laboratory to prepare six batches of XYHJ final product. XYHJ is prepared from eight herbs via steam distillation, ethanol percolation, and aqueous decoction, followed by combination, concentration, addition of preservatives and volatile oils, and final sterilization. Through further content determination, this study calculated the overall transfer rates for 15 components, i.e., from herbal contributions to the final product, as well as segmental transfer rates, i.e., from herbal contributions to percolation/decoction/(percolation + decoction) liquid (Segment 1), from percolation/decoction/ (percolation + decoction) liquid to concentrated liquid (Segment 2), and from concentrated liquid to XYHJ final product (Segment 3). The formulas for calculating the overall transfer rate and segmental transfer rates are as follows:

(3) $$\text{Overall transfer rate (\%)=}\frac{C_{\text{Finished product}}}{C_{\text{Herbal contribution}}}\;\times \;100$$

(4) $$\text{Segment 1 transfer rate (\%)=}\frac{C_{\text{Extraction liquid}}}{C_{\text{Herbal contribution}}}\;\times \;100$$

(5) $$\text{Segment 2 transfer rate (\%)=}\frac{C_{\text{Concentrated liquid}}}{C_{\text{Extraction liquid}}}\;\times \;100$$

(6) $$\text{Segment 3 transfer rate (\%)=}\frac{C_{\text{Finished product}}}{C_{\text{Concentrated liquid}}}\;\times \;100$$

*C*_Finished product_ refers to the content of the final XYHJ product. C_Herbal contribution_ denotes the content contributed by the herbal materials used. *C*_Extraction liquid_ represents the content in the combined liquids from percolation and decoction processes. *C*_Concentrated liquid_ indicates the content in the concentrated liquid obtained after processing these extracts.

Bioactive evaluation: Mouse-derived BV2 microglial cells were cultured in DMEM supplemented with 10% FBS and maintained at 37 °C with 5% CO_2_. Cells were treated with different concentrations of candidate Q-Marker in the presence of lipopolysaccharide (LPS) to induce inflammation. Nitric oxide (NO) production was measured using the Griess assay. Cell viability was assessed via MTT assays to ensure that inhibitory effects on NO production were not due to cytotoxicity. Dose-response relationships were analyzed to identify bioactive compounds (Hu et al., 2024).

### 2.4 Application of optimized model for Q-Marker identification in XYHJ

An optimized machine learning model was applied to identify potential Q-Marker in the XYHJ formula. Based on results of Q-Marker related features of XYHJ characterization, effectiveness, specificity, traceability, measurability, and compatibility features were collected about chemicals in XYHJ. Specifically, compatibility scoring innovatively applied the TCM “King-Minister-Assistant-Guide” hierarchy. Specificity was assessed via normalized variable importance in projection (VIP) values comparing XYHJ to XYS prescriptions. Measurability was quantified using QAMS-based UPLC component content, traceability combined pharmacokinetic and production quality transfer data, and effectiveness derived from normalized *in vitro* bioactivity assays. The trained and optimized model was selected based on results of NDCG, MAP and Recall, which was then applied to generate ranked predictions of candidate Q-Marker in XYHJ, providing a data-driven basis for further experimental validation and Q-Marker selection (He et al., 2021).

### 2.5 Convergent evidence from complementary computational approaches for Q-Marker prioritization in XYHJ

The multi-dimensional “radar chart” mode construction. According to our previous study (He et al., 2021), data for each dimension were standardized. Three objective weighting methods (entropy weight, standard deviation and principal component analysis) were applied to determine the weights of each feature dimension. Weighted radar charts visualized the feature profiles of candidate Q-Marker. The overall performance of each compound was quantified by calculating the enclosed polygon area, computed as the sum of areas of isosceles triangles formed by adjacent dimension scores and angular spacing. Larger weighted areas indicated more favorable Q-Marker prioritized for further study.

Monte Carlo simulation (MCS): MCS was used to evaluate the robustness of weighted radar chart areas by introducing random variations to factor values, simulating measurement errors (Pan et al., 2024). For each factor *x_j_* of compound *i*, simulated values were generated as $x_{j}^{'}∼N\left(x_{j}\text{, 0.2}\right)$ with values clipped ≥ 0. Weighted areas were calculated using combined weights (Entropy, STD, PCA) with a scaling factor of $\frac{\text{4}}{\text{max}(w_{j})}$, and the area formula. Over 10 000 iterations, the mean area was computed as: $\mu_{\text{Area}}=\frac{1}{N}\sum_{k=1}^{N}{\text{Area}}_{k}^{\prime }$, the standard deviation as: $\sigma_{\text{Area}}=\sqrt{\frac{\text{1}}{N}\sum_{k\text{=1}}^{N}{\left({\text{Area}}_{k}^{'}-\mu_{\text{Area}}\right)}^{2}}$, and the coefficient of variation as: $\mathrm{CV}\text{=}\frac{\sigma_{\text{Area}}}{\mu_{\text{Area}}}\;\times \;100\%$. Here, *N* denotes the total number of Monte Carlo iterations, *k* denotes the iteration index. For compound *i, μ*_Area_ and *σ*_Area_ represent the mean and standard deviation of the simulated radar-chart areas, and CV represents the corresponding coefficient of variation. The 95% confidence interval for each compound was determined from the 2.5th and 97.5th percentiles of the simulated area distribution. The resulting distributions were visualized as histograms and ranked according to their mean weighted areas.

Chemometric evaluation: The fingerprint profiles of XYHJ were constructed using UPLC-PDA (Waters Corporation, Milford, USA), with the detailed testing conditions elaborated in Fig. S5. To assess the contribution of prioritized Q-Marker to the overall quality of XYHJ, similarity analysis was conducted based on the *Traditional Chinese Medicine Chromatographic Fingerprint Evaluation System* (Version 2012). The ranked Q-Marker were designated as common peaks for analysis. Nine batches of XYHJ samples from two manufacturers (Group 1: S1–S3; Group 2: S4–S9) were evaluated for fingerprint similarity scores. Unsupervised chemometric analysis via principal component analysis (PCA) was performed on the peak areas of the key Q-Marker obtained from HPLC data to investigate clustering patterns and batch differentiation between manufacturers (Hu et al., 2024).

### 2.6 Statistical analysis

Statistical analysis was performed using GraphPad Prism 9.0. Data are presented as mean ± standard deviation (SD). Group comparisons were analyzed by One-way analysis of variance‌ (ANOVA), with Student *t*-test for pairwise comparisons. Statistical significance was set at *P* < 0.05. Pharmacokinetic parameters were determined using a non-compartmental model (WinNonlin 6.3).

## 3 Results

### 3.1 Machine learning-driven ranking framework and its performance

To evaluate the predictive effectiveness of the developed framework, five machine learning ranking models: RankNet, Random Forest, XGBoost, and two baseline models (Linear Regression and K-Nearest Neighbors), were applied to rank candidate Q-Marker across the dataset (Fig. S1A). Model performance was evaluated using three widely adopted ranking metrics: NDCG, MAP, and Recall, each calculated at cut-off rank 5, meaning that only the top five ranked candidates for each material were considered when computing the metric values. Across all materials, XGBoost demonstrated the highest overall performance, with average scores of NDCG = 0.89, MAP = 0.95, and Recall = 0.84 (Fig. 2A). Random Forest achieved comparable but slightly lower performance (NDCG = 0.87, MAP = 0.93, Recall = 0.83). In contrast, RankNet exhibited markedly lower predictive accuracy, with average NDCG = 0.46, MAP = 0.81, and Recall = 0.35. Notably, XGBoost demonstrated superior stability, exhibiting low relative standard deviations (RSDs) across multiple evaluation metrics, including NDCG, MAP, and Recall, indicating robust and consistent performance across different validation folds. These findings indicate that tree-based ensemble methods (XGBoost and Random Forest) are better suited for Q-Marker identification in complex, high-dimensional feature spaces typical of TCM formulations (Fig. S1B).

**Fig. 2:**
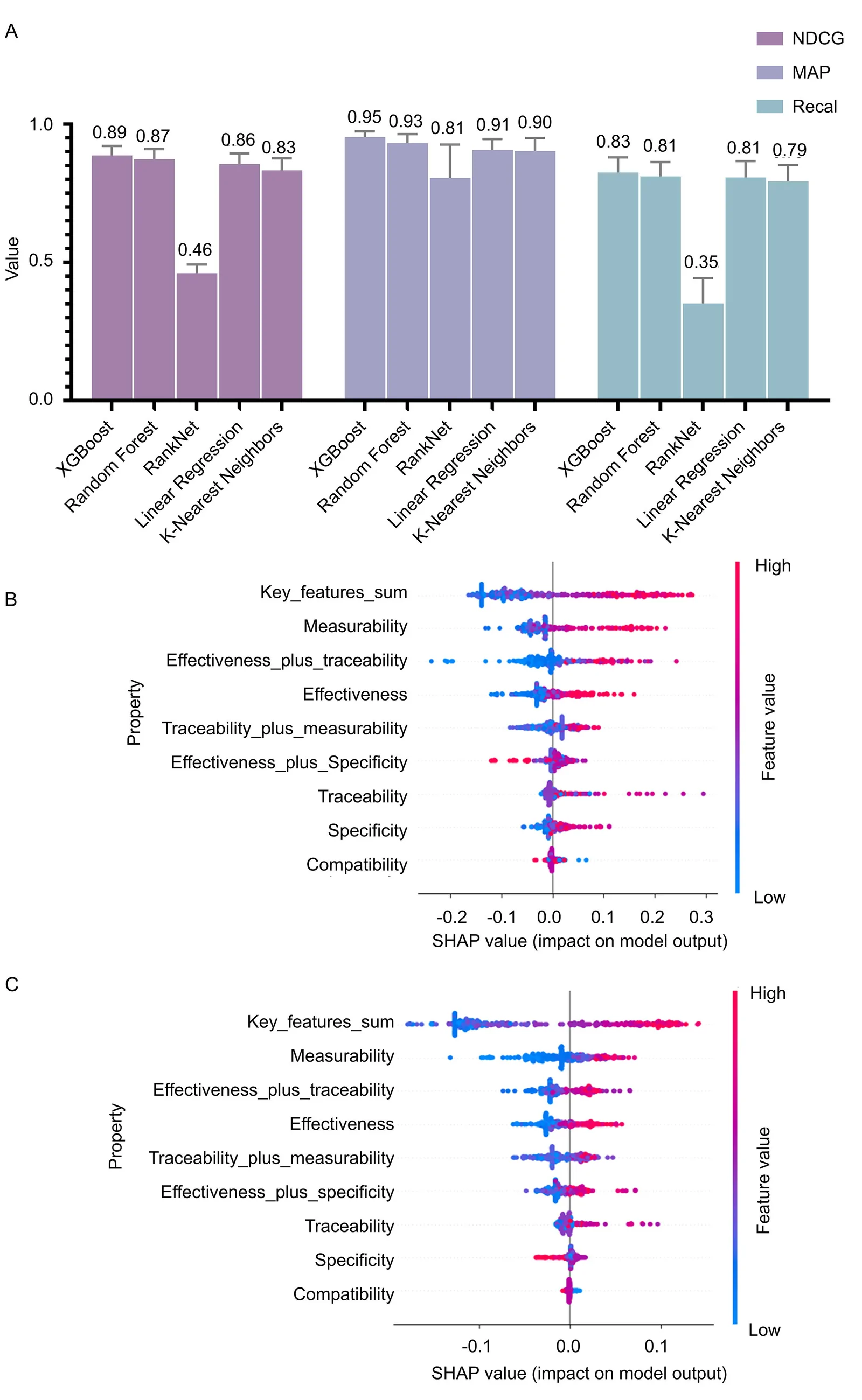
Machine learning-driven workflow for Q-Marker ranking and model optimization. (A) Model performance evaluation using normalized discounted cumulative gain (NDCG), mean average precision (MAP), and Recall metrics for the extreme gradient boosting (XGBoost), Random Forest, RankNet, Linear Regression and K-Nearest Neighbors models. (B) SHapley Additive exPlanations (SHAP) summary plots showing the feature importance in the Random Forest models. (C) SHAP summary plot for the XGBoost models. Red and blue indicate high and low feature values, respectively and Key Features Sum along with specific feature combinations (e.g., Effectiveness + Traceability) emerge as major contributors.

### 3.2 Feature importance analysis

To further elucidate the contribution of each feature to the model’s predictive output, a SHAP analysis was conducted for both the top two models of Random Forest (Fig. 2B) and XGBoost (Fig. 2C). The results consistently show that the composite index, key features sum (XGBoost: 0.085 6; Random Forest: 0.101 2), is the most influential feature across both models. This index, which integrates effectiveness, specificity, measurability, traceability, and compatibility, highlights the critical role of multi-dimensional attribute integration in prediction. Beyond this core feature, other composite indices such as Effectiveness plus Traceability and Traceability plus Measurability also made significant contributions. Among the individual features, Measurability showed moderate importance, emphasizing the practical requirement for quantitative detectability of compounds in analytical workflows. In conclusion, the SHAP analysis consistently demonstrates that integrating pharmacological and quality-related attributes, particularly those emphasizing bioactivity and traceability, is crucial for the accurate identification of Q-Markers.

### 3.3 Qualitative and quantitative comparison of chemical profile of XYHJ and two other XYS prescriptions

A qualitative analysis using UPLC-Q/TOF-MS and quantitative analysis using UPLC-QqQ-MS/MS were conducted to compare the chemical profiles of XYHJ with two commercially available XYS prescriptions—XYW and XYKL. The qualitative analysis results showed that a total of 124 distinct chemical constituents were identified across the three prescriptions, of which 31 compounds were structurally confirmed through reference standards (Table S2). These compounds spanned a diverse range of phytochemicals, including flavonoids, triterpenoids, and organic acids, originating from the eight herbs of XYS. Base peak intensity (BPI) chromatograms revealed that XYHJ contained the most comprehensive chemical profile, with 120 detectable compounds, compared to 118 in XYW and 111 in XYKL (Fig. S2). Notably, 103 constituents (83% of total) were common across all prescriptions.

The quantitative analysis targeted 31 components, including pharmacopoeia-indexed compounds, reported bioactive constituents, and major structural types distributed across the constituent herbs. Multi-batch quantitative analysis of these 31 components indicated that liquiritin apioside, paeoniflorin, gallic acid, glycyrrhizin, and albiflorin were the main components of these prescriptions. The results also showed relative quality stability within batches of the same prescription but differences in component distribution among different prescriptions (Fig. 3A, Table S3). To further investigate these differences, principal component analysis (PCA) confirmed batch-to-batch consistency within each formulation (*R*2*_X_* = 0.892), while orthogonal partial least squares discriminant analysis (OPLS-DA) identified 9 differential markers distinguishing XYHJ from XYW and 7 differential markers distinguishing XYHJ from XYKL (VIP > 1.0) (Fig. 3B−C, S3A−D and S4A−B). These findings suggest that while XYHJ retains the core chemical framework of XYS, its formulation process results in quantitative variations.

**Fig. 3:**
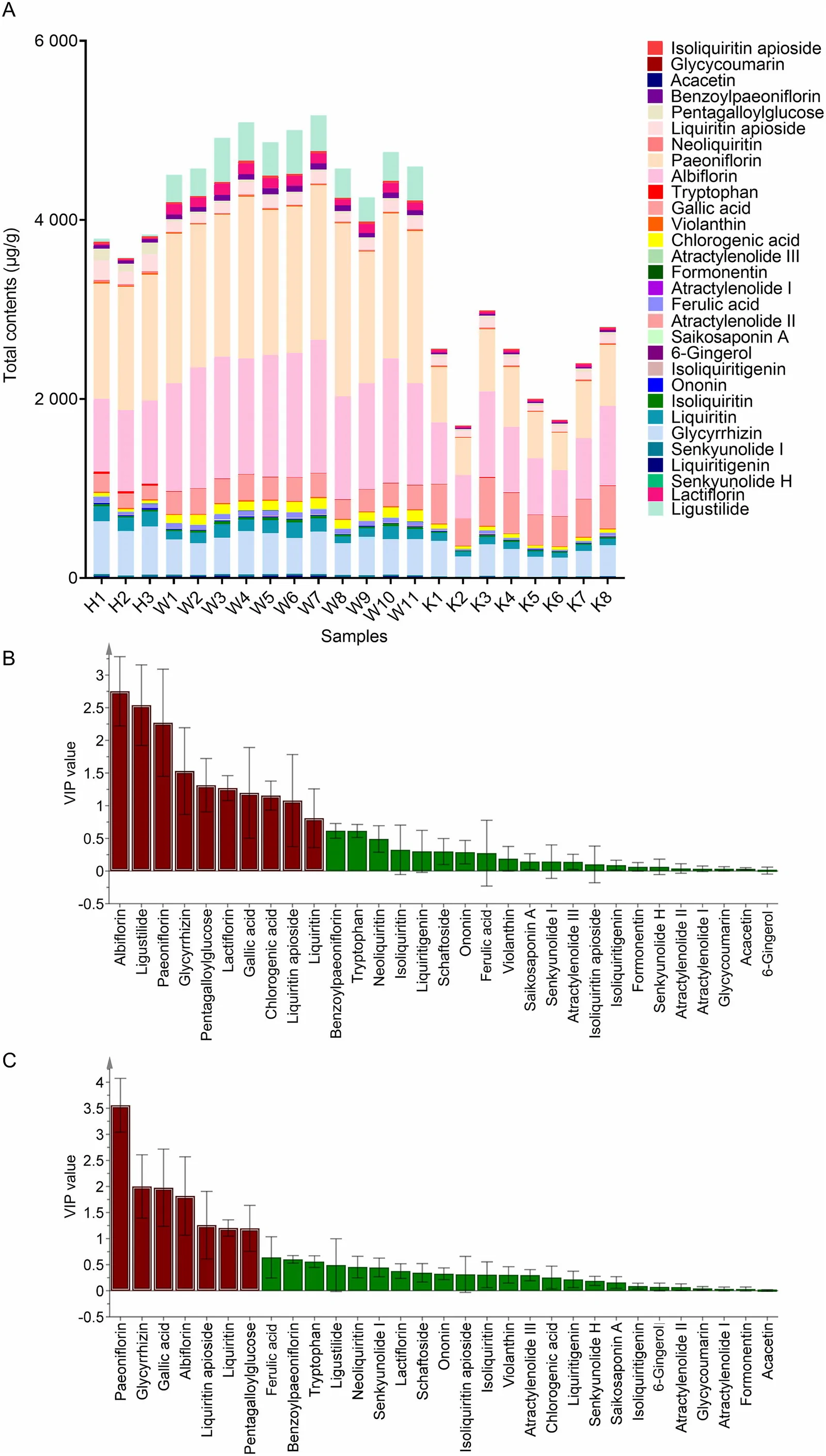
Qualitative and quantitative comparison of chemical components among XYS prescriptions. (A) Overlay diagram of 31 quantified components across 22 batches of XYS prescriptions. H1−H3: 3 batches of XYHJ; W1−W11: 11 batches of XYW; K1−K8: 8 batches of XYKL. (B−C) Variable importance in projection (VIP) plots highlighting differential components between XYHJ *vs* XYW (B) and XYHJ *vs* XYKL (C), with significant marker (VIP > 1.0) indicated in red.

### 3.4 QAMS-based quantitative methodology and its application

To establish a rapid, cost-effective, and accurate method for the simultaneous quantification of multiple components in XYHJ, this study developed a QAMS method using UPLC-PDA. First, based on the chemical profiling and *in vivo* pharmacokinetic characteristics of XYHJ, along with the pharmacopeial indicators of its individual herbs, 15 components were selected for quantitative analysis. To ensure the accuracy, reproducibility, and robustness of the quantitative method, a series of validation experiments were conducted, including specificity, linearity, precision, stability, recovery and accuracy (Fig. S5, Table S4−S7). To assess the feasibility of QAMS, relative correction factors (RCFs) were calculated for each analyte using pentagalloyl glucose as the internal reference. Furthermore, to evaluate the reliability of QAMS, this study compared the quantification results obtained using QAMS and the traditional external standard method across multiple batches of XYHJ. The relative deviations between the two methods remained below 3.5% for all 15 analytes, confirming that QAMS provides a highly accurate alternative to conventional multi-standard calibration methods while significantly reducing analytical cost and time (Table S9).

Using the validated QAMS method, this study quantitatively analyzed multiple batches of XYHJ to assess quality consistency. The results showed that the content of each component ranged as follows: 210.51−246.14 μg/g for gallic acid, 28.65−31.28 μg/g for chlorogenic acid, 771.72−889.22 μg/g for albiflorin, 2 016.87−2 301.75 μg/g for paeoniflorin, 105.90−136.38 μg/g for schaftoside, 18.68−27.20 μg/g for ferulic acid, 109.08−156.85 μg/g for liquiritin apioside, 205.18−258.19 μg/g for liquiritin, 90.44−140.15 μg/g for pentagalloyl glucose, 25.68−38.30 μg/g for senkyunolide I, 307.02−625.89 μg/g for lactiflorin, 7.81−14.95 μg/g for senkyunolide H, 21.28−38.93 μg/g for ononin, 11.50−28.78 μg/g for liquiritigenin, and 573.73−828.48 μg/g for glycyrrhizic acid. Notably, paeoniflorin, paeonolide glycoside, and glycyrrhizic acid were identified as the predominant components (Fig. 4A, Table S8).

**Fig. 4:**
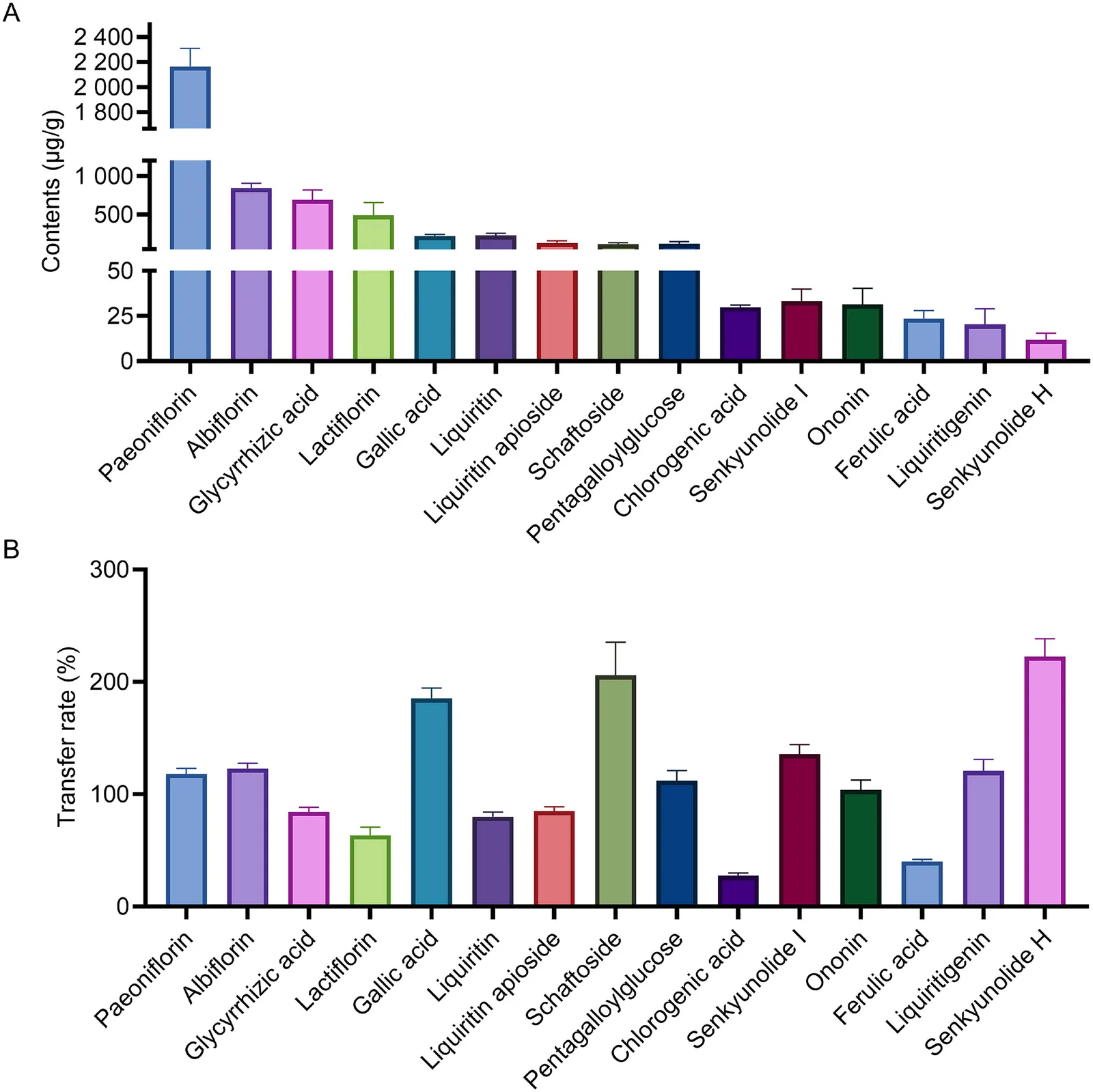
Quantitative analysis and quality transitivity of key compounds in XYHJ. (A) Contents of 15 components in three independent batches of XYHJ. (B) Overall mass transfer efficiency of key components during the XYHJ preparation process. The ApexTrack integration algorithm, which utilizes derivative-based boundary recognition with adaptive thresholds and chromatographic deconvolution, was employed to distinguish overlapping peaks.

### 3.5 Quality transitivity characteristics during preparation of XYHJ

Q-Markers possess traceable characteristics, enabling the confirmation that components contained in compound prescriptions indeed originate from the constituent herbs through their material transfer patterns. They generally must meet three essential requirements: (1) Herb specificity: The component should be a characteristic constituent of the original medicinal material; (2) Preparation stability: It should maintain structural integrity during the formulation process; (3) Quantitative transitivity: A quantifiable transfer pattern must exist from the raw herb to the final preparation (Yang et al., 2017; Zhang et al., 2018). Hence, understanding the mass transfer characteristics of key compounds throughout the manufacturing process is critical for ensuring formulation consistency and optimizing quality control standards.

The study analyzed the 15 quantified components across three stages: from raw herbal slices to percolation/decoction solution, from percolation/decoction solution to concentrated solution, and from concentrated solution to the final product. In the first stage, the transfer rate of components like senkyunolide H reached up to 298.91%, while the transfer rates of chlorogenic acid and ferulic acid were relatively low. In the second stage, the average transfer rate of the 15 components was 77.24%, indicating that the loss of components during sedimentation, filtration, and concentration was minimal. The third stage had an average transfer rate of 108.99%, suggesting that high-temperature sterilization may cause partial solvent evaporation but does not significantly affect the components (Fig. S6). This result implies that while the additional preparation steps may cause some loss of components, they generally improve the dissolution and concentration of active ingredients in XYHJ. The majority of active components were efficiently extracted and retained throughout the entire process (Fig. 4B). It was observed that the transfer rate of these components often exceeded 100% across multiple processing stages, potentially attributable to the conversion of precursor compounds or generation of decomposition products during the processing (Wu et al., 2024). Finally, this study identified gallic acid, albiflorin, paeoniflorin, liquiritin apioside, liquiritin, liquiritigenin, glycyrrhizic acid, senkyunolide I, and ononin as characteristic components influencing the mass transfer in the preparation process of XYHJ (overall transfer rate > 80%). These components demonstrated high transfer rate throughout the preparation process and are critical factors influencing the quality of XYHJ. This study not only reveals the key points of quality control in the preparation process of XYHJ but also provides a scientific basis for further optimization of the preparation process.

### 3.6 Drug metabolism and pharmacokinetic (DMPK) characteristics of XYHJ

The *in vivo* DMPK characteristics of XYHJ were revealed to evaluate the bioavailability features of each prescription. Firstly, this study performed *in vivo* metabolic profiling of prototype compounds and metabolites in plasma, urine, feces, and bile (Table S9 and S10). A total of 38 prototype compounds were identified, with nine detected in plasma, 23 in urine, seven in bile, and 27 in feces. These compounds were primarily flavonoids, monoterpenoids, triterpenoids, lactones, coumarins, and organic acids, mainly derived from *Glycyrrhizae Radix* et *Rhizoma*, *Angelicae Sinensis Radix* and *Paeoniae Radix Alba*. Key exposed compounds included ligustilide, neoliquiritin, glycycoumarin, acacetin, isoliquiritigenin, 18*β*-glycyrrhetinic acid, formononetin, liquiritigenin, isoliquiritin, senkyunolide I, liquiritin, albiflorin, schaftoside, paeoniflorin, and liquiritin apioside, all of which were definitively identified using reference standards. Metabolite profiling revealed a total of 66 metabolites, with 30 detected in plasma, 37 in urine, 12 in bile, and 19 in feces. The major metabolic pathways of XYHJ *in vivo* included hydroxylation, sulfation, glucuronidation, and deglycosylation. Glycyrrhetinic acid, liquiritigenin/isoliquiritin, and ligustilide were identified as major metabolic prototypes.

To assess the pharmacokinetic profiles of key bioactive compounds, this study conducted an *in vivo* pharmacokinetic study, measuring blood concentration-time curves for nine representative compounds (Fig. 5). The pharmacokinetic parameters were calculated using a non-compartmental model (Table S11). The pharmacokinetic analysis of XYHJ revealed substantial variation in the systemic exposure, absorption, and elimination profiles of its major prototype constituents. Among the detected analytes, glycyrrhetinic acid exhibited the highest plasma exposure [*C*_max_ = (1 020.68 ± 349.22) ng/mL; *AUC*_0−∞_ = (39 036.56 ± 7 635.04) ng·h/mL], followed by paeoniflorin [*C*_max_ = (189.27 ± 35.26) ng/mL; *AUC*_0−∞_ = (2 048.18 ± 964.31) ng·h/mL], indicating their dominant contribution to *in vivo* exposure. Liquiritigenin and senkyunolide I also exhibited moderate systemic exposure, with the former showing a prolonged mean residence time (*MRT*_0−∞_ = 80.61 h) suggestive of sustained circulation, while the latter displayed rapid absorption [*T*_max_ = (0.46 ± 0.29) h] and short half-life [*T*_1/2_ = (1.96 ± 0.90) h], indicative of a fast-acting yet transient pharmacokinetic profile.

**Fig. 5:**
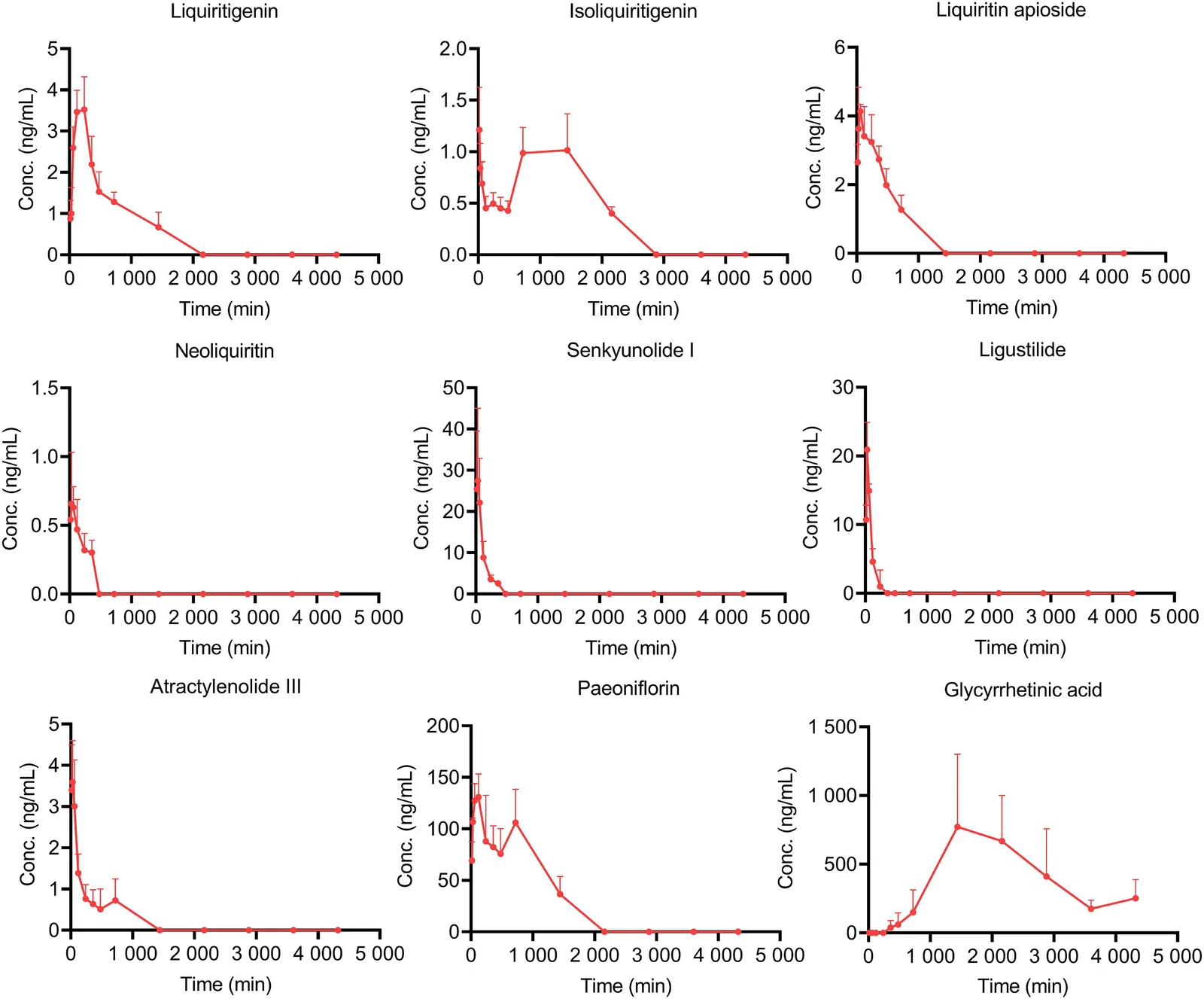
Plasma concentration-time curves of nine representative components in XYHJ, showing their pharmacokinetic profiles following oral administration. Conc. notes concentration.

Notably, the compounds displayed diverse pharmacokinetic behaviors: glycyrrhetinic acid and liquiritigenin exhibited long elimination half-lives [(29.25 ± 16.13) h and (56.25 ± 30.41) h, respectively], while ligustilide and senkyunolide I were rapidly absorbed and eliminated. These findings highlight a dual pattern of fast-acting components with short systemic duration and long-acting constituents with persistent exposure. The observed variability underscores the multi-component, multi-target nature of XYHJ and provides a foundation for further investigation into exposure-effect relationships and Q-Marker prioritization.

### 3.7 Bioactivity assessment of candidate Q-Marker

The efficacy of chemical components stands as the most crucial attribute of Q-Marker (Gu et al., 2025). To further elucidate the pharmacological significance of XYHJ and its candidate Q-Marker, this study conducted a bioactivity assessment using a microglial cell model. As neuroinflammation plays a critical role in the pathogenesis of depression (Lei et al., 2025), investigating the anti-inflammatory effects of key XYHJ constituents provides valuable insights into its therapeutic mechanism, and the cell-based approach allowed for rapid, controlled screening of bioactive constituents. Specifically, we evaluated the ability of key components to suppress nitric oxide (NO) production in lipopolysaccharide (LPS)-activated BV2 microglial cells. The results indicated that all components were well tolerated within the 0–25 µmol/L concentration range, with glycyrrhetinic acid demonstrating no significant cytotoxicity up to 1 mmol/L. These findings confirmed that subsequent anti-inflammatory effects were not attributable to cytotoxicity-induced cell death. NO release measurements indicated that several components, including senkyunolide H, liquiritigenin, paeoniflorin, liquiritin, senkyunolide I, albiflorin, liquiritin apioside, ononin, 18*β*-glycyrrhetinic acid, pentagalloylglucose, gallic acid, chlorogenic acid, schaftoside, ferulic acid, and lactiflorin, inhibited the release of inflammatory factor NO to varying extents in the LPS-induced BV2 cell model (Fig. 6). Among them, senkyunolide H, liquiritigenin, pentagalloylglucose, schaftoside, gallic acid, and chlorogenic acid showed dose-dependent anti-inflammatory effects. Additionally, 18*β*-glycyrrhetinic acid exhibited more pronounced anti-inflammatory activity at low doses. Although pure compounds exhibit lower potency *in vitro* compared with the positive control, this finding may be consistent with the inherently multicomponent and synergistic characteristics of TCM formulations, where the overall therapeutic effect generally arises from the coordinated action of multiple constituents rather than a single dominant compound. Therefore, these results suggest that relevant chemical components of XYHJ may regulate the inflammatory response of the central nervous system. These findings provide important data for further screening of XYHJ Q-Marker and incorporate bioactivity into our proposed multidimensional evaluation strategy.

**Fig. 6:**
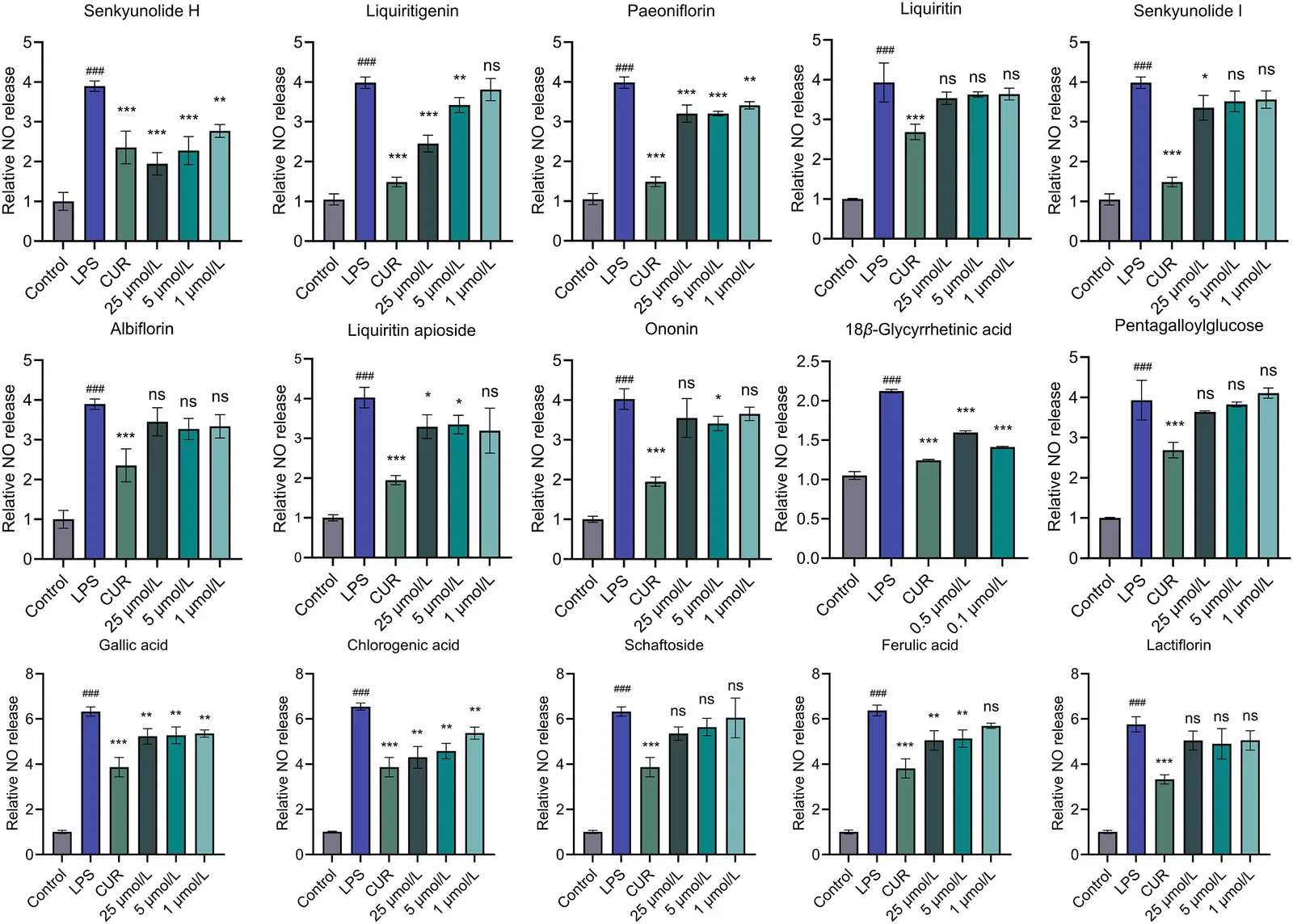
Effects of individual compounds from XYHJ on nitric oxide (NO) release in lipopolysaccharide (LPS)-stimulated BV-2 microglial cells. Curcumin (CUR) served as the positive control. *^###^ P* < 0.001 *vs* control group; *P < 0.05; ^**^P < 0.01; ^***^P < 0.001 *vs* LPS group; ns indicates no significant difference.

### 3.8 Application of optimized model for Q-Marker identification in XYHJ

Based on the characterization of Q-Marker-related features in XYHJ, including effectiveness, specificity, traceability, measurability, and compatibility, an optimized XGBoost model was applied to prioritize its constituent compounds as candidate Q-Marker. The model generated a ranked list of 15 compounds according to their average predicted scores (Fig. 7A−B). Glycyrrhizic acid (0.223 4), Paeoniflorin (0.196 4), and albiflorin (0.038 6) occupied the top three positions, indicating the highest predicted relevance to Q-Marker criteria. The prediction scores for the remaining compounds gradually decreased and were all negative, with ferulic acid ranking last (−0.237 0). Notably, all candidates in this dataset had True Q-Marker labels of zero, indicating that the ranking reflects a purely predictive evaluation without prior empirical designation. These findings demonstrate that the model can effectively differentiate compounds within the same formulation based on integrated pharmacological and analytical attributes, thereby providing a data-driven basis for the experimental validation of top-ranked candidates.

**Fig. 7:**
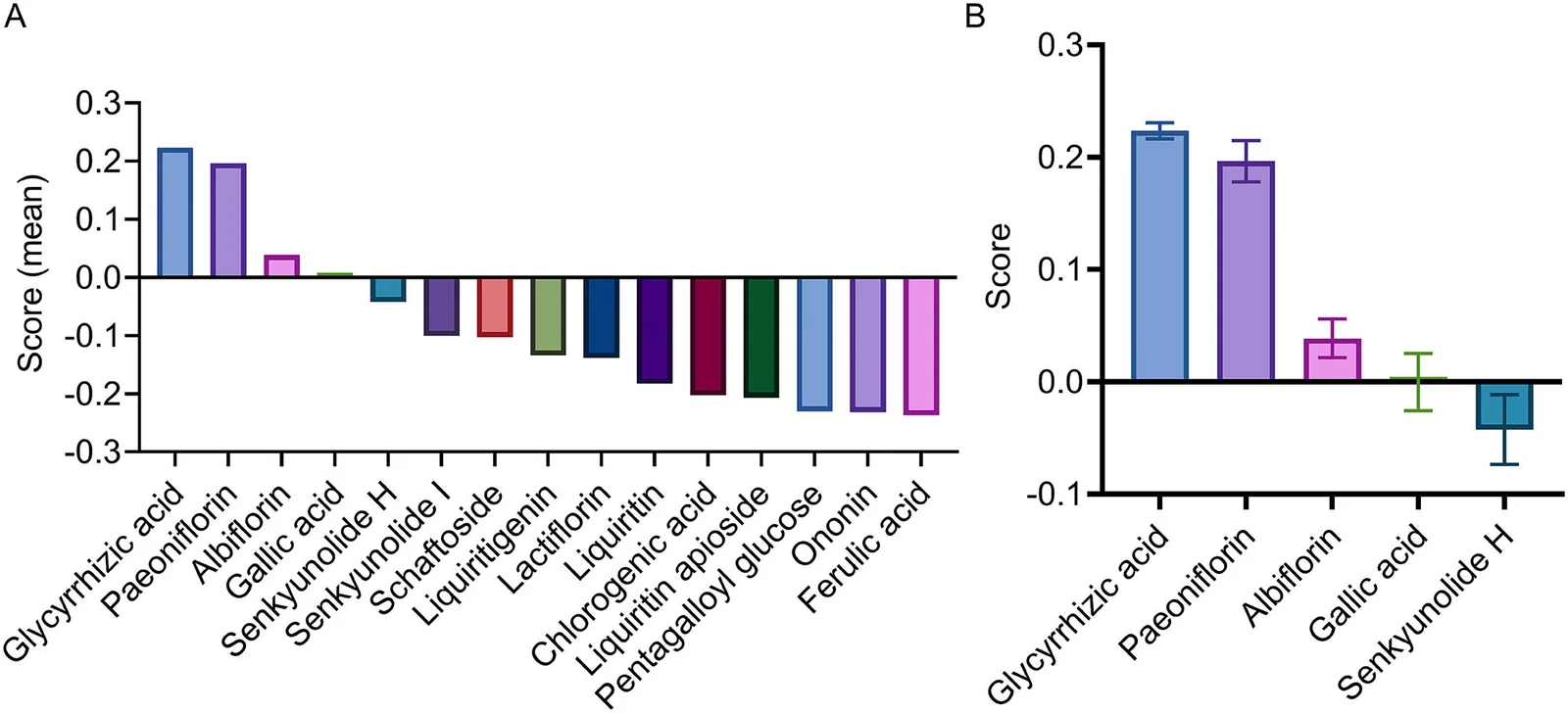
Application of optimized machine learning model, extreme gradient boosting (XGBoost) for Q-Marker identification in XYHJ. (A) Q-Marker ranking in XYHJ generated by the XGBoost model (mean values across multiple models). (B) Ranking performance (Mean ± SD) of the top 5 Q-Markers in XYHJ as predicted by the XGBoost model.

### 3.9 Validation of Q-Marker identified in XYHJ using Monte Carlo simulations

To evaluate candidate compounds in XYHJ and prioritize Q-Marker, a multi-dimensional weighted radar chart model was developed based on our previously published study (He et al., 2021). Each feature was normalized and weighed using three objective methods: entropy, STD, and PCA (Table S12). To reduce bias, weights from these methods were linearly averaged, resulting in final weights of 0.075 5 (compatibility), 0.255 7 (specificity), 0.256 9 (effectiveness), 0.149 6 (traceability), and 0.262 3 (measurability). Using the weighted radar chart area normalized by 4/max (w_j), paeoniflorin ranked highest (193.45), followed by glycyrrhizic acid (182.55) and albiflorin (102.20), indicating superior multi-attribute performance. Liquiritin and senkyunolide I scored moderately, while ononin and ferulic acid ranked lowest (Figs. 8A and S7A). To validate the robustness and reliability of the weighted radar scoring model, we performed Monte Carlo simulations with 10 000 iterations by introducing controlled Gaussian noise (*σ* = 0.2) to the normalized matrix (Fig. 8B). The simulations revealed high stability of the top-ranked compounds: Paeoniflorin maintained the highest mean radar area (193.49 ± 11.90), followed by glycyrrhizic acid (182.56 ± 11.39) and albiflorin (102.23 ± 8.54), with narrow 95% confidence intervals. The relative standard deviations of most compounds were < 10%, indicating strong model robustness under simulated perturbation. Furthermore, sensitivity analysis of individual weighting schemes showed minimal fluctuation (< 0.01) in component weights, particularly for entropy and STD methods, highlighting their resistance to input noise (Fig. 8B). The radar chart scoring system and Monte Carlo simulations further validated the reasonableness of the machine learning results, confirming the model’s reliability and stability for Q-Marker prioritization in TCM quality control.

**Fig. 8:**
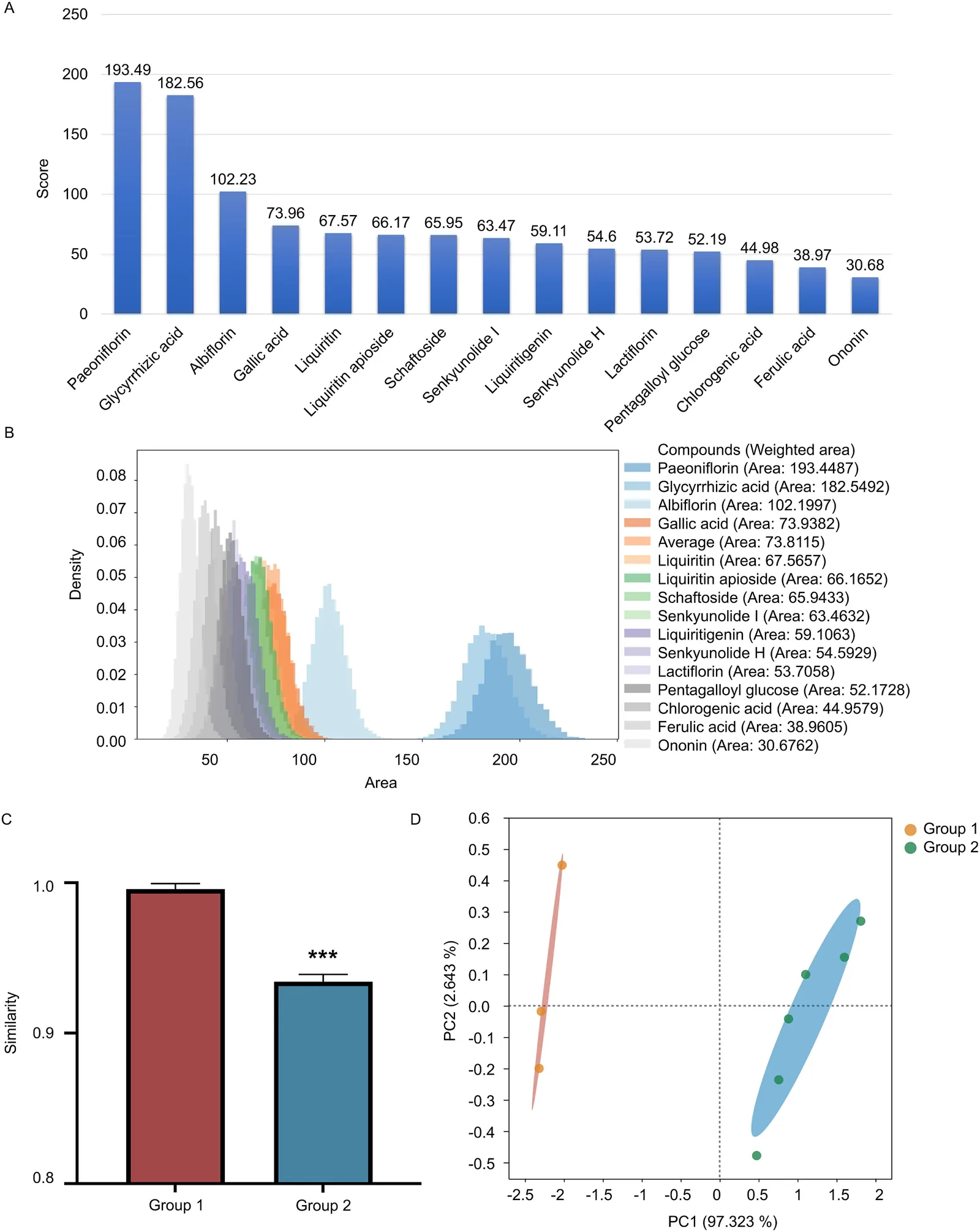
Validation of Q-Markers identified in XYHJ using radar chart modeling, Monte Carlo simulation, and holistic quality evaluation. (A) Ranking of candidate Q-Markers based on radar-chart area scores, highlighting paeoniflorin, albiflorin, and glycyrrhizic acid as key Q-Markers. (B) Monte Carlo simulation results (*n* = 10 000) of 15 candidate Q-Markers further validating the screening outcomes. (C) Comparison of fingerprint similarity between Group 1 and Group 2. (D) Principal component analysis (PCA) score plot based on the peak areas of the three key Q-Markers. ^***^P < 0.001 *vs* Group 1.

### 3.10 Validation of Q-Marker identified in XYHJ using holistic quality evaluation

To evaluate the contribution of Q-Marker to the overall quality of XYHJ, similarity analysis was performed using the TCM chromatographic fingerprint (Version, 2012), with the top three Q-Marker (glycyrrhizic acid, paeoniflorin, and albiflorin) designated as common peaks (Fig. S7B). Similarity analysis revealed that nine batches of XYHJ produced by two manufacturers, Group 1 (S1−S3) and Group 2 (S4−S9), exhibited similarities exceeding 0.9, indicating stable overall quality within each manufacturer. However, while Group 1 samples showed similarities ranging from 0.993−0.999, Group 2 samples exhibited lower similarities, ranging from 0.926−0.940. This suggests that although XYHJ products from different manufacturers maintain a degree of consistency, differences still exist (Fig. 8C). Furthermore, an unsupervised chemometric clustering analysis (principal component analysis, PCA) was conducted using the peak area of the top three Q-Marker derived from HPLC data. PCA clustering analysis revealed distinct separation patterns among samples from the two manufacturers (Fig. 8D), confirming the discriminatory power of prioritized markers. Here, the focus is on a simplified “characteristic chromatogram” aimed at assessing the discriminatory power and practical utility of the selected Q-Markers for routine quality control, rather than full pattern-recognition fingerprinting.

To further assess this discriminatory capability, this study compared the Q-Marker-oriented characteristic fingerprint (using only the top three Q-Markers as common peaks) with a multi-peak fingerprint (using all 15 quantified components as common peaks). Both similarity assessment and PCA clustering produced consistent trends: samples from different manufacturers were effectively distinguished, while intra-manufacturer batches showed strong clustering (Fig. S8A−B). These results indicate that, despite the significant reduction in common peaks, the characteristic fingerprint based on high-confidence Q-Markers retains essential information for quality discrimination.

## 4 Discussion

The identification of representative Q-Markers remains a major challenge in the quality control of TCM prescriptions because their therapeutic effects generally arise from complex chemical constituents with distinct abundance, bioavailability, pharmacological activity, and manufacturing behaviors. Previous Q-Marker identification strategies, including radar-chart models, quantitative ternary networks, network pharmacology, and other multi-criteria approaches, have provided useful tools for candidate prioritization, but differences in evaluation dimensions and weighting schemes may lead to inconsistent conclusions across studies (He et al., 2021, Hu et al., 2024). This study established a machine learning-driven framework by integrating five fundamental Q-Marker attributes (compatibility, specificity, measurability, traceability, and effectiveness) into a unified ranking system. Among the evaluated models, XGBoost showed the best overall ranking performance and stability, suggesting that tree-based ensemble learning is well suited to integrating heterogeneous and potentially nonlinear characteristics associated with Q-Markers.

Application of this framework to XYHJ consistently prioritized paeoniflorin, glycyrrhizic acid, and albiflorin as the leading Q-Marker candidates. Importantly, their prioritization was not determined solely by chemical abundance. Instead, the ranking integrated information from chemical profiling, quantitative analysis, *in vivo* exposure, manufacturing transitivity, and pharmacological activity. The three compounds also remained among the highest-ranked candidates in the weighted radar-chart analysis and Monte Carlo simulations, indicating that their prioritization was robust to changes in computational evaluation. Moreover, the characteristic chromatogram constructed using these three compounds differentiated XYHJ samples produced by different manufacturers, providing additional chemometric evidence that these prioritized Q-Markers capture quality-related variation relevant to practical manufacturing control. Thus, the proposed strategy extends Q-Marker selection from conventional single-dimensional assessment toward an integrated evaluation of chemical, biological, and process-related characteristics.

These findings may also have practical implications for the quality evaluation of XYHJ. Current quality standards for XYS-related preparations rely predominantly on paeoniflorin as a quantitative indicator, whereas a single constituent may not adequately represent the multicomponent nature of TCM prescriptions. The combination of paeoniflorin, glycyrrhizic acid, and albiflorin provides broader chemical representation while maintaining analytical feasibility. In parallel, the QAMS method developed in this study enables simultaneous determination of multiple constituents with reduced dependence on numerous reference standards. Together, machine learning-based prioritization and simplified quantitative analysis may therefore provide a complementary strategy for establishing more informative and practicable quality standards for complex TCM preparations.

Several limitations should nevertheless be acknowledged. Firstly, the training dataset was constructed from 302 compounds collected from previously reported Q-Marker studies, and its coverage and annotation quality may affect model generalizability. External validation using additional TCM prescriptions and independently curated datasets will therefore be necessary. Secondly, the radar-chart and Monte Carlo analyses were based on feature dimensions overlapping with those used for machine learning and should be regarded as convergent rather than completely independent validation. Thirdly, the fingerprint evaluation included only nine batches from two manufacturers, and larger multi-manufacturer and multi-batch datasets are required to confirm the general discriminatory capability of the selected Q-Markers. Finally, the current effectiveness evaluation primarily relied on an in vitro anti-inflammatory model and did not fully capture the potential synergistic or antagonistic interactions among multiple constituents. Future studies integrating *in vivo* pharmacodynamic validation, component-combination effects, and larger real-world quality datasets should further strengthen the biological relevance and applicability of this framework. Despite these limitations, the present study demonstrates the feasibility of integrating machine learning with multidimensional experimental evidence for systematic Q-Marker prioritization and provides a potentially scalable approach for the quality control of TCM prescriptions.

## 5 Conclusion

This study establishes a unified, machine learning-driven framework for the identification and prioritization of Q-Marker in TCM prescriptions. By integrating diverse Q-Marker discovery methods into a comprehensive multi-dimensional feature dataset, and systematically evaluating three ranking models, XGBoost was identified as the optimal tool, demonstrating superior performance and robustness. Applied to XYHJ, the framework enabled the first systematic characterization of its chemical composition, DMPK profiles, quantitative content, manufacturing traceability, and biological efficacy, ultimately identifying key Q-Marker including glycyrrhizic acid, paeoniflorin, and albiflorin. The reliability of these findings was further confirmed through rigorous validation using radar chart modeling combined with extensive Monte Carlo simulations and chemometric analyses. Overall, this machine learning-based approach not only advances the quality control and modernization of XYHJ but also offers a valuable, scalable strategy to standardize Q-Marker screening across diverse TCM prescriptions, promoting their scientific development and clinical application.

## CRediT authorship contribution statement

**Guotao Chen:** Conceptualization, Methodology, Software, Investigation, Data curation, Writing – review & editing. **Jing Hu:** Methodology, Investigation, Data curation. **Aijing Li:** Methodology, Writing – original draft. **Yu Zeng:** Methodology, Investigation, Data curation. **Qingmei Mo:** Investigation, Methodology. **Kairui Tang:** Resources, Validation. **Jiaxu Chen:** Methodology, Project administration. **Xinsheng Yao:** Methodology, Project administration. **Yongxia Guan:** Supervision, Methodology, Writing – review & editing, Project administration. **Guimin Zhang:** Supervision, Methodology, Writing – review & editing, Project administration. **Liangliang He:** Conceptualization, Supervision, Methodology, Writing – review & editing, Project administration. **Zhihong Yao:** Conceptualization, Supervision, Methodology, Writing – review & editing, Project administration.

## Ethics declaration

**Animal subject**

This study was conducted in accordance with the ARRIVE (Animal Research: Reporting of In Vivo Experiments) guidelines.

This study was approved by the Laboratory Animal Committee of Jinan University. (Approval No. 20210616-05)

## Declaration of competing interest

Xinsheng Yao serves as an editorial board member of *Chinese Herbal Medicines* and was not involved in the peer review or publication decision for this article. The authors declare that they have no known competing financial interests or personal relationships that could have appeared to influence the work reported in this paper.
