# Supplementary material for: Discovery of Q-Markers in traditional Chinese medicine based on machine learning: Application and verification in Xiaoyao Heji
Source: Chin Herb Med. 2026 Sep 2;18(4):887–902. doi: 10.1016/j.chmed.2026.08.020 (PMC13712354; doi:10.1016/j.chmed.2026.08.020)
Supplement: Supplementary file 1 [file mmc1.docx]

**Supplementary materials**

**Fig. S1.** (A) Overall workflow for Q-marker ranking and model development. (B) Schematic illustration of the gradient boosting decision tree framework in XGBoost.

**Fig. S2.** Base peak intensity (BPI) chromatograms of XYHJ, XYW, and XYKL in positive (Pos) and negative (Neg) ion mode. A, B and C represent XYHJ, XYW, and XYKL, respectively.

**Fig. S3.** (A−B) Principal component analysis (PCA) score plots showing the compositional clustering of different prescriptions. (C−D) Orthogonal partial least squares discriminant analysis (OPLS-DA) score plots differentiating XYHJ from XYW (C) and XYKL (D).

**Fig. S4.** Permutation test validation of the OPLS-DA models for XYHJ *vs* XYW (A) and XYHJ *vs* XYKL (B).

**Fig. S5.** Chromatograms of 15 quantified compounds. (A) Control solution; (B) Mixed reference solution; (C) XYHJ sample solution; 1: gallic acid; 2: chlorogenic acid; 3: albiflorin; 4: paeoniflorin; 5: schaftoside; 6: ferulic acid; 7: liquiritin apioside; 8: liquiritin; 9: pentagalloyl glucose; 10: senkyunolide I; 11: lactiflorin; 12: senkyunolide H; 13: ononin; 14: liquiritigenin; 15: glycyrrhizic acid. UPLC-PDA analysis was performed using a CORTECS UPLC T3 column (2.1 mm × 150 mm, 1.6 µm) with a flow rate of 0.2 mL/min. The mobile phase consisted of (B) acetonitrile and (A) water (both containing 0.1% formic acid, v/v). The gradient program was: 0–7 min (5%–14% B), 7–14 min (14%–21% B), 14–19 min (21%–27% B), 19–21 min (27%–35% B), 21–24.5 min (35%–80% B), 24.5–25.5 min (80%–100% B), 25.5–26 min (100% B), 26–26.5 min (100%–5% B), and 26.5–28.5 min (5% B). The PDA detector employed a variable wavelength program: 0–6.10 min at 280 nm, 6.10–11.45 min at 250 nm, 11.45–13.00 min at 280 nm, 13.00–14.00 min at 250 nm, 14.00–22.50 min at 280 nm, and 22.50–28.50 min at 260 nm.

**Fig. S6.** Mass transfer efficiency of key bioactive components during different stages of the XYHJ preparation process.

**Fig. S7.** (A) Multi-dimensional weighted radar chart model used for screening Q-markers in XYHJ. (B) Fingerprint analysis of XYHJ samples from two manufacturers (Group 1, S1−S3 and Group 2, S4−S9) using the key Q-markers (albiflorin, paeoniflorin, glycyrrhizic acid) as common peaks (red arrow, from left to right).

**Fig. S8.** (A) A comparison of fingerprint similarity between the two groups; (B) Principal component analysis (PCA) clustering based on the peak areas of 15 quantitative components. **, statistical significance is indicated as *P* < 0.01.

**Table S1** Comprehensive dataset of 302 compounds annotated with multi-dimensional features.

| Names | Materials | Measurability | Specificity | Effectiveness | Traceability | Compatibility | Effectiveness plus traceability | Effectiveness plus specificity | Traceability plus Measurability | Key features sum | Q-marker | References |
| --- | --- | --- | --- | --- | --- | --- | --- | --- | --- | --- | --- | --- |
| Rutin | BZBS | −0.712 789 963 | 0 | 1.153 700 538 | −4.95 × 10^−17^ | −6.18 × 10^−17^ | 1.153 701 | 1.153 701 | −0.712 79 | 0.440 911 | 0 | Tang et al., 2025 |
| Hyperin | BZBS | 0.667 040 705 | 0 | 0.837 363 294 | −4.95 × 10^−17^ | −6.18 × 10^−17^ | 0.837 363 | 0.837 363 | 0.667 041 | 1.504 404 | 1 | Tang et al., 2025 |
| Osthole | BZBS | 1.405 280 718 | 0 | −1.060 660 172 | −4.95 × 10^−17^ | −6.18 × 10^−17^ | −1.060 66 | −1.060 66 | 1.405 281 | 0.344 621 | 1 | Tang et al., 2025 |
| Imperatorin | BZBS | −0.573 695 429 | 0 | −1.376 997 416 | −4.95 × 10^−17^ | −6.18 × 10^−17^ | −1.377 | −1.377 | −0.573 7 | −1.950 69 | 0 | Tang et al., 2025 |
| Isopimpinellin | BZBS | −0.658 698 539 | 0 | −1.060 660 172 | −4.95 × 10^−17^ | −6.18 × 10^−17^ | −1.060 66 | −1.060 66 | −0.658 7 | −1.719 36 | 0 | Tang et al., 2025 |
| Xanthotoxin | BZBS | −0.695 650 81 | 0 | −0.111 648 439 | −4.95 × 10^−17^ | −6.18 × 10^−17^ | −0.111 65 | −0.111 65 | −0.695 65 | −0.807 3 | 0 | Tang et al., 2025 |
| Icaritin | BZBS | −0.740 528 329 | 0 | −0.111 648 439 | −4.95 × 10^−17^ | −6.18 × 10^−17^ | −0.111 65 | −0.111 65 | −0.740 53 | −0.852 18 | 0 | Tang et al., 2025 |
| Icariin | BZBS | 0.625 449 265 | 0 | 1.153 700 538 | −4.95 × 10^−17^ | −6.18 × 10^−17^ | 1.153 701 | 1.153 701 | 0.625 449 | 1.779 15 | 1 | Tang et al., 2025 |
| Epimedin C | BZBS | 1.629 571 662 | 0 | 1.470 037 782 | −4.95 × 10^−17^ | −6.18 × 10^−17^ | 1.470 038 | 1.470 038 | 1.629 572 | 3.099 609 | 1 | Tang et al., 2025 |
| Icariside II | BZBS | −0.454 301 255 | 0 | 1.470 037 782 | −4.95 × 10^−17^ | −6.18 × 10^−17^ | 1.470 038 | 1.470 038 | −0.454 3 | 1.015 737 | 0 | Tang et al., 2025 |
| Schisantherin B | BZBS | −0.641 494 953 | 0 | −0.427 985 683 | −4.95 × 10^−17^ | −6.18 × 10^−17^ | −0.427 99 | −0.427 99 | −0.641 49 | −1.069 48 | 0 | Tang et al., 2025 |
| Schisandrin | BZBS | −0.204 929 803 | 0 | −0.427 985 683 | −4.95 × 10^−17^ | −6.18 × 10^−17^ | −0.427 99 | −0.427 99 | −0.204 93 | −0.632 92 | 1 | Tang et al., 2025 |
| Schisantherin A | BZBS | −0.761 082 426 | 0 | −0.427 985 683 | −4.95 × 10^−17^ | −6.18 × 10^−17^ | −0.427 99 | −0.427 99 | −0.761 08 | −1.189 07 | 0 | Tang et al., 2025 |
| Schisandrin B | BZBS | −0.436 694 964 | 0 | −1.060 660 172 | −4.95 × 10^−17^ | −6.18 × 10^−17^ | −1.060 66 | −1.060 66 | −0.436 69 | −1.497 36 | 0 | Tang et al., 2025 |
| Magnoflorine | BZBS | −0.377 062 31 | 0 | −0.427 985 683 | −4.95 × 10^−17^ | −6.18 × 10^−17^ | −0.427 99 | −0.427 99 | −0.377 06 | −0.805 05 | 1 | Tang et al., 2025 |
| Cuscutamine | BZBS | 2.667 215 278 | 0 | −1.060 660 172 | −4.95 × 10^−17^ | −6.18 × 10^−17^ | −1.060 66 | −1.060 66 | 2.667 215 | 1.606 555 | 1 | Tang et al., 2025 |
| Hydroxyasiatic acid | BZBS | −0.737 628 848 | 0 | 1.470 037 782 | −4.95 × 10^−17^ | −6.18 × 10^−17^ | 1.470 038 | 1.470 038 | −0.737 63 | 0.732 409 | 1 | Tang et al., 2025 |
| Hesperidin | BZYQT | 3.167 569 17 | −8.84 × 10^18^ | −0.640 422 523 | −0.325 368 339 | −6.18 × 10^−17^ | −0.965 79 | −0.640 42 | 2.842 201 | 2.201 778 | 1 | Hu et al., 2024 |
| Astragaloside IV | BZYQT | −0.525 933 19 | −8.84 × 10^18^ | 0.841 949 445 | 1.727 872 555 | −6.18 × 10^−17^ | 2.569 822 | 0.841 949 | 1.201 939 | 2.043 889 | 1 | Hu et al., 2024 |
| Ononin | BZYQT | −0.426 432 316 | −8.84 × 10^18^ | 0.132 498 351 | 2.317 390 379 | −6.18 × 10^−17^ | 2.449 889 | 0.132 498 | 1.890 958 | 2.023 456 | 1 | Hu et al., 2024 |
| Glycyrrhizic acid | BZYQT | 1.923 434 184 | −8.84 × 10^18^ | −0.716 586 259 | −0.279 710 922 | −6.18 × 10^−17^ | −0.996 3 | −0.716 59 | 1.643 723 | 0.927 137 | 1 | Hu et al., 2024 |
| Narirutin | BZYQT | 0.402 492 256 | −8.84 × 10^18^ | −0.472 580 217 | −0.289 110 979 | −6.18 × 10^−17^ | −0.761 69 | −0.472 58 | 0.113 381 | −0.359 2 | 1 | Hu et al., 2024 |
| Calycosin | BZYQT | −0.462 342 406 | −8.84 × 10^18^ | 2.844 773 608 | −0.606 027 167 | −6.18 × 10^−17^ | 2.238 746 | 2.844 774 | −1.068 37 | 1.776 404 | 1 | Hu et al., 2024 |
| Cimigenoside | BZYQT | −0.488 526 847 | −8.84 × 10^18^ | 0.066 207 692 | 0.397 093 14 | −6.18 × 10^−17^ | 0.463 301 | 0.066 208 | −0.091 43 | −0.025 23 | 1 | Hu et al., 2024 |
| Astragaloside II | BZYQT | −0.432 417 331 | −8.84 × 10^18^ | −0.586 825 82 | 2.017 931 439 | −6.18 × 10^−17^ | 1.431 106 | −0.586 83 | 1.585 514 | 0.998 688 | 1 | Hu et al., 2024 |
| Liquiritin | BZYQT | 0.509 474 399 | −8.84 × 10^18^ | −0.424 625 272 | −0.647 655 988 | −6.18 × 10^−17^ | −1.072 28 | −0.424 63 | −0.138 18 | −0.562 81 | 1 | Hu et al., 2024 |
| Liquiritigenin | BZYQT | −0.451 120 503 | −8.84 × 10^18^ | 0.311 624 174 | −0.110 509 907 | −6.18 × 10^−17^ | 0.201 114 | 0.311 624 | −0.561 63 | −0.250 01 | 0 | Hu et al., 2024 |
| Formononetin | BZYQT | −0.461 594 279 | −8.84 × 10^18^ | 2.052 106 58 | −0.928 314 815 | −6.18 × 10^−17^ | 1.123 792 | 2.052 107 | −1.389 91 | 0.662 197 | 0 | Hu et al., 2024 |
| Calycosin-7-*O*-*β*-*D*-glucoside | BZYQT | −0.283 540 084 | −8.84 × 10^18^ | −0.835 063 181 | −0.032 623 725 | −6.18 × 10^−17^ | −0.867 69 | −0.835 06 | −0.316 16 | −1.151 23 | 0 | Hu et al., 2024 |
| Saikosaponin A | BZYQT | −0.347 878 995 | −8.84 × 10^18^ | −0.739 153 292 | −0.174 967 437 | −6.18 × 10^−17^ | −0.914 12 | −0.739 15 | −0.522 85 | −1.262 | 0 | Hu et al., 2024 |
| Magnoflorine | BZYQT | −0.554 362 011 | −8.84 × 10^18^ | −0.417 573 074 | −0.619 455 819 | −6.18 × 10^−17^ | −1.037 03 | −0.417 57 | −1.173 82 | −1.591 39 | 0 | Hu et al., 2024 |
| Isoliquiritigenin | BZYQT | −0.540 147 601 | −8.84 × 10^18^ | −0.596 698 897 | −0.591 255 649 | −6.18 × 10^−17^ | −1.187 95 | −0.596 7 | −1.131 4 | −1.728 1 | 0 | Hu et al., 2024 |
| Heptamethoxy flavone | BZYQT | −0.488 526 847 | −8.84 × 10^18^ | −0.201 775 823 | −0.928 314 815 | −6.18 × 10^−17^ | −1.130 09 | −0.201 78 | −1.416 84 | −1.618 62 | 0 | Hu et al., 2024 |
| Atractylenolide III | BZYQT | −0.540 147 601 | −8.84 × 10^18^ | −0.617 855 49 | −0.926 971 95 | −6.18 × 10^−17^ | −1.544 83 | −0.617 86 | −1.467 12 | −2.084 98 | 0 | Hu et al., 2024 |
| Puerarin | DLP | 2.991 234 38 | −8.84 × 10^18^ | 1.055 929 752 | 0.795 742 423 | −6.18 × 10^−17^ | 1.851 672 | 1.055 93 | 3.786 977 | 4.842 907 | 1 | Wang et al., 2023 |
| Mirificin | DLP | 0.786 593 395 | −8.84 × 10^18^ | −0.965 582 165 | −0.158 050 961 | −6.18 × 10^−17^ | −1.123 63 | −0.965 58 | 0.628 542 | −0.337 04 | 0 | Wang et al., 2023 |
| Methoxypuerarin | DLP | 0.564 949 509 | −8.84 × 10^18^ | −1.603 352 693 | −0.168 312 057 | −6.18 × 10^−17^ | −1.771 66 | −1.603 35 | 0.396 637 | −1.206 72 | 0 | Wang et al., 2023 |
| Paeoniflorin | DLP | 0.317 259 904 | −8.84 × 10^18^ | 1.108 567 753 | 3.269 155 386 | −6.18 × 10^−17^ | 4.377 723 | 1.108 568 | 3.586 415 | 4.694 983 | 1 | Wang et al., 2023 |
| Daidzin | DLP | 0.166 852 152 | −8.84 × 10^18^ | −1.390 939 749 | −0.456 600 016 | −6.18 × 10^−17^ | −1.847 54 | −1.390 94 | −0.289 75 | −1.680 69 | 0 | Wang et al., 2023 |
| Ononin | DLP | −0.632 619 891 | −8.84 × 10^18^ | −1.071 389 865 | −0.511 325 866 | −6.18 × 10^−17^ | −1.582 72 | −1.071 39 | −1.143 95 | −2.215 34 | 0 | Wang et al., 2023 |
| Daidzein | DLP | −0.416 131 858 | −8.84 × 10^18^ | 1.055 929 752 | −0.295 354 209 | −6.18 × 10^−17^ | 0.760 576 | 1.055 93 | −0.711 49 | 0.344 444 | 1 | Wang et al., 2023 |
| Formononetin | DLP | −0.714 333 205 | −8.84 × 10^18^ | −0.247 791 243 | −0.506 928 253 | −6.18 × 10^−17^ | −0.754 72 | −0.247 79 | −1.221 26 | −1.469 05 | 0 | Wang et al., 2023 |
| Alisol A | DLP | −0.502 309 073 | −8.84 × 10^18^ | 1.321 778 242 | −0.267 991 284 | −6.18 × 10^−17^ | 1.053 787 | 1.321 778 | −0.770 3 | 0.551 478 | 1 | Wang et al., 2023 |
| TanshinoneI | DLP | −0.611 545 539 | −8.84 × 10^18^ | −0.619 979 129 | −0.313 921 908 | −6.18 × 10^−17^ | −0.933 9 | −0.619 98 | −0.925 47 | −1.545 45 | 0 | Wang et al., 2023 |
| Cryptotanshinone | DLP | −0.587 750 656 | −8.84 × 10^18^ | 0.150 981 492 | −0.478 588 081 | −6.18 × 10^−17^ | −0.327 61 | 0.150 981 | −1.066 34 | −0.915 36 | 0 | Wang et al., 2023 |
| Levistolide A | DLP | −0.719 402 982 | −8.84 × 10^18^ | 0.523 169 378 | −0.469 304 232 | −6.18 × 10^−17^ | 0.053 865 | 0.523 169 | −1.188 71 | −0.665 54 | 0 | Wang et al., 2023 |
| Tanshinone IIA | DLP | −0.642 796 136 | −8.84 × 10^18^ | 0.682 678 472 | −0.438 520 941 | −6.18 × 10^−17^ | 0.244 158 | 0.682 678 | −1.081 32 | −0.398 64 | 1 | Wang et al., 2023 |
| Kaempferol | *Eupatorium lindleyanum* | −0.161 249 242 | 1.544 796 999 | 1.407 360 081 | −4.95 × 10^−17^ | −6.18 × 10^−17^ | 1.407 36 | 2.952 157 | −0.161 25 | 2.790 908 | 1 | Huang et al., 2021a |
| Eupalinolide A | *Eupatorium lindleyanum* | −0.270 372 596 | −0.524 006 031 | 1.406 434 629 | −4.95 × 10^−17^ | −6.18 × 10^−17^ | 1.406 435 | 0.882 429 | −0.270 37 | 0.612 056 | 1 | Huang et al., 2021a |
| Eupalinolide K | *Eupatorium lindleyanum* | 1.756 203 982 | −0.524 006 031 | 1.384 849 098 | −4.95 × 10^−17^ | −6.18 × 10^−17^ | 1.384 849 | 0.860 843 | 1.756 204 | 2.617 047 | 1 | Huang et al., 2021a |
| Isoquercitrin | *Eupatorium lindleyanum* | −0.395 085 001 | 0.829 271 139 | 1.367 965 861 | −4.95 × 10^−17^ | −6.18 × 10^−17^ | 1.367 966 | 2.197 237 | −0.395 09 | 1.802 152 | 1 | Huang et al., 2021a |
| Eupafolin | *Eupatorium lindleyanum* | 0.041 408 416 | −0.430 676 571 | 1.363 763 81 | −4.95 × 10^−17^ | −6.18 × 10^−17^ | 1.363 764 | 0.933 087 | 0.041 408 | 0.974 496 | 1 | Huang et al., 2021a |
| Luteolin | *Eupatorium lindleyanum* | −0.395 085 001 | 0.938 155 509 | 1.362 363 127 | −4.95 × 10^−17^ | −6.18 × 10^−17^ | 1.362 363 | 2.300 519 | −0.395 09 | 1.905 434 | 1 | Huang et al., 2021a |
| Cirsiliol | *Eupatorium lindleyanum* | −0.395 085 001 | −0.477 341 301 | 1.352 033 087 | −4.95 × 10^−17^ | −6.18 × 10^−17^ | 1.352 033 | 0.874 692 | −0.395 09 | 0.479 607 | 1 | Huang et al., 2021a |
| Eupalinolide B | *Eupatorium lindleyanum* | 2.223 875 5 | −0.524 006 031 | 1.350 632 404 | −4.95 × 10^−17^ | −6.18 × 10^−17^ | 1.350 632 | 0.826 626 | 2.223 876 | 3.050 502 | 1 | Huang et al., 2021a |
| Eupalinolide C | *Eupatorium lindleyanum* | −0.395 085 001 | −0.524 006 031 | 1.328 596 653 | −4.95 × 10^−17^ | −6.18 × 10^−17^ | 1.328 597 | 0.804 591 | −0.395 09 | 0.409 506 | 1 | Huang et al., 2021a |
| Scoparone | *Eupatorium lindleyanum* | −0.395 085 001 | −0.290 682 381 | 1.181 374 824 | −4.95 × 10^−17^ | −6.18 × 10^−17^ | 1.181 375 | 0.890 692 | −0.395 09 | 0.495 607 | 0 | Huang et al., 2021a |
| Hyperoside | *Eupatorium lindleyanum* | −0.098 893 04 | 0.767 051 499 | 1.151 360 18 | −4.95 × 10^−17^ | −6.18 × 10^−17^ | 1.151 36 | 1.918 412 | −0.098 89 | 1.819 519 | 1 | Huang et al., 2021a |
| Adenosine | *Eupatorium lindleyanum* | −0.395 085 001 | −0.212 907 831 | 1.121 370 548 | −4.95 × 10^−17^ | −6.18 × 10^−17^ | 1.121 371 | 0.908 463 | −0.395 09 | 0.513 378 | 0 | Huang et al., 2021a |
| Scopoletin | *Eupatorium lindleyanum* | −0.395 085 001 | 0.658 167 129 | 1.091 355 904 | −4.95 × 10^−17^ | −6.18 × 10^−17^ | 1.091 356 | 1.749 523 | −0.395 09 | 1.354 438 | 0 | Huang et al., 2021a |
| *β*-Sitosterol | *Eupatorium lindleyanum* | −0.395 085 001 | 3.286 946 919 | 1.087 604 074 | −4.95 × 10^−17^ | −6.18 × 10^−17^ | 1.087 604 | 4.374 551 | −0.395 09 | 3.979 466 | 0 | Huang et al., 2021a |
| Caffeicacid | *Eupatorium lindleyanum* | −0.114 482 09 | −0.321 792 201 | 1.062 766 956 | −4.95 × 10^−17^ | −6.18 × 10^−17^ | 1.062 767 | 0.740 975 | −0.114 48 | 0.626 493 | 1 | Huang et al., 2021a |
| Astragalin | *Eupatorium lindleyanum* | −0.176 838 293 | 0.564 837 669 | 1.021 496 82 | −4.95 × 10^−17^ | −6.18 × 10^−17^ | 1.021 497 | 1.586 334 | −0.176 84 | 1.409 496 | 0 | Huang et al., 2021a |
| Rutin | *Eupatorium lindleyanum* | −0.145 660 192 | 0.844 826 049 | 0.993 833 323 | −4.95 × 10^−17^ | −6.18 × 10^−17^ | 0.993 833 | 1.838 659 | −0.145 66 | 1.692 999 | 1 | Huang et al., 2021a |
| Octacosanoic acid | *Eupatorium lindleyanum* | −0.395 085 001 | −0.352 902 021 | 0.938 981 561 | −4.95 × 10^−17^ | −6.18 × 10^−17^ | 0.938 982 | 0.586 08 | −0.395 09 | 0.190 995 | 0 | Huang et al., 2021a |
| Vanillic acid | *Eupatorium lindleyanum* | −0.270 372 596 | 0.782 606 409 | 0.912 718 748 | −4.95 × 10^−17^ | −6.18 × 10^−17^ | 0.912 719 | 1.695 325 | −0.270 37 | 1.424 953 | 0 | Huang et al., 2021a |
| Daucosterol | *Eupatorium lindleyanum* | −0.395 085 001 | 3.333 611 649 | 0.883 654 568 | −4.95 × 10^−17^ | −6.18 × 10^−17^ | 0.883 655 | 4.217 266 | −0.395 09 | 3.822 181 | 0 | Huang et al., 2021a |
| 5-Hydroxymethylfurfural | *Eupatorium lindleyanum* | −0.395 085 001 | 0.098 190 369 | 0.882 253 884 | −4.95 × 10^−17^ | −6.18 × 10^−17^ | 0.882 254 | 0.980 444 | −0.395 09 | 0.585 359 | 0 | Huang et al., 2021a |
| Taraxasterolacetate | *Eupatorium lindleyanum* | −0.395 085 001 | −0.384 011 841 | 0.855 991 071 | −4.95 × 10^−17^ | −6.18 × 10^−17^ | 0.855 991 | 0.471 979 | −0.395 09 | 0.076 894 | 0 | Huang et al., 2021a |
| Buddleoside | *Eupatorium lindleyanum* | −0.395 085 001 | −0.290 682 381 | 0.851 313 789 | −4.95 × 10^−17^ | −6.18 × 10^−17^ | 0.851 314 | 0.560 631 | −0.395 09 | 0.165 546 | 0 | Huang et al., 2021a |
| *p*-Hydroxybenzaldehyde | *Eupatorium lindleyanum* | −0.395 085 001 | −0.337 347 111 | 0.799 738 626 | −4.95 × 10^−17^ | −6.18 × 10^−17^ | 0.799 739 | 0.462 392 | −0.395 09 | 0.067 307 | 0 | Huang et al., 2021a |
| Coniferylalcohol | *Eupatorium lindleyanum* | −0.395 085 001 | 0.098 190 369 | 0.739 709 338 | −4.95 × 10^−17^ | −6.18 × 10^−17^ | 0.739 709 | 0.837 9 | −0.395 09 | 0.442 815 | 0 | Huang et al., 2021a |
| Trifolin | *Eupatorium lindleyanum* | 0.056 997 466 | −0.088 468 551 | 0.645 013 136 | −4.95 × 10^−17^ | −6.18 × 10^−17^ | 0.645 013 | 0.556 545 | 0.056 997 | 0.613 542 | 0 | Huang et al., 2021a |
| Quercetin | *Eupatorium lindleyanum* | −0.036 536 837 | 3.426 941 109 | 0.637 509 475 | −4.95 × 10^−17^ | −6.18 × 10^−17^ | 0.637 509 | 4.064 451 | −0.036 54 | 4.027 914 | 1 | Huang et al., 2021a |
| Palmiticacid | *Eupatorium lindleyanum* | −0.395 085 001 | 3.209 172 369 | 0.637 034 243 | −4.95 × 10^−17^ | −6.18 × 10^−17^ | 0.637 034 | 3.846 207 | −0.395 09 | 3.451 122 | 0 | Huang et al., 2021a |
| Eupatilin | *Eupatorium lindleyanum* | −0.348 317 849 | −0.430 676 571 | 0.499 667 222 | −4.95 × 10^−17^ | −6.18 × 10^−17^ | 0.499 667 | 0.068 991 | −0.348 32 | −0.279 33 | 0 | Huang et al., 2021a |
| Jaceosidin | *Eupatorium lindleyanum* | 0.399 956 579 | −0.446 231 481 | 0.402 619 874 | −4.95 × 10^−17^ | −6.18 × 10^−17^ | 0.402 62 | −0.043 61 | 0.399 957 | 0.356 345 | 0 | Huang et al., 2021a |
| Medioresinol | *Eupatorium lindleyanum* | −0.395 085 001 | −0.461 786 391 | 0.366 052 032 | −4.95 × 10^−17^ | −6.18 × 10^−17^ | 0.366 052 | −0.095 73 | −0.395 09 | −0.490 82 | 0 | Huang et al., 2021a |
| Taraxasterol | *Eupatorium lindleyanum* | −0.395 085 001 | −0.461 786 391 | −0.161 880 543 | −4.95 × 10^−17^ | −6.18 × 10^−17^ | −0.161 88 | −0.623 67 | −0.395 09 | −1.018 75 | 0 | Huang et al., 2021a |
| Eupalinolide F | *Eupatorium lindleyanum* | −0.067 714 939 | −0.524 006 031 | −0.966 423 075 | −4.95 × 10^−17^ | −6.18 × 10^−17^ | −0.966 42 | −1.490 43 | −0.067 71 | −1.558 14 | 0 | Huang et al., 2021a |
| Eupalinolide G | *Eupatorium lindleyanum* | 2.301 820 753 | −0.524 006 031 | −0.966 423 075 | −4.95 × 10^−17^ | −6.18 × 10^−17^ | −0.966 42 | −1.490 43 | 2.301 821 | 0.811 392 | 0 | Huang et al., 2021a |
| Eupalinolide H | *Eupatorium lindleyanum* | 0.166 120 82 | −0.524 006 031 | −0.966 423 075 | −4.95 × 10^−17^ | −6.18 × 10^−17^ | −0.966 42 | −1.490 43 | 0.166 121 | −1.324 31 | 0 | Huang et al., 2021a |
| EupalinolideI | *Eupatorium lindleyanum* | −0.114 482 09 | −0.524 006 031 | −0.966 423 075 | −4.95 × 10^−17^ | −6.18 × 10^−17^ | −0.966 42 | −1.490 43 | −0.114 48 | −1.604 91 | 0 | Huang et al., 2021a |
| Eupalinolide O | *Eupatorium lindleyanum* | 0.134 942 719 | −0.524 006 031 | −0.966 423 075 | −4.95 × 10^−17^ | −6.18 × 10^−17^ | −0.966 42 | −1.490 43 | 0.134 943 | −1.355 49 | 0 | Huang et al., 2021a |
| Chlorogenic acid | *Eupatorium lindleyanum* | 3.034 506 131 | 1.638 126 459 | −0.966 423 075 | −4.95 × 10^−17^ | −6.18 × 10^−17^ | −0.966 42 | 0.671 703 | 3.034 506 | 3.706 21 | 0 | Huang et al., 2021a |
| Neochlorogenic acid | *Eupatorium lindleyanum* | 4.078 972 521 | −0.026 248 911 | −0.966 423 075 | −4.95 × 10^−17^ | −6.18 × 10^−17^ | −0.966 42 | −0.992 67 | 4.078 973 | 3.086 301 | 0 | Huang et al., 2021a |
| Cryptochlorogenic acid | *Eupatorium lindleyanum* | 4.203 684 926 | −0.135 133 281 | −0.966 423 075 | −4.95 × 10^−17^ | −6.18 × 10^−17^ | −0.966 42 | −1.101 56 | 4.203 685 | 3.102 129 | 0 | Huang et al., 2021a |
| Eupalinolide D | *Eupatorium lindleyanum* | −0.395 085 001 | −0.524 006 031 | −0.966 423 075 | −4.95 × 10^−17^ | −6.18 × 10^−17^ | −0.966 42 | −1.490 43 | −0.395 09 | −1.885 51 | 0 | Huang et al., 2021a |
| Eupalinolide E | *Eupatorium lindleyanum* | −0.395 085 001 | −0.524 006 031 | −0.966 423 075 | −4.95 × 10^−17^ | −6.18 × 10^−17^ | −0.966 42 | −1.490 43 | −0.395 09 | −1.885 51 | 0 | Huang et al., 2021a |
| Eupalinolide J | *Eupatorium lindleyanum* | −0.395 085 001 | −0.524 006 031 | −0.966 423 075 | −4.95 × 10^−17^ | −6.18 × 10^−17^ | −0.966 42 | −1.490 43 | −0.395 09 | −1.885 51 | 0 | Huang et al., 2021a |
| Eupalinolide L | *Eupatorium lindleyanum* | −0.395 085 001 | −0.524 006 031 | −0.966 423 075 | −4.95 × 10^−17^ | −6.18 × 10^−17^ | −0.966 42 | −1.490 43 | −0.395 09 | −1.885 51 | 0 | Huang et al., 2021a |
| Eupalinolide M | *Eupatorium lindleyanum* | −0.395 085 001 | −0.524 006 031 | −0.966 423 075 | −4.95 × 10^−17^ | −6.18 × 10^−17^ | −0.966 42 | −1.490 43 | −0.395 09 | −1.885 51 | 0 | Huang et al., 2021a |
| Eupalinilide A | *Eupatorium lindleyanum* | −0.395 085 001 | −0.524 006 031 | −0.966 423 075 | −4.95 × 10^−17^ | −6.18 × 10^−17^ | −0.966 42 | −1.490 43 | −0.395 09 | −1.885 51 | 0 | Huang et al., 2021a |
| Eupalinilide B | *Eupatorium lindleyanum* | −0.395 085 001 | −0.524 006 031 | −0.966 423 075 | −4.95 × 10^−17^ | −6.18 × 10^−17^ | −0.966 42 | −1.490 43 | −0.395 09 | −1.885 51 | 0 | Huang et al., 2021a |
| Eupalinilide C | *Eupatorium lindleyanum* | −0.395 085 001 | −0.524 006 031 | −0.966 423 075 | −4.95 × 10^−17^ | −6.18 × 10^−17^ | −0.966 42 | −1.490 43 | −0.395 09 | −1.885 51 | 0 | Huang et al., 2021a |
| Eupalinilide D | *Eupatorium lindleyanum* | −0.395 085 001 | −0.524 006 031 | −0.966 423 075 | −4.95 × 10^−17^ | −6.18 × 10^−17^ | −0.966 42 | −1.490 43 | −0.395 09 | −1.885 51 | 0 | Huang et al., 2021a |
| Eupalinilide E | *Eupatorium lindleyanum* | −0.395 085 001 | −0.524 006 031 | −0.966 423 075 | −4.95 × 10^−17^ | −6.18 × 10^−17^ | −0.966 42 | −1.490 43 | −0.395 09 | −1.885 51 | 0 | Huang et al., 2021a |
| Eupalinilide F | *Eupatorium lindleyanum* | −0.395 085 001 | −0.524 006 031 | −0.966 423 075 | −4.95 × 10^−17^ | −6.18 × 10^−17^ | −0.966 42 | −1.490 43 | −0.395 09 | −1.885 51 | 0 | Huang et al., 2021a |
| Eupalinilide G | *Eupatorium lindleyanum* | −0.395 085 001 | −0.524 006 031 | −0.966 423 075 | −4.95 × 10^−17^ | −6.18 × 10^−17^ | −0.966 42 | −1.490 43 | −0.395 09 | −1.885 51 | 0 | Huang et al., 2021a |
| Eupalinilide H | *Eupatorium lindleyanum* | −0.395 085 001 | −0.524 006 031 | −0.966 423 075 | −4.95 × 10^−17^ | −6.18 × 10^−17^ | −0.966 42 | −1.490 43 | −0.395 09 | −1.885 51 | 0 | Huang et al., 2021a |
| Eupalinilide I | *Eupatorium lindleyanum* | −0.395 085 001 | −0.524 006 031 | −0.966 423 075 | −4.95 × 10^−17^ | −6.18 × 10^−17^ | −0.966 42 | −1.490 43 | −0.395 09 | −1.885 51 | 0 | Huang et al., 2021a |
| Eupalinilide J | *Eupatorium lindleyanum* | −0.395 085 001 | −0.524 006 031 | −0.966 423 075 | −4.95 × 10^−17^ | −6.18 × 10^−17^ | −0.966 42 | −1.490 43 | −0.395 09 | −1.885 51 | 0 | Huang et al., 2021a |
| Eupachinilide C | *Eupatorium lindleyanum* | −0.395 085 001 | −0.524 006 031 | −0.966 423 075 | −4.95 × 10^−17^ | −6.18 × 10^−17^ | −0.966 42 | −1.490 43 | −0.395 09 | −1.885 51 | 0 | Huang et al., 2021a |
| Eupachifolin D | *Eupatorium lindleyanum* | −0.395 085 001 | −0.524 006 031 | −0.966 423 075 | −4.95 × 10^−17^ | −6.18 × 10^−17^ | −0.966 42 | −1.490 43 | −0.395 09 | −1.885 51 | 0 | Huang et al., 2021a |
| Eupachinilide E | *Eupatorium lindleyanum* | −0.395 085 001 | −0.524 006 031 | −0.966 423 075 | −4.95 × 10^−17^ | −6.18 × 10^−17^ | −0.966 42 | −1.490 43 | −0.395 09 | −1.885 51 | 0 | Huang et al., 2021a |
| Eupalinin B | *Eupatorium lindleyanum* | −0.395 085 001 | −0.524 006 031 | −0.966 423 075 | −4.95 × 10^−17^ | −6.18 × 10^−17^ | −0.966 42 | −1.490 43 | −0.395 09 | −1.885 51 | 0 | Huang et al., 2021a |
| Eupalinin C | *Eupatorium lindleyanum* | −0.395 085 001 | −0.524 006 031 | −0.966 423 075 | −4.95 × 10^−17^ | −6.18 × 10^−17^ | −0.966 42 | −1.490 43 | −0.395 09 | −1.885 51 | 0 | Huang et al., 2021a |
| Eupalinin D | *Eupatorium lindleyanum* | −0.395 085 001 | −0.524 006 031 | −0.966 423 075 | −4.95 × 10^−17^ | −6.18 × 10^−17^ | −0.966 42 | −1.490 43 | −0.395 09 | −1.885 51 | 0 | Huang et al., 2021a |
| Eupalinin A | *Eupatorium lindleyanum* | −0.395 085 001 | −0.508 451 121 | −0.966 423 075 | −4.95 × 10^−17^ | −6.18 × 10^−17^ | −0.966 42 | −1.474 87 | −0.395 09 | −1.869 96 | 0 | Huang et al., 2021a |
| Taraxasterol palmitate | *Eupatorium lindleyanum* | −0.395 085 001 | −0.492 896 211 | −0.966 423 075 | −4.95 × 10^−17^ | −6.18 × 10^−17^ | −0.966 42 | −1.459 32 | −0.395 09 | −1.854 4 | 0 | Huang et al., 2021a |
| Butyricacid | *Eupatorium lindleyanum* | −0.395 085 001 | −0.150 688 191 | −0.966 423 075 | −4.95 × 10^−17^ | −6.18 × 10^−17^ | −0.966 42 | −1.117 11 | −0.395 09 | −1.512 2 | 0 | Huang et al., 2021a |
| 3,3'-Di-*O*-methylellagicacid | *Euphorbia Pekinensis Radix* | 2.009 842 506 | 0.059 490 688 | 2.444 160 261 | −4.95 × 10^−17^ | −6.18 × 10^−17^ | 2.444 16 | 2.503 651 | 2.009 843 | 4.513 493 | 1 | Zeng et al., 2022 |
| Euphol | *Euphorbia Pekinensis Radix* | 1.708 016 795 | 0.171 607 753 | 1.156 501 075 | −4.95 × 10^−17^ | −6.18 × 10^−17^ | 1.156 501 | 1.328 109 | 1.708 017 | 3.036 126 | 1 | Zeng et al., 2022 |
| Tirucallol | *Euphorbia Pekinensis Radix* | 0.443 888 739 | 0.227 666 286 | 0.719 582 338 | −4.95 × 10^−17^ | −6.18 × 10^−17^ | 0.719 582 | 0.947 249 | 0.443 889 | 1.391 137 | 1 | Zeng et al., 2022 |
| Euphodane B | *Euphorbia Pekinensis Radix* | 0.621 816 859 | 0.507 958 95 | −0.535 356 061 | −4.95 × 10^−17^ | −6.18 × 10^−17^ | −0.535 36 | −0.027 4 | 0.621 817 | 0.594 42 | 1 | Zeng et al., 2022 |
| Jolkinol B | *Euphorbia Pekinensis Radix* | −0.863 091 116 | 0.451 900 417 | 1.345 126 786 | −4.95 × 10^−17^ | −6.18 × 10^−17^ | 1.345 127 | 1.797 027 | −0.863 09 | 0.933 936 | 1 | Zeng et al., 2022 |
| 24-Methylenecycloartenol | *Euphorbia Pekinensis Radix* | 0.400 105 38 | −0.332 919 042 | −0.373 676 881 | −4.95 × 10^−17^ | −6.18 × 10^−17^ | −0.373 68 | −0.706 6 | 0.400 105 | −0.306 49 | 0 | Zeng et al., 2022 |
| Euphodane A | *Euphorbia Pekinensis Radix* | −0.849 117 703 | 0.507 958 95 | 0.356 766 559 | −4.95 × 10^−17^ | −6.18 × 10^−17^ | 0.356 767 | 0.864 726 | −0.849 12 | 0.015 608 | 0 | Zeng et al., 2022 |
| (3*β,*11*α*,13*α*)-3,11-Dihydroxypimara-7,15-dienE2,12-dione | *Euphorbia Pekinensis Radix* | −0.454 135 908 | 0.507 958 95 | −0.811 557 994 | −4.95 × 10^−17^ | −6.18 × 10^−17^ | −0.811 56 | −0.303 6 | −0.454 14 | −0.757 73 | 0 | Zeng et al., 2022 |
| Helioscopinolide B | *Euphorbia Pekinensis Radix* | −0.864 022 676 | 0.339 783 352 | −0.534 393 685 | −4.95 × 10^−17^ | −6.18 × 10^−17^ | −0.534 39 | −0.194 61 | −0.864 02 | −1.058 63 | 0 | Zeng et al., 2022 |
| Guajavarin | *Euphorbia Pekinensis Radix* | −0.684 231 435 | 0.171 607 753 | −0.811 557 994 | −4.95 × 10^−17^ | −6.18 × 10^−17^ | −0.811 56 | −0.639 95 | −0.684 23 | −1.324 18 | 0 | Zeng et al., 2022 |
| Helioscopinolide A | *Euphorbia Pekinensis Radix* | −0.859 364 872 | 0.227 666 286 | −0.660 464 951 | −4.95 × 10^−17^ | −6.18 × 10^−17^ | −0.660 46 | −0.432 8 | −0.859 36 | −1.292 16 | 0 | Zeng et al., 2022 |
| Helioscopinolide E | *Euphorbia Pekinensis Radix* | −0.856 570 19 | 0.283 724 819 | −0.672 013 464 | −4.95 × 10^−17^ | −6.18 × 10^−17^ | −0.672 01 | −0.388 29 | −0.856 57 | −1.244 86 | 0 | Zeng et al., 2022 |
| Helioscopinolide C | *Euphorbia Pekinensis Radix* | −0.853 775 507 | 0.395 841 885 | −0.811 557 994 | −4.95 × 10^−17^ | −6.18 × 10^−17^ | −0.811 56 | −0.415 72 | −0.853 78 | −1.269 49 | 0 | Zeng et al., 2022 |
| Ellagicacid | *Euphorbia Pekinensis Radix* | 1.100 639 129 | −3.520 247 049 | −0.811 557 994 | −4.95 × 10^−17^ | −6.18 × 10^−17^ | −0.811 56 | −4.331 81 | 1.100 639 | −3.231 17 | 0 | Zeng et al., 2022 |
| PO | HDT | 1.031 632 391 | −8.84 × 10^18^ | −0.631 115 968 | 0.302 904 367 | −6.18 × 10^−17^ | −0.328 21 | −0.631 12 | 1.334 537 | 0.703 421 | 0 | Liu et al., 2022 |
| IPO | HDT | 0.607 226 188 | −8.84 × 10^18^ | −0.631 115 968 | 0.395 823 089 | −6.18 × 10^−17^ | −0.235 29 | −0.631 12 | 1.003 049 | 0.371 933 | 0 | Liu et al., 2022 |
| Hyp | HDT | 1.631 976 868 | −8.84 × 10^18^ | 1.358 665 365 | 0.412 312 014 | −6.18 × 10^−17^ | 1.770 977 | 1.358 665 | 2.044 289 | 3.402 954 | 1 | Liu et al., 2022 |
| Qug | HDT | 2.227 932 861 | −8.84 × 10^18^ | 1.210 504 763 | 0.432 326 872 | −6.18 × 10^−17^ | 1.642 832 | 1.210 505 | 2.660 26 | 3.870 764 | 1 | Liu et al., 2022 |
| IQ | HDT | 0.780 531 372 | −8.84 × 10^18^ | 1.643 500 988 | 0.422 060 183 | −6.18 × 10^−17^ | 2.065 561 | 1.643 501 | 1.202 592 | 2.846 093 | 1 | Liu et al., 2022 |
| SeB | HDT | −0.811 370 895 | −8.84 × 10^18^ | −0.631 115 968 | −2.414 242 322 | −6.18 × 10^−17^ | −3.045 36 | −0.631 12 | −3.225 61 | −3.856 73 | 0 | Liu et al., 2022 |
| Ast | HDT | −0.262 938 183 | −8.84 × 10^18^ | −0.631 115 968 | 0.421 645 367 | −6.18 × 10^−17^ | −0.209 47 | −0.631 12 | 0.158 707 | −0.472 41 | 0 | Liu et al., 2022 |
| SeA | HDT | −0.811 370 895 | −8.84 × 10^18^ | −0.631 115 968 | −2.414 242 322 | −6.18 × 10^−17^ | −3.045 36 | −0.631 12 | −3.225 61 | −3.856 73 | 0 | Liu et al., 2022 |
| Roa | HDT | −0.596 383 101 | −8.84 × 10^18^ | 2.021 777 223 | 0.390 430 484 | −6.18 × 10^−17^ | 2.412 208 | 2.021 777 | −0.205 95 | 1.815 825 | 0 | Liu et al., 2022 |
| Lia | HDT | −0.532 367 103 | −8.84 × 10^18^ | 1.370 607 689 | 0.566 312 351 | −6.18 × 10^−17^ | 1.936 92 | 1.370 608 | 0.033 945 | 1.404 553 | 0 | Liu et al., 2022 |
| SaB | HDT | 2.273 573 085 | −8.84 × 10^18^ | −0.631 115 968 | 0.382 237 874 | −6.18 × 10^−17^ | −0.248 88 | −0.631 12 | 2.655 811 | 2.024 695 | 1 | Liu et al., 2022 |
| Nuc | HDT | 0.360 513 652 | −8.84 × 10^18^ | −0.631 115 968 | 0.578 964 23 | −6.18 × 10^−17^ | −0.052 15 | −0.631 12 | 0.939 478 | 0.308 362 | 0 | Liu et al., 2022 |
| SaA | HDT | −0.625 642 315 | −8.84 × 10^18^ | 1.774 596 327 | 0.315 971 062 | −6.18 × 10^−17^ | 2.090 567 | 1.774 596 | −0.309 67 | 1.464 925 | 0 | Liu et al., 2022 |
| P | HDT | −0.431 240 496 | −8.84 × 10^18^ | −0.631 115 968 | 0.679 971 859 | −6.18 × 10^−17^ | 0.048 856 | −0.631 12 | 0.248 731 | −0.382 38 | 0 | Liu et al., 2022 |
| IP | HDT | −0.507 209 126 | −8.84 × 10^18^ | −0.631 115 968 | 0.539 867 848 | −6.18 × 10^−17^ | −0.091 25 | −0.631 12 | 0.032 659 | −0.598 46 | 0 | Liu et al., 2022 |
| SaC | HDT | −0.811 370 895 | −8.84 × 10^18^ | −0.631 115 968 | −2.414 242 322 | −6.18 × 10^−17^ | −3.045 36 | −0.631 12 | −3.225 61 | −3.856 73 | 0 | Liu et al., 2022 |
| Rhe | HDT | −0.737 510 332 | −8.84 × 10^18^ | −0.631 115 968 | 0.632 890 274 | −6.18 × 10^−17^ | 0.001 774 | −0.631 12 | −0.104 62 | −0.735 74 | 0 | Liu et al., 2022 |
| Neo | HDT | −0.741 870 888 | −8.84 × 10^18^ | −0.544 028 81 | 0.381 926 762 | −6.18 × 10^−17^ | −0.162 1 | −0.544 03 | −0.359 94 | −0.903 97 | 0 | Liu et al., 2022 |
| Cor | HDT | −0.799 442 2 | −8.84 × 10^18^ | −0.631 115 968 | 0.234 252 365 | −6.18 × 10^−17^ | −0.396 86 | −0.631 12 | −0.565 19 | −1.196 31 | 0 | Liu et al., 2022 |
| Cry | HDT | −0.568 528 203 | −8.84 × 10^18^ | −0.631 115 968 | 0.333 289 618 | −6.18 × 10^−17^ | −0.297 83 | −0.631 12 | −0.235 24 | −0.866 35 | 0 | Liu et al., 2022 |
| Bak | HDT | −0.676 141 785 | −8.84 × 10^18^ | −0.631 115 968 | −0.180 459 654 | −6.18 × 10^−17^ | −0.811 58 | −0.631 12 | −0.856 6 | −1.487 72 | 0 | Liu et al., 2022 |
| Baicalin | JZol | 4.112 273 631 | −8.84 × 10^18^ | 0.825 580 691 | 3.975 235 05 | 1.253 566 341 | 4.800 816 | 0.825 581 | 8.087 509 | 10.166 66 | 1 | Liu et al., 2023 |
| ChrysinglucuronideB | JZol | −0.453 414 886 | −8.84 × 10^18^ | 0.963 177 472 | 0.000 000 568 | 1.253 566 341 | 0.963 178 | 0.963 177 | −0.453 41 | 1.763 329 | 1 | Liu et al., 2023 |
| OroxylinAglucuronide | JZol | −0.087 467 574 | −8.84 × 10^18^ | 0.412 790 345 | 0.600 288 619 | 1.253 566 341 | 1.013 079 | 0.412 79 | 0.512 821 | 2.179 178 | 1 | Liu et al., 2023 |
| Wogonoside | JZol | 0.495 685 952 | −8.84 × 10^18^ | −1.375 967 818 | 0.902 253 366 | 1.253 566 341 | −0.473 71 | −1.375 97 | 1.397 939 | 1.275 538 | 1 | Liu et al., 2023 |
| Wogonin | JZol | −0.488 910 638 | −8.84 × 10^18^ | 0.275 193 564 | −0.878 478 693 | 1.253 566 341 | −0.603 29 | 0.275 194 | −1.367 39 | 0.161 371 | 0 | Liu et al., 2023 |
| Peimisine | JZol | −0.488 910 638 | −8.84 × 10^18^ | −1.651 161 381 | −0.897 085 881 | 1.253 566 341 | −2.548 25 | −1.651 16 | −1.386 | −1.783 59 | 0 | Liu et al., 2023 |
| Skullcapflavone II | JZol | −0.488 910 638 | −8.84 × 10^18^ | 0.137 596 782 | 0.000 000 568 | 1.253 566 341 | 0.137 597 | 0.137 597 | −0.488 91 | 0.902 253 | 1 | Liu et al., 2023 |
| Gallicacid | JZol | 0.036 388 601 | −8.84 × 10^18^ | −0.963 177 472 | 0.000 000 568 | 0 | −0.963 18 | −0.963 18 | 0.036 389 | −0.926 79 | 0 | Liu et al., 2023 |
| AloEemodin glucopyranoside P6 | JZol | −0.360 633 284 | −8.84 × 10^18^ | 0.412 790 345 | 0.000 000 568 | 0 | 0.412 791 | 0.412 79 | −0.360 63 | 0.052 158 | 1 | Liu et al., 2023 |
| Chrysophanol glucopyranoside | JZol | −0.488 910 638 | −8.84 × 10^18^ | 0.412 790 345 | 0.000 000 568 | 0 | 0.412 791 | 0.412 79 | −0.488 91 | −0.076 12 | 1 | Liu et al., 2023 |
| Chrysophanol glucopyranoside U | JZol | −0.438 256 557 | −8.84 × 10^18^ | 0.687 983 909 | 0.000 000 568 | 0 | 0.687 984 | 0.687 984 | −0.438 26 | 0.249 728 | 0 | Liu et al., 2023 |
| Hyodeoxycholic acid | JZol | 0.714 786 957 | −8.84 × 10^18^ | −0.963 177 472 | 0.000 000 568 | 0 | −0.963 18 | −0.963 18 | 0.714 788 | −0.248 39 | 0 | Liu et al., 2023 |
| Cholicacid | JZol | 0.120 138 365 | −8.84 × 10^18^ | −0.687 983 909 | 0.000 000 568 | 0 | −0.687 98 | −0.687 98 | 0.120 139 | −0.567 84 | 0 | Liu et al., 2023 |
| Rhein | JZol | −0.488 910 638 | −8.84 × 10^18^ | 0.825 580 691 | −0.867 054 396 | 0 | −0.041 47 | 0.825 581 | −1.355 97 | −0.530 38 | 0 | Liu et al., 2023 |
| Deoxycholicacid | JZol | −0.488 910 638 | −8.84 × 10^18^ | −0.963 177 472 | 0.000 000 568 | 0 | −0.963 18 | −0.963 18 | −0.488 91 | −1.452 09 | 0 | Liu et al., 2023 |
| Liquiritin | JZol | 0.044 346 723 | −8.84 × 10^18^ | 1.375 967 818 | −0.391 616 994 | −1.253 566 341 | 0.984 351 | 1.375 968 | −0.347 27 | −0.224 87 | 0 | Liu et al., 2023 |
| Liquiritigenin | JZol | −0.402 508 166 | −8.84 × 10^18^ | 1.238 371 036 | −0.903 514 652 | −1.253 566 341 | 0.334 856 | 1.238 371 | −1.306 02 | −1.321 22 | 0 | Liu et al., 2023 |
| Glycyrrhizicacid | JZol | 1.107 766 621 | −8.84 × 10^18^ | −1.651 161 381 | 0.275 983 581 | −1.253 566 341 | −1.375 18 | −1.651 16 | 1.383 75 | −1.520 98 | 0 | Liu et al., 2023 |
| Isoliquiritin | JZol | −0.488 910 638 | −8.84 × 10^18^ | 0.687 983 909 | −0.908 493 512 | −1.253 566 341 | −0.220 51 | 0.687 984 | −1.397 4 | −1.962 99 | 0 | Liu et al., 2023 |
| Isoliquiritigenin | JZol | −0.488 910 638 | −8.84 × 10^18^ | 0.687 983 909 | −0.907 522 738 | −1.253 566 341 | −0.219 54 | 0.687 984 | −1.396 43 | −1.962 02 | 0 | Liu et al., 2023 |
| Liquiritinapioside | JZol | −0.488 910 638 | −8.84 × 10^18^ | 0.963 177 472 | 0.000 000 568 | −1.253 566 341 | 0.963 178 | 0.963 177 | −0.488 91 | −0.779 3 | 0 | Liu et al., 2023 |
| LicoricEsaponin G2 | JZol | −0.488 910 638 | −8.84 × 10^18^ | −1.651 161 381 | 0.000 000 568 | −1.253 566 341 | −1.651 16 | −1.651 16 | −0.488 91 | −3.393 64 | 0 | Liu et al., 2023 |
| Eleutheroside A | LBZ | −0.517 904 859 | −0.629 649 775 | −1.300 621 558 | −4.95 × 10^−17^ | −6.18 × 10^−17^ | −1.300 62 | −1.930 27 | −0.517 9 | −2.448 18 | 0 | Huang et al., 2021c |
| Methylellagic acid | LBZ | −0.517 904 859 | −0.568 032 606 | −0.215 653 007 | −4.95 × 10^−17^ | −6.18 × 10^−17^ | −0.215 65 | −0.783 69 | −0.517 9 | −1.301 59 | 0 | Huang et al., 2021c |
| Gallic acid | LBZ | 8.42 × 10^17^ | −0.581 707 005 | 0.841 209 661 | −4.95 × 10^−17^ | −6.18 × 10^−17^ | 0.841 21 | 0.259 503 | 3.47 × 10^17^ | 0.259 503 | 1 | Huang et al., 2021c |
| Ellagic acid | LBZ | 2.988 649 497 | −0.569 667 59 | 0.920 069 066 | −4.95 × 10^−17^ | −6.18 × 10^−17^ | 0.920 069 | 0.350 401 | 2.988 649 | 3.339 051 | 1 | Huang et al., 2021c |
| Nigaichigoside F1 | LBZ | −0.517 904 859 | −0.382 902 288 | −1.300 621 558 | −4.95 × 10^−17^ | −6.18 × 10^−17^ | −1.300 62 | −1.683 52 | −0.517 9 | −2.201 43 | 0 | Huang et al., 2021c |
| Isoquercitrin | LBZ | −0.517 904 859 | −0.604 782 553 | 0.033 796 888 | −4.95 × 10^−17^ | −6.18 × 10^−17^ | 0.033 797 | −0.570 99 | −0.517 9 | −1.088 89 | 0 | Huang et al., 2021c |
| Caffeic acid | LBZ | −0.517 904 859 | −0.401 497 969 | 0.432 981 893 | −4.95 × 10^−17^ | −6.18 × 10^−17^ | 0.432 982 | 0.031 484 | −0.517 9 | −0.486 42 | 0 | Huang et al., 2021c |
| Astragalin | LBZ | −0.517 904 859 | −0.595 574 | −1.071 864 11 | −4.95 × 10^−17^ | −6.18 × 10^−17^ | −1.071 86 | −1.667 44 | −0.517 9 | −2.185 34 | 0 | Huang et al., 2021c |
| Kaempferol | LBZ | −0.517 904 859 | −0.617 984 978 | 0.490 334 188 | −4.95 × 10^−17^ | −6.18 × 10^−17^ | 0.490 334 | −0.127 65 | −0.517 9 | −0.645 56 | 0 | Huang et al., 2021c |
| Gemin A | LBZ | −0.517 904 859 | 1.048 965 012 | −1.243 595 129 | −4.95 × 10^−17^ | −6.18 × 10^−17^ | −1.243 6 | −0.194 63 | −0.517 9 | −0.712 53 | 0 | Huang et al., 2021c |
| Casuarinin | LBZ | 0.974 504 088 | −0.335 567 831 | 0.884 142 416 | −4.95 × 10^−17^ | −6.18 × 10^−17^ | 0.884 142 | 0.548 575 | 0.974 504 | 1.523 079 | 0 | Huang et al., 2021c |
| Tellimagrandin II | LBZ | 0.028 098 414 | −0.335 567 831 | 0.943 612 835 | −4.95 × 10^−17^ | −6.18 × 10^−17^ | 0.943 613 | 0.608 045 | 0.028 098 | 0.636 143 | 0 | Huang et al., 2021c |
| Gemin D | LBZ | −0.517 904 859 | −0.220 190 095 | −0.803 676 96 | −4.95 × 10^−17^ | −6.18 × 10^−17^ | −0.803 68 | −1.023 87 | −0.517 9 | −1.541 77 | 0 | Huang et al., 2021c |
| Casuarictin | LBZ | 8.42 × 10^17^ | −0.335 567 831 | 0.924 712 647 | −4.95 × 10^−17^ | −6.18 × 10^−17^ | 0.924 713 | 0.589 145 | 3.47 × 10^17^ | 0.589 145 | 0 | Huang et al., 2021c |
| Gemin G | LBZ | 2.721 714 564 | 2.741 171 822 | 1.284 467 949 | −4.95 × 10^−17^ | −6.18 × 10^−17^ | 1.284 468 | 4.025 64 | 2.721 715 | 6.747 354 | 1 | Huang et al., 2021c |
| Gemin C | LBZ | 0.343 566 972 | 2.741 171 822 | 1.191 270 471 | −4.95 × 10^−17^ | −6.18 × 10^−17^ | 1.191 27 | 3.932 442 | 0.343 567 | 4.276 009 | 1 | Huang et al., 2021c |
| Protocatechuic acid | LBZ | −0.517 904 859 | −0.601 458 913 | −1.251 741 762 | −4.95 × 10^−17^ | −6.18 × 10^−17^ | −1.251 74 | −1.853 2 | −0.517 9 | −2.371 11 | 0 | Huang et al., 2021c |
| Tetra-*O*-galloyl-*β*-*D*-glucose | LBZ | −0.517 904 859 | −0.335 567 831 | −1.333 208 089 | −4.95 × 10^−17^ | −6.18 × 10^−17^ | −1.333 21 | −1.668 78 | −0.517 9 | −2.186 68 | 0 | Huang et al., 2021c |
| Pedunculagin | LBZ | −0.517 904 859 | −0.304 800 435 | 0.927 563 968 | −4.95 × 10^−17^ | −6.18 × 10^−17^ | 0.927 564 | 0.622 764 | −0.517 9 | 0.104 859 | 0 | Huang et al., 2021c |
| Hydroxytormentic acid | LBZ | −0.517 904 859 | −0.159 754 137 | −1.268 035 027 | −4.95 × 10^−17^ | −6.18 × 10^−17^ | −1.268 04 | −1.427 79 | −0.517 9 | −1.945 69 | 0 | Huang et al., 2021c |
| Potentillin | LBZ | −0.323 770 362 | 1.048 965 012 | 0.914 855 221 | −4.95 × 10^−17^ | −6.18 × 10^−17^ | 0.914 855 | 1.963 82 | −0.323 77 | 1.640 05 | 0 | Huang et al., 2021c |
| Quercetin | PPH | 0.886 007 96 | −0.517 140 584 | 2.502 181 968 | −4.95 × 10^−17^ | −6.18 × 10^−17^ | 2.502 182 | 1.985 041 | 0.886 008 | 2.871 049 | 1 | Huang et al., 2021b |
| Oleanolic acid | PPH | −0.071 260 98 | −0.496 411 491 | 1.600 833 116 | −4.95 × 10^−17^ | −6.18 × 10^−17^ | 1.600 833 | 1.104 422 | −0.071 26 | 1.033 161 | 1 | Huang et al., 2021b |
| Isoquercitrin | PPH | −0.305 449 989 | −0.493 783 548 | 1.525 720 712 | −4.95 × 10^−17^ | −6.18 × 10^−17^ | 1.525 721 | 1.031 937 | −0.305 45 | 0.726 487 | 1 | Huang et al., 2021b |
| Ellagic acid | PPH | 0.349 253 59 | −0.461 219 913 | 1.375 495 904 | −4.95 × 10^−17^ | −6.18 × 10^−17^ | 1.375 496 | 0.914 276 | 0.349 254 | 1.263 53 | 1 | Huang et al., 2021b |
| Gallic acid | PPH | −0.079 808 024 | −0.472 384 588 | 1.150 158 691 | −4.95 × 10^−17^ | −6.18 × 10^−17^ | 1.150 159 | 0.677 774 | −0.079 81 | 0.597 966 | 1 | Huang et al., 2021b |
| Catechin | PPH | −0.305 449 989 | −0.454 721 953 | 1.075 046 286 | −4.95 × 10^−17^ | −6.18 × 10^−17^ | 1.075 046 | 0.620 324 | −0.305 45 | 0.314 874 | 0 | Huang et al., 2021b |
| Kaempferol | PPH | −0.219 979 548 | −0.506 026 735 | 0.849 709 074 | −4.95 × 10^−17^ | −6.18 × 10^−17^ | 0.849 709 | 0.343 682 | −0.219 98 | 0.123 703 | 0 | Huang et al., 2021b |
| Rutin | PPH | 0.087 714 04 | −0.494 184 278 | 0.812 152 871 | −4.95 × 10^−17^ | −6.18 × 10^−17^ | 0.812 153 | 0.317 969 | 0.087 714 | 0.405 683 | 0 | Huang et al., 2021b |
| Ursolic acid | PPH | 0.209 082 066 | −0.491 173 919 | 0.624 371 861 | −4.95 × 10^−17^ | −6.18 × 10^−17^ | 0.624 372 | 0.133 198 | 0.209 082 | 0.342 28 | 0 | Huang et al., 2021b |
| Aesculetin | PPH | −0.300 321 762 | −0.450 985 627 | 0.624 371 861 | −4.95 × 10^−17^ | −6.18 × 10^−17^ | 0.624 372 | 0.173 386 | −0.300 32 | −0.126 94 | 0 | Huang et al., 2021b |
| AloEemodin | PPH | −0.305 449 989 | −0.364 263 523 | 0.399 034 648 | −4.95 × 10^−17^ | −6.18 × 10^−17^ | 0.399 035 | 0.034 771 | −0.305 45 | −0.270 68 | 0 | Huang et al., 2021b |
| Emodin | PPH | −0.305 449 989 | −0.428 206 088 | 0.399 034 648 | −4.95 × 10^−17^ | −6.18 × 10^−17^ | 0.399 035 | −0.029 17 | −0.305 45 | −0.334 62 | 0 | Huang et al., 2021b |
| Isorhamnetin | PPH | −0.305 449 989 | −0.475 336 168 | 0.511 703 254 | −4.95 × 10^−17^ | −6.18 × 10^−17^ | 0.511 703 | 0.036 367 | −0.305 45 | −0.269 08 | 0 | Huang et al., 2021b |
| Protocatechuicacid | PPH | −0.025 106 942 | −0.490 701 394 | 0.323 922 244 | −4.95 × 10^−17^ | −6.18 × 10^−17^ | 0.323 922 | −0.166 78 | −0.025 11 | −0.191 89 | 0 | Huang et al., 2021b |
| Ferulicacid | PPH | −0.088 355 068 | −0.504 340 369 | 0.323 922 244 | −4.95 × 10^−17^ | −6.18 × 10^−17^ | 0.323 922 | −0.180 42 | −0.088 36 | −0.268 77 | 0 | Huang et al., 2021b |
| *β*-Sitosterol | PPH | −0.305 449 989 | −0.516 690 295 | −0.051 639 778 | −4.95 × 10^−17^ | −6.18 × 10^−17^ | −0.051 64 | −0.568 33 | −0.305 45 | −0.873 78 | 0 | Huang et al., 2021b |
| Dihydroquercetin | PPH | −0.305 449 989 | −0.372 522 771 | −0.164 308 384 | −4.95 × 10^−17^ | −6.18 × 10^−17^ | −0.164 31 | −0.536 83 | −0.305 45 | −0.842 28 | 0 | Huang et al., 2021b |
| Hyperoside | PPH | −0.305 449 989 | −0.492 085 218 | −0.389 645 597 | −4.95 × 10^−17^ | −6.18 × 10^−17^ | −0.389 65 | −0.881 73 | −0.305 45 | −1.187 18 | 0 | Huang et al., 2021b |
| Cucurbitacin IIA | PPH | −0.305 449 989 | 2.609 065 754 | −0.727 651 417 | −4.95 × 10^−17^ | −6.18 × 10^−17^ | −0.727 65 | 1.881 414 | −0.305 45 | 1.575 964 | 0 | Huang et al., 2021b |
| Protocatechualdehyde | PPH | −0.226 817 183 | −0.333 291 343 | −0.765 207 619 | −4.95 × 10^−17^ | −6.18 × 10^−17^ | −0.765 21 | −1.098 5 | −0.226 82 | −1.325 32 | 0 | Huang et al., 2021b |
| Quercetin-3-*O*-glucuronide | PPH | 5.390 300 204 | −0.180 724 679 | −0.802 763 821 | −4.95 × 10^−17^ | −6.18 × 10^−17^ | −0.802 76 | −0.983 49 | 5.390 3 | 4.406 812 | 0 | Huang et al., 2021b |
| Physcion | PPH | −0.305 449 989 | −0.428 206 088 | −0.840 320 023 | −4.95 × 10^−17^ | −6.18 × 10^−17^ | −0.840 32 | −1.268 53 | −0.305 45 | −1.573 98 | 0 | Huang et al., 2021b |
| 3′,4′,5′,5,7-Pentamethoxyflavone | PPH | −0.305 449 989 | 2.609 065 754 | −0.877 876 225 | −4.95 × 10^−17^ | −6.18 × 10^−17^ | −0.877 88 | 1.731 19 | −0.305 45 | 1.425 74 | 0 | Huang et al., 2021b |
| 5-Hydroxymethylfurfural | PPH | −0.283 227 674 | −0.452 899 355 | −0.877 876 225 | −4.95 × 10^−17^ | −6.18 × 10^−17^ | −0.877 88 | −1.330 78 | −0.283 23 | −1.614 | 0 | Huang et al., 2021b |
| *α*-Tocopherylquinone | PPH | −0.305 449 989 | 2.609 065 754 | −0.915 432 427 | −4.95 × 10^−17^ | −6.18 × 10^−17^ | −0.915 43 | 1.693 633 | −0.305 45 | 1.388 183 | 0 | Huang et al., 2021b |
| 3,3′-Dimethoxyellagicacid | PPH | −0.129 380 88 | 0.098 254 364 | −0.952 988 629 | −4.95 × 10^−17^ | −6.18 × 10^−17^ | −0.952 99 | −0.854 73 | −0.129 38 | −0.984 12 | 0 | Huang et al., 2021b |
| Avicularin | PPH | −0.305 449 989 | −0.081 089 306 | −0.990 544 832 | −4.95 × 10^−17^ | −6.18 × 10^−17^ | −0.990 54 | −1.071 63 | −0.305 45 | −1.377 08 | 0 | Huang et al., 2021b |
| Kaempferol-3-*O*-rutinoside | PPH | −0.305 449 989 | −0.305 268 895 | −1.028 101 034 | −4.95 × 10^−17^ | −6.18 × 10^−17^ | −1.028 1 | −1.333 37 | −0.305 45 | −1.638 82 | 0 | Huang et al., 2021b |
| Pinocembrin | PPH | −0.305 449 989 | −0.288 024 311 | −1.178 325 842 | −4.95 × 10^−17^ | −6.18 × 10^−17^ | −1.178 33 | −1.466 35 | −0.305 45 | −1.771 8 | 0 | Huang et al., 2021b |
| Methylcoumarate | PPH | −0.305 449 989 | −0.081 089 306 | −1.178 325 842 | −4.95 × 10^−17^ | −6.18 × 10^−17^ | −1.178 33 | −1.259 42 | −0.305 45 | −1.564 87 | 0 | Huang et al., 2021b |
| Skimmin | PPH | −0.305 449 989 | 0.098 254 364 | −1.178 325 842 | −4.95 × 10^−17^ | −6.18 × 10^−17^ | −1.178 33 | −1.080 07 | −0.305 45 | −1.385 52 | 0 | Huang et al., 2021b |
| Methylferulate | PPH | −0.305 449 989 | 2.609 065 754 | −1.178 325 842 | −4.95 × 10^−17^ | −6.18 × 10^−17^ | −1.178 33 | 1.430 74 | −0.305 45 | 1.125 29 | 0 | Huang et al., 2021b |
| Alisol A | QLQX | −0.010 929 093 | −8.84 × 10^18^ | 2.143 530 293 | −0.119 646 363 | −1.488 416 815 | 2.023 884 | 2.143 53 | −0.130 58 | 0.524 538 | 1 | He et al., 2021 |
| Astragaloside | QLQX | 0.100 952 59 | −8.84 × 10^18^ | 0.560 465 033 | 0.129 812 203 | 0.868 243 142 | 0.690 277 | 0.560 465 | 0.230 765 | 1.659 473 | 1 | He et al., 2021 |
| Benzoylaconine | QLQX | −0.437 540 302 | −8.84 × 10^18^ | −0.164 598 412 | −0.441 829 951 | 0.868 243 142 | −0.606 43 | −0.164 6 | −0.879 37 | −0.175 73 | 0 | He et al., 2021 |
| Benzoylhypaconine | QLQX | −0.432 541 998 | −8.84 × 10^18^ | 0.061 963 774 | −0.685 973 426 | 0.868 243 142 | −0.624 01 | 0.061 964 | −1.118 52 | −0.188 31 | 0 | He et al., 2021 |
| Benzoylmesaconine | QLQX | −0.381 071 366 | −8.84 × 10^18^ | 0.061 963 774 | −0.694 836 408 | 0.868 243 142 | −0.632 87 | 0.061 964 | −1.075 91 | −0.145 7 | 0 | He et al., 2021 |
| Calycosin-7-*O*-*β*-*D*-glucopyranoside | QLQX | −0.160 045 166 | −8.84 × 10^18^ | −0.422 136 92 | 1.258 918 438 | 0.868 243 142 | 0.836 782 | −0.422 14 | 1.098 873 | 1.544 979 | 1 | He et al., 2021 |
| Formononetin | QLQX | −0.305 516 644 | −8.84 × 10^18^ | −0.635 879 582 | 2.862 484 289 | 0.868 243 142 | 2.226 605 | −0.635 88 | 2.556 968 | 2.789 331 | 0 | He et al., 2021 |
| Fuziline | QLQX | −0.440 307 221 | −8.84 × 10^18^ | −0.515 035 674 | −0.059 939 267 | 0.868 243 142 | −0.574 97 | −0.515 04 | −0.500 25 | −0.147 04 | 0 | He et al., 2021 |
| Ginsenoside Rb_1_ | QLQX | 0.097 218 738 | −8.84 × 10^18^ | −0.300 567 949 | −0.831 396 492 | −0.310 086 836 | −1.131 96 | −0.300 57 | −0.734 18 | −1.344 83 | 0 | He et al., 2021 |
| Ginsenoside Re | QLQX | 0.900 949 021 | −8.84 × 10^18^ | −0.784 668 643 | −0.947 856 355 | −0.310 086 836 | −1.732 52 | −0.784 67 | −0.046 91 | −1.141 66 | 1 | He et al., 2021 |
| Hesperidin | QLQX | −0.133 238 785 | −8.84 × 10^18^ | 0.786 906 375 | −0.412 273 185 | −1.488 416 815 | 0.374 633 | 0.786 906 | −0.545 51 | −1.247 02 | 1 | He et al., 2021 |
| Neoline | QLQX | −0.437 599 806 | −8.84 × 10^18^ | −0.635 879 582 | −0.641 051 458 | 0.868 243 142 | −1.276 93 | −0.635 88 | −1.078 65 | −0.846 29 | 0 | He et al., 2021 |
| Periplocin | QLQX | −0.299 744 792 | −8.84 × 10^18^ | −0.542 980 828 | −0.477 295 373 | −1.488 416 815 | −1.020 28 | −0.542 98 | −0.777 04 | −2.808 44 | 0 | He et al., 2021 |
| Periplocymarin | QLQX | −0.437 837 82 | −8.84 × 10^18^ | 0.210 027 771 | −0.487 939 046 | −1.488 416 815 | −0.277 91 | 0.210 028 | −0.925 78 | −2.204 17 | 0 | He et al., 2021 |
| Periplogenin | QLQX | −0.363 755 812 | −8.84 × 10^18^ | −2.206 850 381 | 0.963 930 851 | −1.488 416 815 | −1.242 92 | −2.206 85 | 0.600 175 | −3.095 09 | 0 | He et al., 2021 |
| Sinapinethiocyanate | QLQX | 4.020 262 708 | −8.84 × 10^18^ | −0.300 688 793 | −1.128 744 843 | −0.310 086 836 | −1.429 43 | −0.300 69 | 2.891 518 | 2.280 742 | 0 | He et al., 2021 |
| Songorine | QLQX | −0.432 854 392 | −8.84 × 10^18^ | 2.022 686 386 | 0.245 678 503 | 0.868 243 142 | 2.268 365 | 2.022 686 | −0.187 18 | 2.703 754 | 1 | He et al., 2021 |
| Talatisamine | QLQX | −0.442 806 373 | −8.84 × 10^18^ | −0.635 879 582 | −0.281 028 194 | 0.868 243 142 | −0.916 91 | −0.635 88 | −0.723 83 | −0.491 47 | 0 | He et al., 2021 |
| Tanshinone IIA | QLQX | −0.403 593 487 | −8.84 × 10^18^ | 1.297 622 94 | 1.748 986 077 | −0.310 086 836 | 3.046 609 | 1.297 623 | 1.345 393 | 2.332 929 | 1 | He et al., 2021 |
| Hydroxysaffloryellow A | SJHXJN | 1.771 759 136 | 0.852 988 992 | 1.553 329 112 | 1.243 163 121 | 0.792 405 816 | 2.796 492 | 2.406 318 | 3.014 922 | 6.213 646 | 1 | Zhang et al., 2018 |
| Tracheloside | SJHXJN | 1.771 759 136 | 0.852 988 992 | 1.447 231 926 | 1.243 163 121 | −0.440 225 453 | 2.690 395 | 2.300 221 | 3.014 922 | 4.874 918 | 1 | Zhang et al., 2018 |
| Eleutheroside B | SJHXJN | 1.771 759 136 | −1.057 706 351 | 1.513 542 667 | 1.243 163 121 | 0.792 405 816 | 2.756 706 | 0.455 836 | 3.014 922 | 4.263 164 | 1 | Zhang et al., 2018 |
| Formononetin | SJHXJN | −0.839 254 327 | 0.852 988 992 | 1.447 231 926 | 1.243 163 121 | −0.440 225 453 | 2.690 395 | 2.300 221 | 0.403 909 | 2.263 904 | 0 | Zhang et al., 2018 |
| Rosmarinic acid | SJHXJN | 0.031 083 494 | 0.852 988 992 | −0.528 828 161 | 1.243 163 121 | −1.672 856 722 | 0.714 335 | 0.324 161 | 1.274 247 | −0.074 45 | 0 | Zhang et al., 2018 |
| Kaempferide | SJHXJN | 1.771 759 136 | 0.852 988 992 | −1.032 789 794 | 1.243 163 121 | 0.792 405 816 | 0.210 373 | −0.179 8 | 3.014 922 | 3.627 527 | 0 | Zhang et al., 2018 |
| 4-Methoxysalicylaldehyde | SJHXJN | 1.771 759 136 | 0.852 988 992 | 1.327 872 592 | 1.243 163 121 | −0.440 225 453 | 2.571 036 | 2.180 862 | 3.014 922 | 4.755 558 | 1 | Zhang et al., 2018 |
| Protocatechuic acid | SJHXJN | 1.771 759 136 | 0.852 988 992 | 0.240 376 437 | −0.804 399 667 | 0.792 405 816 | −0.564 02 | 1.093 365 | 0.967 359 | 2.853 131 | 1 | Zhang et al., 2018 |
| Protocatechualdehyde | SJHXJN | 0.031 083 494 | 0.852 988 992 | 0.717 813 773 | 1.243 163 121 | 0.792 405 816 | 1.960 977 | 1.570 803 | 1.274 247 | 3.637 455 | 1 | Zhang et al., 2018 |
| Syringaresinol | SJHXJN | −0.839 254 327 | 0.852 988 992 | 0.638 240 884 | 1.243 163 121 | 0.792 405 816 | 1.881 404 | 1.491 23 | 0.403 909 | 2.687 544 | 0 | Zhang et al., 2018 |
| Homoeriodictyol-7-*O*-*β*-*D*-apiofuranosyl-(1→2)-*β*-*D*-glucopyranoside | SJHXJN | −0.839 254 327 | 0.852 988 992 | 0.001 657 769 | 1.243 163 121 | 0.792 405 816 | 1.244 821 | 0.854 647 | 0.403 909 | 2.050 961 | 0 | Zhang et al., 2018 |
| PeriplosideA | SJHXJN | −0.839 254 327 | 0.852 988 992 | 0.691 289 477 | −0.804 399 667 | −0.440 225 453 | −0.113 11 | 1.544 278 | −1.643 65 | −0.539 6 | 0 | Zhang et al., 2018 |
| Nortracheloside | SJHXJN | −0.839 254 327 | 0.852 988 992 | −0.475 779 568 | −0.804 399 667 | −0.440 225 453 | −1.280 18 | 0.377 209 | −1.643 65 | −1.706 67 | 0 | Zhang et al., 2018 |
| Obscurinine | SJHXJN | −0.839 254 327 | 0.852 988 992 | 1.288 086 147 | −0.804 399 667 | −1.672 856 722 | 0.483 686 | 2.141 075 | −1.643 65 | −1.175 44 | 0 | Zhang et al., 2018 |
| Palmiticacid | SJHXJN | 0.031 083 494 | 0.852 988 992 | 1.327 872 592 | −0.804 399 667 | −0.440 225 453 | 0.523 473 | 2.180 862 | −0.773 32 | 0.967 32 | 0 | Zhang et al., 2018 |
| Luteolin-7-*O*-*β*-*D*-glucoside | SJHXJN | −0.839 254 327 | 0.852 988 992 | 0.028 182 065 | −0.804 399 667 | −1.672 856 722 | −0.776 22 | 0.881 171 | −1.643 65 | −2.435 34 | 0 | Zhang et al., 2018 |
| *α*-Cyperone | SJHXJN | 0.031 083 494 | 0.852 988 992 | 0.571 930 143 | 1.243 163 121 | 0.792 405 816 | 1.815 093 | 1.424 919 | 1.274 247 | 3.491 572 | 0 | Zhang et al., 2018 |
| Stigmasterol | SJHXJN | 0.031 083 494 | −1.057 706 351 | −1.032 789 794 | −0.804 399 667 | 0.792 405 816 | −1.837 19 | −2.090 5 | −0.773 32 | −2.071 41 | 0 | Zhang et al., 2018 |
| *β*-Sitosterol | SJHXJN | 0.031 083 494 | −1.057 706 351 | −1.032 789 794 | −0.804 399 667 | 0.792 405 816 | −1.837 19 | −2.090 5 | −0.773 32 | −2.071 41 | 0 | Zhang et al., 2018 |
| Lignans | SJHXJN | 0.031 083 494 | −1.057 706 351 | −0.926 692 608 | −0.804 399 667 | 0.792 405 816 | −1.731 09 | −1.984 4 | −0.773 32 | −1.965 31 | 0 | Zhang et al., 2018 |
| *β*-Carotene | SJHXJN | 0.031 083 494 | −1.057 706 351 | −1.032 789 794 | −0.804 399 667 | 0.792 405 816 | −1.837 19 | −2.090 5 | −0.773 32 | −2.071 41 | 0 | Zhang et al., 2018 |
| Pyrethrin | SJHXJN | 0.031 083 494 | −1.057 706 351 | −0.820 595 422 | −0.804 399 667 | 0.792 405 816 | −1.625 | −1.878 3 | −0.773 32 | −1.859 21 | 0 | Zhang et al., 2018 |
| Lupeolpalmitate | SJHXJN | −0.839 254 327 | −1.057 706 351 | −0.939 954 756 | −0.804 399 667 | 0.792 405 816 | −1.744 35 | −1.997 66 | −1.643 65 | −2.848 91 | 0 | Zhang et al., 2018 |
| Baicalein | SJHXJN | −0.839 254 327 | −1.057 706 351 | −0.966 479 052 | −0.804 399 667 | 0.792 405 816 | −1.770 88 | −2.024 19 | −1.643 65 | −2.875 43 | 0 | Zhang et al., 2018 |
| Gallicacid | SJHXJN | −0.839 254 327 | −1.057 706 351 | −0.959 847 978 | −0.804 399 667 | 0.792 405 816 | −1.764 25 | −2.017 55 | −1.643 65 | −2.868 8 | 0 | Zhang et al., 2018 |
| Hederagenin | SJHXJN | −0.839 254 327 | −1.057 706 351 | −1.032 789 794 | −0.804 399 667 | −1.672 856 722 | −1.837 19 | −2.090 5 | −1.643 65 | −5.407 01 | 0 | Zhang et al., 2018 |
| Campesterol | SJHXJN | −0.839 254 327 | −2.013 054 022 | −0.999 634 423 | −0.804 399 667 | −1.672 856 722 | −1.804 03 | −3.012 69 | −1.643 65 | −6.329 2 | 0 | Zhang et al., 2018 |
| Licochalcone A | SJHXJN | −0.839 254 327 | −1.057 706 351 | −1.012 896 571 | −0.804 399 667 | −1.672 856 722 | −1.817 3 | −2.070 6 | −1.643 65 | −5.387 11 | 0 | Zhang et al., 2018 |
| Mangiferin | SSPF | −0.314 161 975 | −8.84 × 10^18^ | 1.782 978 729 | 1.019 511 164 | −6.18 × 10^−17^ | 2.802 49 | 1.782 979 | 0.705 349 | 2.488 328 | 1 | Chen et al., 2022 |
| Tanshinone IIA | SSPF | −0.259 787 787 | −8.84 × 10^18^ | 1.241 654 485 | 1.158 449 949 | −6.18 × 10^−17^ | 2.400 104 | 1.241 654 | 0.898 662 | 2.140 317 | 1 | Chen et al., 2022 |
| Ginsenoside Rg_1_ | SSPF | −0.850 136 113 | −8.84 × 10^18^ | 0.665 777 63 | −0.972 241 63 | −6.18 × 10^−17^ | −0.306 46 | 0.665 778 | −1.822 38 | −1.156 6 | 0 | Chen et al., 2022 |
| Salvianolicacid B | SSPF | 1.821 966 837 | −8.84 × 10^18^ | 0.055 348 164 | −0.043 014 287 | −6.18 × 10^−17^ | 0.012 334 | 0.055 348 | 1.778 953 | 1.834 301 | 1 | Chen et al., 2022 |
| Naringin | SSPF | 0.703 412 114 | −8.84 × 10^18^ | 0.046 710 012 | 1.682 439 169 | −6.18 × 10^−17^ | 1.729 149 | 0.046 71 | 2.385 851 | 2.432 561 | 1 | Chen et al., 2022 |
| Liquiritin | SSPF | −0.951 116 748 | −8.84 × 10^18^ | −0.529 166 843 | −0.972 241 63 | −6.18 × 10^−17^ | −1.501 41 | −0.529 17 | −1.923 36 | −2.452 53 | 0 | Chen et al., 2022 |
| Cryptotanshinone | SSPF | −0.881 207 077 | −8.84 × 10^18^ | −1.061 852 934 | −0.972 241 63 | −6.18 × 10^−17^ | −2.034 09 | −1.061 85 | −1.853 45 | −2.915 3 | 0 | Chen et al., 2022 |
| Timosaponin BII | SSPF | 1.449 115 262 | −8.84 × 10^18^ | −1.096 405 545 | −0.972 241 63 | −6.18 × 10^−17^ | −2.068 65 | −1.096 41 | 0.476 874 | −0.619 53 | 0 | Chen et al., 2022 |
| Glycyrrhizic acid | SSPF | −0.718 084 514 | −8.84 × 10^18^ | −1.105 043 698 | 0.071 580 523 | −6.18 × 10^−17^ | −1.033 46 | −1.105 04 | −0.646 5 | −1.751 55 | 1 | Chen et al., 2022 |
| Gastrodin | Tianshu Capsule | 2.201 272 034 | 0.240 603 184 | 1.191 369 152 | 0.570 351 825 | −2 | 1.761 721 | 1.431 972 | 2.771 624 | 2.203 596 | 1 | Gu et al., 2024 |
| Parishin E | Tianshu Capsule | −0.469 547 264 | 0.482 002 188 | −1.135 025 072 | −1.964 545 177 | −2 | −3.099 57 | −0.653 02 | −2.434 09 | −5.087 12 | 0 | Gu et al., 2024 |
| Chlorogenic acid | Tianshu Capsule | −0.263 389 553 | −1.687 936 117 | −1.308 899 381 | −0.063 372 425 | 0.5 | −1.372 27 | −2.996 84 | −0.326 76 | −2.823 6 | 0 | Gu et al., 2024 |
| Ferulic acid | Tianshu Capsule | 0.733 552 213 | −1.929 335 122 | 0.779 793 258 | 0.570 351 825 | 0.5 | 1.350 145 | −1.149 54 | 1.303 904 | 0.654 362 | 0 | Gu et al., 2024 |
| Isochlorogenic acid A | Tianshu Capsule | −1.054 173 607 | −0.722 340 099 | −1.878 942 998 | −1.964 545 177 | 0.5 | −3.843 49 | −2.601 28 | −3.018 72 | −5.12 | 0 | Gu et al., 2024 |
| SenkyunolideI | Tianshu Capsule | 1.115 097 826 | 0.723 401 193 | 0.454 054 048 | 0.570 351 825 | 0.5 | 1.024 406 | 1.177 455 | 1.685 45 | 3.362 905 | 1 | Gu et al., 2024 |
| Senkyunolide H | Tianshu Capsule | −0.848 015 897 | 0.723 401 193 | 0.163 529 887 | 0.570 351 825 | 0.5 | 0.733 882 | 0.886 931 | −0.277 66 | 1.109 267 | 0 | Gu et al., 2024 |
| Senkyunolide A | Tianshu Capsule | 0.087 386 254 | 0.723 401 193 | 0.416 638 057 | 0.570 351 825 | 0.5 | 0.986 99 | 1.140 039 | 0.657 738 | 2.297 777 | 1 | Gu et al., 2024 |
| Z-Ligustilide | Tianshu Capsule | −0.509 548 013 | 0.723 401 193 | 0.370 418 305 | 0.570 351 825 | 0.5 | 0.940 77 | 1.093 819 | 0.060 804 | 1.654 623 | 0 | Gu et al., 2024 |
| Levistilide A | Tianshu Capsule | −0.992 633 992 | 0.723 401 193 | 0.947 064 744 | 0.570 351 825 | 0.5 | 1.517 417 | 1.670 466 | −0.422 28 | 1.748 184 | 0 | Gu et al., 2024 |
| Schisandrol A | WWZ | 3.383 368 77 | 0.392 232 27 | 0.435 182 732 | −4.95 × 10^−17^ | −6.18 × 10^−17^ | 0.435 183 | 0.827 415 | 3.383 369 | 4.210 784 | 1 | Huang et al., 2021d |
| Schisantherin A | WWZ | −0.204 865 583 | 0.392 232 27 | −0.332 786 795 | −4.95 × 10^−17^ | −6.18 × 10^−17^ | −0.332 79 | 0.059 445 | −0.204 87 | −0.145 42 | 0 | Huang et al., 2021d |
| Deoxyharringtonine | WWZ | −0.731 360 43 | 0.392 232 27 | −0.716 771 559 | −4.95 × 10^−17^ | −6.18 × 10^−17^ | −0.716 77 | −0.324 54 | −0.731 36 | −1.055 9 | 0 | Huang et al., 2021d |
| Angeloylgomisin O | WWZ | −0.731 360 43 | −2.549 509 757 | −0.332 786 795 | −4.95 × 10^−17^ | −6.18 × 10^−17^ | −0.332 79 | −2.882 3 | −0.731 36 | −3.613 66 | 0 | Huang et al., 2021d |
| Tigloylgomisin P | WWZ | −0.731 360 43 | 0.392 232 27 | −0.716 771 559 | −4.95 × 10^−17^ | −6.18 × 10^−17^ | −0.716 77 | −0.324 54 | −0.731 36 | −1.055 9 | 0 | Huang et al., 2021d |
| Schisandrol B | WWZ | 0.348 086 292 | 0.392 232 27 | −0.332 786 795 | −4.95 × 10^−17^ | −6.18 × 10^−17^ | −0.332 79 | 0.059 445 | 0.348 086 | 0.407 532 | 0 | Huang et al., 2021d |
| Gomisin G | WWZ | −0.421 813 208 | 0.392 232 27 | −0.332 786 795 | −4.95 × 10^−17^ | −6.18 × 10^−17^ | −0.332 79 | 0.059 445 | −0.421 81 | −0.362 37 | 0 | Huang et al., 2021d |
| Gomisin R | WWZ | −0.731 360 43 | −2.549 509 757 | −0.332 786 795 | −4.95 × 10^−17^ | −6.18 × 10^−17^ | −0.332 79 | −2.882 3 | −0.731 36 | −3.613 66 | 0 | Huang et al., 2021d |
| Schisandrin C | WWZ | −0.187 007 089 | 0.392 232 27 | 0.435 182 732 | −4.95 × 10^−17^ | −6.18 × 10^−17^ | 0.435 183 | 0.827 415 | −0.187 01 | 0.640 408 | 0 | Huang et al., 2021d |
| Schisandrin B | WWZ | 0.685 413 392 | 0.392 232 27 | 2.739 091 315 | −4.95 × 10^−17^ | −6.18 × 10^−17^ | 2.739 091 | 3.131 324 | 0.685 413 | 3.816 737 | 1 | Huang et al., 2021d |
| Schisandrin A | WWZ | 0.253 502 418 | 0.392 232 27 | 1.971 121 787 | −4.95 × 10^−17^ | −6.18 × 10^−17^ | 1.971 122 | 2.363 354 | 0.253 502 | 2.616 856 | 1 | Huang et al., 2021d |
| GomisinD | WWZ | −0.374 190 559 | 0.392 232 27 | −0.716 771 559 | −4.95 × 10^−17^ | −6.18 × 10^−17^ | −0.716 77 | −0.324 54 | −0.374 19 | −0.698 73 | 0 | Huang et al., 2021d |
| Angeloylgomisin H | WWZ | 0.151 642 863 | 0.392 232 27 | −0.716 771 559 | −4.95 × 10^−17^ | −6.18 × 10^−17^ | −0.716 77 | −0.324 54 | 0.151 643 | −0.172 9 | 0 | Huang et al., 2021d |
| Schisantherin B | WWZ | −0.143 352 994 | 0.392 232 27 | −0.332 786 795 | −4.95 × 10^−17^ | −6.18 × 10^−17^ | −0.332 79 | 0.059 445 | −0.143 35 | −0.083 91 | 0 | Huang et al., 2021d |
| Schisanhenol | WWZ | −0.565 342 582 | 0.392 232 27 | −0.716 771 559 | −4.95 × 10^−17^ | −6.18 × 10^−17^ | −0.716 77 | −0.324 54 | −0.565 34 | −0.889 88 | 0 | Huang et al., 2021d |

Notes: PO, psoralenoside; IPO, isopsoralenoside; Hyp, hyperoside; Qug, quercetin-3-*O*-glucuronide; IQ, isoquercitrin; SeB, sennoside B; Ast, astragaline; SeA, sennoside A; Roa, rosmarinic acid; Lia, lithospermic acid; SaB, salvianolic acid B; Nuc, nuciferine; SaA, salvianolic acid A; Ps, psoralen; IP, isopsoralen; SaC, salvianolic acid C; Rhe, rhein; Neo, neobavaisoflavone; Cor, corylin; Cry, cryptotanshinone; Bak, bakuchiol. BZBS, Bazi Bushen Capsule; BZYQT, Buzhong Yiqi Tang; DLP, Danlou Tablet; HDT, Hedan Tablet; JZol, Jin-Zhen oral liquid; LBZ, Lanzhou Buzheng; PPH, Gangban Gui; QLQX, Qiliqiangxin Capsule; SJHXJN, Shujin Huoxue Capsule; SSPF, Shuangshen Pingfei Formula; WWZ, *Schisandra chinensis* (Turcz.) Baill..

**Table S2** Identification of 124 compounds in XYHJ, XYW, and XYKL.

| No. | t*_R_* (min) | Selected ion | Elemental composition | Measured mass | Mass error (× 10^−6^) | (ESI^+^) MS Fragmentation | (ESI^−^) MS Fragmentation | Identification | Class | From | XYHJ | XYW | XYKL |
| --- | --- | --- | --- | --- | --- | --- | --- | --- | --- | --- | --- | --- | --- |
| 1 | 0.42 | [M+H]^+^ | C_12_H_25_N_4_O_7_ | 337.172 8 | 1.5 | 175.120 3, 158.093 6, 116.071 8, 70.066 0 | 335.156 7, 173.104 1 | Arginine + Glc | OA | P/A/B/G | + | + | + |
| 2 | 0.45 | [M−H]^−^ | C_6_H_13_N_4_O_2_ | 173.103 3 | −3.5 | 175.119 5 | 156.076 2,131.082 9 | Arginine | OA | G/P/A/AS/B | + | + | + |
| 3 | 0.45 | [M+H]^+^ | C_12_H_25_N_4_O_7_ | 337.172 7 | 1.2 | 175.120 6,158.093 2,116.071 7,70.065 5 | 335.158 1,173.104 4 | Arginine + Glc | OA | P/A/B/G | + | + | + |
| 4 | 0.46 | [M+H]^+^ | C_16_H_23_O_10_ | 375.128 4 | −1.9 | 357.106 5,213.074 6,195.064 9 | − | Geniposidic acid | OA | B/A/P | + | + | + |
| 5 | 0.46 | [M−H]^−^ | C_4_H_7_N_2_O_3_ | 131.046 1 | 3.1 | 133.061 6 | 114.019 2 | Asparagine | OA | P/A/Z/G/B/ | + | + | + |
| 6 | 0.47 | [M+COOH]^−^ | C_13_H_23_O_13_ | 387.113 8 | −0.3 | − | 341.109 3,195.051 1,179.0565161.0453,143.036 0,119.036 4,113.023 9,101.025 3,89.024 0 | Sucrose | O | P/A/PR/AS/Z/G | + | + | + |
| 7 | 0.49 | [M+H]^+^ | C_5_H_10_NO_2_ | 116.071 7 | 4.3 | 70.067 2 | − | Proline | OA | G/A | + | + | − |
| 8* | 0.58 | [M+H]^+^ | C_10_H_14_N_5_O_4_ | 268.104 6 | 0.0 | 136.063 9,119.046 2 | − | Adenosine | AL | B/PR/B/AS/Z | + | + | + |
| 9 | 0.59 | [M+H]^+^ | C_12_H_24_NO_7_ | 294.156 6 | 4.4 | 276.145 1,248.109 3,178.088 1 | − | *N*-(1-Deoxy-1-fructosyl)leucine | OA | B/M/A/Z | + | + | + |
| 10 | 0.60 | [M−H]^−^ | C_6_H_7_O_7_ | 191.018 6 | −3.1 | − | 173.010 7,129.017 1,111.008 7 | Isocitric acid | OA | AS/B/M/A/P/G/Z | + | + | + |
| 11 | 0.63 | [M+H]^+^ | C_10_H_14_N_5_O_4_ | 268.104 3 | −1.1 | 136.077 8,119.046 1 | − | 9-Arabinosyladenine | AL | B/PR/B/AS/Z | + | + | − |
| 12 | 0.64 | [M−H]^−^ | C_6_H_7_O_7_ | 191.019 1 | −0.5 | − | 173.008 2,129.019 2,111.008 5 | Citric acid | OA | AS/B/M/A/P/G/Z | + | + | + |
| 13 | 0.67 | [M+H]^+^ | C_12_H_24_NO_7_ | 294.154 2 | −3.7 | 276.146 2,248.114 3,178.074 6 | − | *N*-(1-Deoxy-1-fructosyl)leucine isomer | OA | B/M/A/Z | − | + | + |
| 14* | 1.06 | [M−H]^−^ | C_7_H_5_O_5_ | 169.014 1 | 2.4 | − | 125.024 | Gallic acid | OA | P | + | + | + |
| 15* | 1.55 | [M+H]^+^ | C_11_H_13_N_2_O_2_ | 205.096 9 | −3.9 | 188.071 5,170.061 5,146.060 9,118.066 4 | 203.082 1,159.092 3,142.065 9,116.051 3 | Tryptophan | OA | AS/G/B/M/A/Z | + | − | − |
| 16 | 2.18 | [M−H]^−^ | C_16_H_17_O_9_ | 353.086 9 | −1.1 | 163.039 | 191.055 6,179.033 6,135.046 8 | Neochlorogenic acid | OA | A | + | + | + |
| 17 | 2.50 | [M−H]^−^ | C_23_H_27_O_12_ | 495.148 7 | −3.2 | − | 137.024 2 | Oxypaeoniflorin isomer | T | P | + | + | + |
| 18 | 2.50 | [M−H]^−^ | C_23_H_27_O_14_ | 527.139 5 | −1.1 | 497.164 3 | 495.148 7,137.028 6,125.023 3 | Isomer of 6'−*O*−galloyl desbenzoylpaeoniflorin | T | P | + | + | + |
| 19 | 2.58 | [M−H]^−^ | C_10_H_9_O_5_ | 209.045 3 | 1.4 | − | 165.054 9,121.065 1,93.034 8 | Unknown | OA | G | + | + | + |
| 20 | 2.71 | [M−H]^−^ | C_23_H_27_O_12_ | 495.149 4 | −1.8 | − | 375.008 0,333.034 3,121.029 9 | Oxypaeoniflorin | T | P | + | + | + |
| 21 | 2.80 | [M−H]^−^ | C_23_H_27_O_13_S | 543.118 | 1.5 | − | 421.082 9,375.070 9,259.026 9,213.020 2,121.030 0,79.959 6 | Paeoniflorin sulfonate | T | P | + | + | + |
| 22 | 2.83 | [M−H]^−^ | C_16_H_17_O_9_ | 353.087 9 | 1.7 | 291.088 0,163.039 8,123.044 5 | 289.062 4,191.056 3,179.037 9,173.047 4 | 4-Caffeoylquinic acid | OA | A/M/AS | + | + | + |
| 23 | 2.85 | [M−H]^−^ | C_21_H_23_O_12_S | 499.091 2 | 0.4 | 257.081 9 | 453.200 4,431.091 7,417.120 0,255.066 2 | Liquiritin sulfate | Fl | G | + | + | − |
| 24* | 2.96 | [M−H]^−^ | C_16_H_17_O_9_ | 353.086 9 | −1.1 | 163.039 4 | 191.055 2,121.032 0 | Chlorogenic acid | OA | A/AS/B/M | + | + | + |
| 25* | 3.03 | [M+COOH]^−^ | C_24_H_29_O_13_ | 525.161 3 | 1.0 | 481.170 3,319.117 4,301.106 5,197.080 6,179.070 4,151.076 0,133.065 0,105.033 7 | 479.154 8,435.165 9,375.129 1,357.115 8,283.082 3,195.064 4,165.056 4,121.029 3 | Albiflorin | T | P | + | + | + |
| 26 | 3.11 | [M−H]^−^ | C_27_H_29_O_15_ | 593.151 1 | 0.8 | 595.165 5,337.070 1 | 525.162 0,345.155 9,121.028 8 | Vicenin | Fl | M/G | + | + | + |
| 27 | 3.17 | [M−H]^−^ | C_21_H_23_O_12_S | 499.091 3 | 0.6 | 257.081 8 | 417.119 1,255.065 6,135.009 5 | Isoliquiritin sulfate | Fl | G | + | + | − |
| 28 | 3.18 | [M−H]^−^ | C_26_H_31_O_16_S | 631.132 8 | −0.8 | − | 549.157 5,429.073 1,417.116 8,297.079 6,255.060 0 | Licuraside sulfate; Isoliquiritin/liquiritin Apioside sulfate; | Fl | G | + | + | − |
| 29* | 3.22 | [M+COOH]^−^ | C_24_H_29_O_13_ | 525.160 9 | 0.2 | 319.117 4,301.106 5,197.080 6,179.071 1,151.075 8,105.033 7 | 479.155 4,449.144 9,431.135 4,357.114 5,327.108 7,165.056 0,121.029 5 | Paeoniflorin | T | P | + | + | + |
| 30* | 3.36 | [M−H]^−^ | C_26_H_27_O_14_ | 563.141 5 | 2.5 | 565.154 9 | 473.104 2,443.095 7,383.079 2,353.066 1,325.068 9 | Schaftoside | Fl | G | + | + | + |
| 31 | 3.48 | [M−H]^−^ | C_26_H_27_O_14_ | 563.140 2 | 0.2 | 565.155 9,391.080 4,379.081 3,363.087 3,361.068 3 | 473.211 2,383.079 6,353.066 2,325.072 5 | Isoschaftoside | Fl | G | + | + | + |
| 32 | 3.49 | [M−H]^−^ | C_25_H_23_O_12_ | 515.118 8 | −0.4 | − | 353.090 1,191.056 7 | 1,3-Dicaffeoylquinic acid | OA | A | + | + | + |
| 33 | 3.54 | [M+H]^+^ | C_33_H_42_NO_14_ | 676.260 8 | 0.4 | 544.217 5,402.195 9,257.086 2,144.102 6,84.910 0 | 549.157 0,255.065 6,135.009 4 | Liquiritigenin-4'-O-apiosyl(1→2)−[2-(2 -piperidyl)acetyl]-glucoside | Fl | G | − | + | − |
| 34* | 3.62 | [M−H]^−^ | C_27_H_29_O_14_ | 577.156 3 | 1.0 | − | 503.134 5,487.177 8,457.118 3,439.114 5,413.091 4,383.0782353.0674 | Violanthin | Fl | G | + | + | + |
| 35 | 3.62 | [M+COOH]^−^ | C_24_H_29_O_13_ | 525.160 9 | 0.2 | 481.171 4,319.117 3,301.108 1,197.080 6,179.070 2,151.076 1,105.034 5 | 479.160 6,449.143 9,435.129 7,357.121 2,283.059 9,121.029 5 | 4-*epi*-Albiflorin | T | P | + | + | + |
| 36 | 3.66 | [M−H]^−^ | C_26_H_29_O_13_ | 549.159 4 | −2.5 | 419.136 3,257.081 1,137.024 7 | 417.116 0,135.008 0,119.048 4,91.019 1 | Neoliquiritin apioside | Fl | G | + | + | + |
| 37* | 3.71 | [M−H]^−^ | C_21_H_21_O_9_ | 417.118 5 | −0.2 | 257.082 1,137.023 5,109.029 3 | 255.065 6,135.007 6,119.049 2 | Neoliquiritin | Fl | G | + | + | + |
| 38 | 3.81 | [M−H]^−^ | C_34_H_27_O_22_ | 787.101 6 | 2.8 | − | 617.084 4,465.138 3,169.015 4,125.024 0 | 1,2,4,6-Tetragalloylglucose | O | P | + | − | − |
| 39 | 3.82 | [M−H]^−^ | C_26_H_29_O_13_ | 549.160 2 | −1.1 | 419.135 9,257.081 4,137.024 3 | 417.118 5,255.065 2,135.008 6,119.050 3,91.018 8 | Liquiritin apioside | Fl | G | + | + | + |
| 40 | 3.85 | [M+H]^+^ | C_30_H_33_O_15_ | 633.182 9 | 1.6 | 481.168 9,319.116 8,315.072 8,297.0609197.0925,179.071 8,161.062 0,153.018 6 | 631.167 6,313.056 8,177.055 5,169.014 4 | Galloylpaeoniflorin/Galloylalbiflorin | T | P | + | + | + |
| 41* | 3.86 | [M−H]^−^ | C_21_H_21_O_9_ | 417.118 1 | −1.2 | 257.081 5,137.023 9,109.029 3 | 255.065 9,135.008 8,119.050 0 | Liquiritin | Fl | G | + | + | + |
| 42 | 3.92 | [M−H]^−^ | C_27_H_29_O_16_ | 609.144 4 | −2.0 | − | 463.216 2,301.033 8,151.001 1 | Quercetin 3-*O*-neohesperidoside | Fl | B/M | + | + | + |
| 43 | 3.94 | [M−H]^−^ | C_27_H_21_O_12_ | 537.103 5 | 0.4 | − | 493.113 1,313.070 2,295.060 4,159.045 9,135.044 7,109.026 4 | Salvianolic acid J | OA | M | + | + | + |
| 44* | 3.95 | [M−H]^−^ | C_10_H_9_O_4_ | 193.049 9 | −1.0 | 195.065 1,177.054 5,151.075 9,137.060 5 | 178.027 0,149.059 7,134.036 6,121.028 0 | Ferulic acid | OA | AS | + | + | − |
| 45 | 4.07 | [M−H]^−^ | C_28_H_33_O_15_ | 609.181 7 | −0.3 | − | 301.070 6 | Hesperetin 7-O-rutinoside | Fl | M | + | + | + |
| 46 | 4.09 | [M+H]^+^ | C_23_H_29_O_11_ | 481.172 | 2.1 | 481.170 8,319.116 6,301.108 1,197.081 6,179.070 7,161.060 2,151.075 8,105.034 3 | 525.215 2,357.136 7,327.092 7,283.052 4,121.071 3 | Albiflorin R1 | T | P | + | + | + |
| 47* | 4.12 | [M−H]^−^ | C_41_H_31_O_26_ | 939.112 5 | 2.2 | 771.106 | 769.088 0,617.078 2,601.081 5,469.050 9,465.067 8,447.055 9,403.065 2,169.013 9,125.024 0 | Pentagalloylglucose | O | P | + | + | + |
| 48 | 4.14 | [M+H]^+^ | C_30_H_33_O_15_ | 633.180 8 | −1.7 | 481.182 0,319.119 0,315.082 5,297.113 1,197.082 2,179.072 2,153.019 1,161.063 0 | 631.194 8,313.068 7,177.055 7,169.068 5 | Galloylpaeoniflorin/galloylalbiflorin | T | P | + | + | + |
| 49 | 4.19 | [M+H]^+^ | C_28_H_33_O_15_ | 609.181 1 | 1.9 | 463.125 2,301.071 3 | − | Neodiosmin | Fl | M | + | + | + |
| 50* | 4.20 | [M+Na]^+^ | C_12_H_16_O_4_Na | 247.094 6 | 0.0 | 207.102 6,189.091 2,179.101 8,161.095 2 | − | Senkyunolide I | L | AS | + | + | + |
| 51 | 4.20 | [M−H]^−^ | C_25_H_23_O_12_ | 515.120 1 | 2.1 | − | 353.086 7,191.056 1 | 3,4-Dicaffeoylquinic acid | OA | A | + | + | + |
| 52 | 4.21 | [M+H]^+^ | C_33_H_42_NO_14_ | 676.257 8 | −4.0 | 545.183 5,301.073 3,257.081 4,137.061 6 | − | Liquiritigenin-4'-O-apiosyl(1→2)-[2-(2 -piperidyl)acetyl]-glucoside | Fl | G | − | − | − |
| 53* | 4.25 | [M+COOH]^−^ | C_24_H_27_O_12_ | 507.148 8 | −3.0 | 301.106 8,151.076 1,133.065 4,105.033 8 | 461.142 2,339.107 0,121.028 7 | Lactiflorin | T | P | + | + | + |
| 54 | 4.32 | [M−H]^−^ | C_21_H_21_O_10_ | 433.111 8 | −3.9 | 273.076 2 | 271.024 7,151.003 3 | 5-Hydroxyl liquiritin | Fl | G | + | + | + |
| 55* | 4.36 | [M+Na]^+^ | C_12_H_16_O_4_Na | 247.095 1 | 2.0 | 207.103 2,189.092 1,179.071 8,161.064 8 | − | Senkyunolide H | L | AS | + | + | + |
| 56 | 4.37 | [M−H]^−^ | C_25_H_23_O_12_ | 515.117 9 | −2.1 | − | 353.087 1,191.055 9 | 3,5-Dicaffeoylquinic acid | OA | A | + | + | + |
| 57* | 4.45 | [M+H]^+^ | C_22_H_23_O_9_ | 431.133 4 | −1.9 | 269.082 1,254.058 7,226.063 6,197.061 3,137.027 8 | 475.125 0,267.066 5 | Ononin | Fl | G | + | + | + |
| 58 | 4.45 | [M−H]^−^ | C_18_H_15_O_8_ | 359.077 5 | 2.2 | − | 197.046 3,161.023 5 | Labiatenic acid | OA | M | + | + | + |
| 59 | 4.50 | [M−H]^−^ | C_21_H_35_O_10_ | 447.222 8 | −0.4 | − | 269.175 4,251.165 3,221.154 1,203.142 7 | Atractyloside A (*R*/*S*) | O | A | + | + | + |
| 60 | 4.52 | [M−H]^−^ | C_25_H_23_O_12_ | 515.119 | 0.0 | − | 353.088 1,191.060 7,173.047 1 | 4,5-Dicaffeoylquinic acid | OA | A | + | + | + |
| 61 | 4.53 | [M−H]^−^ | C_21_H_33_O_10_ | 445.207 8 | 0.9 | − | 285.043 2,268.046 4,249.074 1,231.069 6,219.064 3,201.020 2 | Atractyloside A-2H | O | A | + | + | + |
| 62 | 4.54 | [M−H]^−^ | C_21_H_17_O_11_ | 445.077 1 | 0.0 | − | 891.289 8,269.045 3,175.022 6 | 5,8-Dihydroxy-4-oxo-2-phenyl-4H-chromen-7-yl hexopyranosiduronic acid | Fl | B | + | + | + |
| 63 | 4.66 | [M−H]^−^ | C_21_H_35_O_10_ | 447.222 8 | −0.4 | − | 269.037 9,251.061 2,221.066 2 | Atractyloside A (*S*/*R*) | O | A | + | + | + |
| 64* | 4.68 | [M−H]^−^ | C_26_H_29_O_13_ | 549.161 1 | 0.5 | 419.135 2,257.080 6,137.024 6 | 417.126 9,297.133 1,255.066 5,135.008 1,119.050 1,91.019 7 | Isoliquiritin apioside | Fl | G | + | + | + |
| 65 | 4.69 | [M−H]^−^ | C_21_H_33_O_10_ | 445.207 6 | 0.4 | − | 285.082 3,267.065 9,249.058 8,231.079 0,219.968 1,201.111 2 | Atractyloside A-2H | O | A | + | + | + |
| 66 | 4.74 | [M−H]^−^ | C_28_H_31_O_14_ | 591.170 8 | −1.0 | 593.186 6,285.075 9,257.078 9 | 549.158 9,296.058 9,283.061 6,255.068 1,135.009 2 | Liquiritigenin 7-(3-acetylapiofuranoside)-4'-glucoside | Fl | G | + | + | + |
| 67* | 4.85 | [M−H]^−^ | C_21_H_21_O_9_ | 417.117 9 | −1.7 | 257.081 1,137.023 7 | 255.066 2,135.008 3,119.050 8 | Isoliquiritin | Fl | G | + | + | + |
| 68 | 4.88 | [M−H]^−^ | C_36_H_37_O_16_ | 725.208 5 | 0.4 | 419.133 9,257.080 7 | 417.117 5,255.065 0,135.007 9 | Licorice-glycoside A | Fl | G | + | + | + |
| 69 | 4.94 | [M+H]^+^ | C_48_H_73_O_22_ | 1 001.456 7 | −2.6 | 825.426 9,649.392 0,631.379 5,487.340 5,469.331 9 | 999.441 6 | Licorice saponin-A3 or isomer | Tr | G | + | + | + |
| 70 | 4.99 | [M+H]^+^ | C_22_H_30_N_5_O_3_ | 412.234 9 | 0.0 | 193.044 8,179.123 4,177.059 7,163.077 1,151.046 9,137.062 1,136.062 9 | − | [6]-Zingerine | AL | Z | + | − | + |
| 71 | 5.13 | [M−H]^−^ | C_42_H_63_O_16_ | 823.408 9 | −3.3 | − | 351.056 4,193.036 0 | Uralsaponin C isomer | Tr | G | + | + | + |
| 72* | 5.17 | [M−H]^−^ | C_15_H_11_O_4_ | 255.065 4 | −1.2 | 147.047 3,137.025 0,119.049 4 | 135.008 3,119.050 6,91.017 9 | Liquiritigenin | Fl | G | + | + | + |
| 73 | 5.22 | [M+H]^+^ | C_48_H_73_O_21_ | 985.464 9 | 0.5 | 809.429 3,647.375 7,471.346 2,453.337 1 | − | Licoricesaponin A3 | Tr | G | + | + | + |
| 74 | 5.24 | [M+H]^+^ | C_42_H_63_O_18_ | 855.402 6 | 1.4 | 679.367 3,503.339 0,485.327 4,467.315 9,233.152 3 | 351.056 8,193.034 9,175.025 1,113.024 4 | 22-Hydroxy-licorice-saponin G2 | Tr | G | + | + | + |
| 75* | 5.26 | [M+COOH]^−^ | C_31_H_33_O_14_ | 629.186 7 | −0.5 | 463.160 1,445.150 0,427.138 5,319.118 3,301.106 6,267.087 3,249.075 7,197.081 2,179.071 3 | 583.180 8,553.169 1,535.158 6 | Benzoylpaeoniflorin | T | P | + | + | + |
| 76 | 5.32 | [M+COOH]^−^ | C_31_H_33_O_14_ | 629.187 6 | 1.0 | 585.197 1,463.161 3,445.148 7,427.139 7,319.119 4,301.108 4,267.087 4,249.077 7,197.081 1,179.070 8,105.033 8 | 583.183 3,553.171 0,535.166 8 | Paeonidaninol A/B | T | P | + | + | + |
| 77 | 5.37 | [M−H]^−^ | C_44_H_63_O_19_ | 895.395 5 | −1.0 | 721.381 5,545.348 7,527.337 3,509.326 3,485.327 4,467.316 5,449.306 2,421.311 2,403.299 8 | 351.057 1,193.035 2,175.025 9,113.024 1 | Uralsaponin F | Tr | G | + | + | + |
| 78 | 5.41 | [M−H]^−^ | C_42_H_61_O_16_ | 821.396 5 | 0.6 | 471.348 1,453.336 3 | 645.367 9,351.056 3,193.036 3,175.024 2,113.025 3 | Uralsaponin B | Tr | G | + | + | + |
| 79 | 5.44 | [M−H]^−^ | C_18_H_31_O_5_ | 327.216 8 | −1.1 | 293.211 2,275.201 0,257.190 8,229.195 7 | 193.033 6 | Trihydroxyoctadecadienoic acid | OA | M | + | + | + |
| 80 | 5.55 | [M−H]^−^ | C_42_H_61_O_17_ | 837.388 8 | −2.5 | 663.373 0,487.343 7,469.332 0,451.320 0,433.310 2,423.324 6,405.315 4 | 351.056 8,193.034 8,175.024 3,113.024 5 | Macedonoside A | Tr | G | + | + | + |
| 81 | 5.60 | [M+H]^+^ | C_42_H_61_O_17_ | 837.392 2 | 1.6 | 661.352 7,485.327 0,467.314 5 | − | 24-Hydroxy-licorice-saponin E2 | Tr | G | + | + | + |
| 82 | 5.67 | [M−H]^−^ | C_44_H_63_O_18_ | 879.399 8 | −1.8 | 705.382 6,529.354 1,511.342 5,493.330 5,451.321 6,469.330 2,433.307 9,405.316 1 | 351.056 2,193.035 3,175.025 1,113.024 3 | Acetoxyl-glycyrrhizin | Tr | G | + | + | + |
| 83 | 5.75 | [M−H]^−^ | C_18_H_33_O_5_ | 329.232 7 | −0.3 | 353.228 9,313.237 6,295.226 8,277.216 3,195.138 5,149.132 9 | 329.232 7 | Trihydroxyoctadecenoic acid | OA | AS/B/M/A/P/G/Z/PR | + | + | + |
| 84 | 5.83 | [M+H]^+^ | C_12_H_13_O_2_ | 189.092 1 | 2.6 | 171.082 2,147.117 2,133.031 4 | − | Ligusticum lactone | L | AS | + | + | + |
| 85 | 5.89 | [M−H]^−^ | C_42_H_59_O_16_ | 819.379 3 | −1.2 | 645.363 7,469.331 9,451.321 4,433.310 5 | 351.056 7,193.034 9,175.025 0,113.023 5 | Licoricesaponin E2 | Tr | G | + | + | + |
| 86 | 6.02 | [M−H]^−^ | C_44_H_63_O_17_ | 863.407 5 | 1.2 | 689.390 1,513.357 8,495.347 3 | 687.193 8,351.057 4,193.036 0 | Acetoxyl-glycyrrhaldehyde | Tr | G | + | + | + |
| 87 | 6.03 | [M−H]^−^ | C_42_H_61_O_17_ | 837.392 5 | 1.9 | 663.374 1,645.363 6,627.351 7,487.342 1,469.331 6,451.321 0,433.310 1,423.326 3,405.315 2 | 351.056 9,193.034 7,175.023 8,113.024 9 | Licoricesaponin G2 | Tr | G | + | + | + |
| 88 | 6.13 | [M+H]^+^ | C_48_H_75_O_21_ | 987.478 1 | −2.0 | 841.416 9,665.386 8,453.336 2 | − | Yunganoside G1 or isomer | Tr | G | + | + | + |
| 89 | 6.19 | [M−H]^−^ | C_42_H_61_O_17_ | 837.390 4 | −0.6 | 663.374 1,645.361 8,627.353 0,487.342 3,469.331 9,451.320 0,433.310 5,423.324 5,405.313 9 | 351.057 3,193.035 8,175.024 1,113.023 4 | Uralsaponin U/N | Tr | G | + | + | + |
| 90 | 6.25 | [M+H]^+^ | C_48_H_75_O_21_ | 987.477 4 | −2.7 | 841.422 5,453.335 0 | 839.404 4,807.416 0 | Yunganoside G1 or isomer | Tr | G | + | + | + |
| 91 | 6.30 | [M−H]^−^ | C_42_H_61_O_16_ | 821.396 5 | 0.6 | 647.378 8,471.346 4,453.335 8 | 351.056 2,193.035 5,175.024 6,113.023 5 | Licorice saponin H2 | Tr | G | + | + | + |
| 92 | 6.32 | [M+H]^+^ | C_44_H_67_O_18_ | 883.432 6 | −0.1 | 707.417 7,531.175 1,513.386 0,471.348 5,453.337 9,435.328 3 | − | 22-Acetoxylicoricesaponin J2 | Tr | G | + | + | + |
| 93* | 6.33 | [M+H]^+^ | C_16_H_13_O_4_ | 269.081 4 | −0.4 | 254.057 5,253.050 3,226.0632197.0610,169.066 3 | − | Formonentin | Fl | G | + | + | + |
| 94* | 6.35 | [M−H]^−^ | C_42_H_61_O_16_ | 821.397 2 | 1.2 | 647.378 7,471.346 9,453.336 3,407.330 3 | 351.057 3,193.035 0,175.025 2,113.024 3 | 18*α*-Glycyrrhizin | Tr | G | + | + | + |
| 95 | 6.37 | [M−H]^−^ | C_42_H_61_O_16_ | 821.396 9 | 1.1 | 647.378 8,471.346 6,453.337 3,407.330 1 | 351.057 3,193.035 8,175.025 2,113.024 3 | 18*β*-Glycyrrhizin | Tr | G | + | + | + |
| 96 | 6.38 | [M+H]^+^ | C_42_H_65_O_15_ | 809.439 9 | −3.0 | 633.400 3,457.366 2,439.357 5 | − | 22-Dehydroxy-uralsaponin C | Tr | G | + | + | + |
| 97 | 6.41 | [M−H]^−^ | C_42_H_61_O_16_ | 821.396 2 | 0.2 | 647.377 6,471.347 0,453.336 3,437.341 3,407.330 2 | 351.056 6,193.035 3,175.024 7,113.024 8 | Licorice-saponin K2 | Tr | G | + | + | + |
| 98* | 6.43 | [M+H]^+^ | C_42_H_69_O_13_ | 781.475 1 | 1.7 | 763.498 3,745.426 7,619.423 2,601.410 3,583.394 0,473.353 2,455.344 0,419.330 5 | 779.457 6,617.405 8, | Saikosaponin A | Tr | B | + | + | + |
| 99 | 6.47 | [M+H]^+^ | C_21_H_21_O_6_ | 369.134 2 | 1.1 | 313.068 9,298.997 7,285.221 8,271.205 5,227.181 0,211.004 43,200.052 7,137.060 1 | 367.118 4 | Glicoricone | Fl | G | + | + | + |
| 100 | 6.49 | [M+COOH]^−^ | C_43_H_69_O_15_ | 825.463 9 | 0.4 | 455.352 7,437.340 5,419.331 2 | 779.458 0,617.406 1 | Iso-saikosaponin A | Tr | B | + | + | + |
| 101* | 6.53 | [M+Na]^+^ | C_17_H_26_O_4_Na | 317.172 3 | −1.9 | 277.181 7,259.175 2,179.072 2,177.092 6,137.060 8 | − | [6]-Gingerol | O | Z | + | + | + |
| 102 | 6.67 | [M+COOH]^−^ | C_43_H_69_O_15_ | 825.466 7 | 3.8 | 455.350 6,437.343 1,419.331 5 | 779.462 3,617.410 4 | Saikosaponin B1/B2 | Tr | B | + | − | + |
| 103* | 6.71 | [M−H]^−^ | C_15_H_11_O_4_ | 255.066 1 | 1.6 | 147.043 0,137.025 4,119.052 4 | 135.007 9,119.047 6,91.019 5 | Isoliquiritigenin | Fl | G | + | + | + |
| 104 | 6.74 | [M+H]^+^ | C_12_H_17_O_2_ | 193.123 | 0.5 | 175.148 5,147.116 3,137.062 8 | − | Senkyunolide | L | AS | + | + | − |
| 105 | 6.75 | [M+COOH]^−^ | C_43_H_69_O_15_ | 825.466 5 | 3.5 | 455.352 5,437.341 7,419.331 3 | 779.454 8,617.404 8 | Saikosaponin B1/B2 | Tr | B | + | − | + |
| 106* | 6.77 | [M+H]^+^ | C_15_H_21_O_3_ | 249.149 6 | 2.0 | 231.138 0,203.143 8,163.077 9,119.086 4,105.070 3,91.057 6 | − | Atractylenolide III | L | A | + | − | − |
| 107 | 6.85 | [M−H]^−^ | C_12_H_11_O_3_ | 203.070 7 | −0.5 | 187.075 2,149.022 5 | 193.035 8 | Senkyunolide C or 3-Butylidene-7-hydroxyphthalide | L | AS | + | + | − |
| 108 | 6.86 | [M+H]^+^ | C_48_H_77_O_19_ | 957.504 3 | −1.7 | 811.447 8,617.396 9,441.371 3,423.364 0 | − | Yunganoside A1/B1/C1 | Tr | G | + | + | + |
| 109 | 7.03 | [M−H]^−^ | C_18_H_31_O_4_ | 311.222 3 | 0.3 | 295.227 5,277.216 7,259.206 7,231.211 3 | − | Dihydroxyoctadecadienoic acid | OA | B,M | + | + | + |
| 110 | 7.06 | [M−H]^−^ | C_44_H_69_O_14_ | 821.470 2 | 1.8 | 823.481 9,805.473 1,455.352 0,437.341 9,419.330 1 | 779.454 9,761.457 8,617.405 1 | Acetylsaikosaponin | Tr | B | + | + | + |
| 111* | 7.09 | [M+H]^+^ | C_21_H_21_O_6_ | 369.134 | 0.5 | 351.296 4,313.254 0,298.274 6,285.081 1,271.055 6,270.049 7,255.065 1 | 367.118 2 | Glycycoumarin | C | G | + | + | + |
| 112 | 7.13 | [M−H]^−^ | C_42_H_63_O_16_ | 823.413 9 | 2.3 | − | 647.387 3,471.355 8,351.058 2,193.033 4 | Licoricesaponin J2 | Tr | G | + | + | + |
| 113 | 7.20 | [M+H]^+^ | C_21_H_25_O_5_ | 357.171 6 | 3.9 | 301.106 9,221.114 3,191.107 7,165.057 1,123.046 9 | 355.154 6 | Glyasperin C | Fl | G | + | + | + |
| 114* | 7.22 | [M+H]^+^ | C_16_H_13_O_5_ | 285.076 3 | 1.8 | 270.052 4,242.057 5,153.018 3 | 283.061 2,211.039 6 | Acacetin | Fl | M | + | + | + |
| 115 | 7.26 | [M−H]^−^ | C_12_H_11_O_3_ | 203.070 2 | −3.0 | 187.075 0,149.023 0 | 113.025 8 | Senkyunolide C or 3-Butylidene-7-hydroxyphthalide | L | AS | + | + | − |
| 116 | 7.27 | [M−H]^−^ | C_36_H_53_O_10_ | 645.363 2 | −1.1 | 471.349 2,453.336 9,435.326 4,407.332 9 | 469.331 7,351.058 9 | Starrhizin | Tr | G | + | + | + |
| 117 | 7.45 | [M−H]^−^ | C_42_H_63_O_15_ | 807.419 6 | 3.6 | − | 631.378 8,351.053 3,193.039 7 | 22-Dehydroxy-uralsaponin C | Tr | G | + | + | + |
| 118* | 7.47 | [M+H]^+^ | C_12_H_15_O_2_ | 191.107 6 | 2.1 | 173.095 8,163.114 1,145.105 1,117.077 5,103.227 5,91.058 6 | − | Ligustilide | L | AS | + | + | + |
| 119 | 7.48 | [M−H]^−^ | C_18_H_33_O_4_ | 313.238 8 | 2.9 | 297.243 3,279.232 0,261.222 4 | − | Dihydroxyoctadecenoic acid | OA | AS/B/M/A/Z | + | − | − |
| 120 | 7.51 | [M+H]^+^ | C_20_H_17_O_6_ | 353.103 1 | 1.7 | 311.129 2,298.250 8,283.024 2,153.016 2 | 351.086 7 | Licoisoflavone B or Semilicoisoflavone B or Allolicoisoflavone B | Fl | G | + | + | + |
| 121 | 7.61 | [M+H]^+^ | C_20_H_19_O_6_ | 355.117 9 | −0.8 | 299.066 3,287.223 0,201.173 1,153.053 0 | − | Uralenin | Fl | G | + | + | + |
| 122 | 7.62 | [M+H]^+^ | C_15_H_21_O_2_ | 233.154 7 | 2.1 | 215.155 7,186.221 2,159.111 9,151.077 5,133.059 1 | − | Atractylenolide II | L | A | + | − | + |
| 123 | 8.22 | [M+H]^+^ | C_24_H_51_NO_7_P | 496.341 | 1.4 | 184.074 8 | − | 1-Palmitoylphosphatidylcholine | O | AS/A/P | + | − | − |
| 124* | 8.29 | [M+H]^+^ | C_15_H_19_O_2_ | 231.139 3 | 3.5 | 185.132 8 | − | Atractylenolide I | L | A | + | − | − |

Notes: * represents compounds were identified by comparing with reference standards. “+” indicates that the compound was detected in the corresponding preparation, whereas “−” indicates that the compound was not detected. B, AS, P, A, PR, M, Z, G represents the following Chinese herbal medicines: *Bupleuri Radix*, *Angelicae Sinensis Radix*, *Paeoniae Radix Alba*, *Atractylodis Macrocephalae Rhizoma*, *Poria*, *Menthae Haplocalycis Herba*, *Zingiberis Rhizoma Recens*, and *Glycyrrhizae Radix* et *Rhizoma*. Fl, T, Tr, OA, AL, L, C, O represent the following categories of compounds: flavonoids, terpenoids, triterpenoids, organic acids, alkaloids, lactones, coumarins, and others.

**Table S3** Content of 31 compounds in 22 batches of XYS formulations.

| Analytes | H1 | H2 | H3 | W1 | W2 | W3 | W4 | W5 | W6 | W7 | W8 | W9 | W10 | W11 | K1 | K2 | K3 | K4 | K5 | K6 | K7 | K8 |
| --- | --- | --- | --- | --- | --- | --- | --- | --- | --- | --- | --- | --- | --- | --- | --- | --- | --- | --- | --- | --- | --- | --- |
| Gallic acid | 198.33 | 161.79 | 150.48 | 249.39 | 285.41 | 269.18 | 287.80 | 249.84 | 259.72 | 265.07 | 213.57 | 243.97 | 256.26 | 266.27 | 438.42 | 307.56 | 535.04 | 449.94 | 336.00 | 333.91 | 417.76 | 471.71 |
| Tryptophan | 26.18 | 26.74 | 23.77 | 6.13 | 4.11 | 5.51 | 5.04 | 5.99 | 5.62 | 5.54 | 4.05 | 4.70 | 4.53 | 6.13 | 3.28 | 2.91 | 12.52 | 6.29 | 3.74 | 3.20 | 5.01 | 8.69 |
| Chlorogenic acid | 34.52 | 27.80 | 31.17 | 88.24 | 109.03 | 107.48 | 97.52 | 101.39 | 111.57 | 116.66 | 96.78 | 94.91 | 113.48 | 119.10 | 48.81 | 27.47 | 37.95 | 41.62 | 28.28 | 30.38 | 38.15 | 39.10 |
| Albiflorin | 814.13 | 903.03 | 925.96 | 1 205.25 | 1 351.11 | 1 361.65 | 1 291.94 | 1 359.82 | 1 388.61 | 1 483.29 | 1 153.84 | 1 183.65 | 1 392.10 | 1 136.29 | 687.25 | 483.19 | 958.40 | 731.92 | 630.02 | 515.44 | 679.25 | 884.42 |
| Paeoniflorin | 1 283.16 | 1 377.18 | 1 406.86 | 1 670.88 | 1 596.44 | 1 586.11 | 1 804.84 | 1 621.36 | 1 636.12 | 1 729.42 | 1 931.41 | 1 470.11 | 1 618.94 | 1 700.87 | 618.63 | 413.67 | 691.18 | 667.19 | 517.61 | 416.80 | 636.84 | 686.13 |
| Schaftoside | 18.13 | 12.52 | 16.38 | 10.79 | 9.11 | 10.97 | 12.35 | 12.16 | 10.81 | 12.40 | 8.59 | 7.90 | 10.67 | 11.80 | 10.19 | 5.97 | 9.29 | 9.43 | 6.18 | 6.52 | 8.69 | 8.87 |
| Violanthin | 15.76 | 12.14 | 13.55 | 11.24 | 9.44 | 11.01 | 14.18 | 13.01 | 11.27 | 13.02 | 10.31 | 10.19 | 12.27 | 12.21 | 8.87 | 6.34 | 8.88 | 9.23 | 5.95 | 6.71 | 8.87 | 8.36 |
| Neoliquiritin | 25.27 | 16.51 | 22.23 | 9.29 | 5.76 | 5.60 | 12.54 | 7.83 | 6.39 | 7.67 | 9.22 | 10.05 | 7.40 | 9.53 | 8.21 | 5.99 | 10.65 | 10.28 | 6.32 | 6.22 | 9.42 | 9.90 |
| Liquiritin apioside | 216.50 | 143.81 | 188.50 | 116.06 | 100.51 | 112.82 | 140.50 | 130.55 | 123.36 | 130.99 | 90.05 | 117.24 | 127.20 | 128.91 | 93.42 | 63.20 | 102.21 | 93.30 | 60.31 | 66.76 | 88.50 | 95.74 |
| Liquiritin | 165.60 | 148.92 | 164.81 | 92.23 | 120.14 | 151.11 | 127.91 | 144.43 | 172.64 | 145.45 | 80.04 | 94.33 | 144.89 | 112.45 | 89.47 | 54.54 | 83.77 | 78.68 | 60.33 | 56.63 | 71.42 | 76.77 |
| Ferulic acid | 57.35 | 29.69 | 45.03 | 52.50 | 48.77 | 53.12 | 55.19 | 56.62 | 58.08 | 52.60 | 50.82 | 42.22 | 46.11 | 41.88 | 11.58 | 9.01 | 33.69 | 11.69 | 10.51 | 10.08 | 10.77 | 29.95 |
| Pentagalloylglucose | 131.48 | 86.21 | 130.33 | 22.99 | 24.21 | 25.46 | 27.32 | 23.65 | 23.80 | 24.29 | 25.46 | 23.03 | 24.09 | 24.78 | 30.15 | 22.64 | 29.98 | 28.91 | 26.48 | 22.99 | 33.50 | 28.49 |
| Senkyunolide I | 22.67 | 16.50 | 21.00 | 19.14 | 14.65 | 17.80 | 20.06 | 19.57 | 21.20 | 18.69 | 19.33 | 19.33 | 18.35 | 15.12 | 7.20 | 7.86 | 7.26 | 9.54 | 6.27 | 8.15 | 8.43 | 6.41 |
| Lactiflorin | 12.47 | 10.22 | 11.17 | 114.92 | 104.45 | 124.96 | 120.43 | 112.71 | 114.94 | 112.67 | 61.23 | 100.27 | 107.03 | 83.58 | 29.77 | 17.11 | 20.03 | 24.26 | 21.05 | 19.88 | 26.28 | 20.10 |
| Senkyunolide H | 4.27 | 3.00 | 4.01 | 3.64 | 2.75 | 3.27 | 3.87 | 3.64 | 4.00 | 3.75 | 3.66 | 3.52 | 3.44 | 2.93 | 1.50 | 1.45 | 1.50 | 1.89 | 1.24 | 1.68 | 1.57 | 1.34 |
| Ononin | 14.21 | 11.84 | 16.31 | 9.76 | 7.68 | 8.76 | 12.07 | 9.62 | 8.84 | 9.60 | 7.60 | 9.50 | 8.45 | 10.12 | 8.50 | 5.68 | 8.02 | 9.25 | 6.45 | 6.09 | 8.58 | 7.27 |
| Isoliquiritin apioside | 26.96 | 13.22 | 21.63 | 23.62 | 15.76 | 21.58 | 29.99 | 26.66 | 22.47 | 25.15 | 19.68 | 27.19 | 25.37 | 24.20 | 14.49 | 9.19 | 16.89 | 17.11 | 9.88 | 10.63 | 14.24 | 14.47 |
| Isoliquiritin | 24.76 | 15.74 | 19.98 | 17.46 | 22.13 | 33.90 | 24.31 | 30.13 | 33.65 | 31.28 | 15.93 | 18.71 | 27.52 | 19.42 | 14.77 | 9.80 | 17.31 | 16.63 | 11.55 | 11.23 | 13.18 | 15.09 |
| Liquiritigenin | 15.57 | 7.78 | 10.08 | 14.70 | 17.24 | 22.06 | 15.23 | 20.57 | 22.02 | 20.30 | 9.80 | 7.11 | 16.80 | 13.94 | 9.94 | 5.22 | 13.24 | 5.78 | 5.11 | 5.73 | 5.76 | 12.68 |
| Benzoylpaeoniflorin | 40.49 | 37.12 | 39.17 | 52.28 | 53.47 | 64.30 | 59.26 | 67.19 | 62.30 | 68.36 | 67.82 | 49.07 | 62.66 | 56.22 | 17.83 | 11.45 | 20.44 | 19.45 | 14.80 | 12.08 | 18.45 | 19.65 |
| Formononetin | 0.79 | 0.28 | 0.52 | 1.18 | 0.69 | 0.88 | 1.11 | 1.13 | 0.89 | 0.94 | 0.65 | 0.60 | 0.89 | 0.96 | 0.70 | 0.41 | 0.73 | 0.54 | 0.42 | 0.44 | 0.50 | 0.70 |
| [6]-Gingerol | 1.15 | 1.04 | 1.68 | 1.25 | 1.27 | 1.66 | 0.74 | 1.79 | 1.41 | 1.11 | 1.10 | 1.20 | 1.18 | 2.22 | 1.18 | 0.94 | 2.08 | 1.10 | 0.79 | 1.04 | 0.91 | 2.80 |
| Saikosaponin A | 3.10 | 1.85 | 1.93 | 3.33 | 2.65 | 8.39 | 4.63 | 6.07 | 5.15 | 4.37 | 2.19 | 5.12 | 2.98 | 4.83 | 4.97 | 1.75 | 5.70 | 4.32 | 2.48 | 1.85 | 3.45 | 4.79 |
| Glycyrrhizin | 591.82 | 498.20 | 541.37 | 393.44 | 352.28 | 405.67 | 484.27 | 457.49 | 399.43 | 475.87 | 354.96 | 430.11 | 395.92 | 403.29 | 396.39 | 224.66 | 355.41 | 306.61 | 225.48 | 210.09 | 285.73 | 344.02 |
| Atractylenolide III | 7.94 | 5.34 | 6.08 | 4.24 | 2.15 | 7.04 | 4.23 | 5.77 | 5.91 | 6.75 | 4.10 | 4.87 | 5.80 | 5.66 | 1.39 | 0.55 | 1.18 | 1.11 | 0.71 | 0.56 | 0.85 | 1.11 |
| Isoliquiritigenin | 3.27 | 1.45 | 2.02 | 1.75 | 1.77 | 2.61 | 1.96 | 2.35 | 2.61 | 1.99 | 1.20 | 0.79 | 1.80 | 1.44 | 2.37 | 1.24 | 3.18 | 1.52 | 1.19 | 1.37 | 1.32 | 2.92 |
| Glycycoumarin | 0.18 | 0.10 | 0.10 | 0.17 | 0.13 | 0.37 | 0.18 | 0.22 | 0.27 | 0.24 | 0.13 | 0.04 | 0.18 | 0.22 | 0.28 | 0.16 | 0.41 | 0.23 | 0.18 | 0.23 | 0.19 | 0.34 |
| Acacetin | 0.10 | 0.05 | 0.07 | 0.01 | 0.01 | 0.01 | 0.01 | 0.01 | 0.01 | 0.01 | 0.01 | 0.01 | 0.01 | 0.01 | 0.07 | 0.04 | 0.07 | 0.11 | 0.11 | 0.05 | 0.10 | 0.08 |
| Ligustilide | 33.28 | 7.81 | 18.68 | 306.18 | 310.88 | 492.36 | 430.90 | 375.99 | 487.23 | 400.76 | 331.35 | 271.28 | 319.79 | 382.73 | 5.68 | 6.47 | − | − | − | − | − | 4.47 |
| Atractylenolide II | 0.75 | 0.33 | 0.44 | 0.54 | 0.27 | 0.92 | 0.60 | 0.75 | 0.81 | 0.78 | 0.45 | 0.59 | 0.67 | 0.65 | 0.18 | 0.13 | 0.24 | 0.20 | 0.17 | 0.14 | 0.11 | 0.19 |
| Atractylenolide I | 0.17 | 0.06 | 0.07 | 0.02 | 0.02 | 0.04 | 0.03 | 0.03 | 0.04 | 0.03 | 0.02 | 0.03 | 0.03 | 0.03 | 0.01 | 0.01 | 0.01 | 0.01 | 0.01 | 0.01 | 0.01 | 0.01 |

Notes: H1−3, W1−11, K1−8 represent different batches of XYHJ, XYW, and XYKL, respectively. The unit of content is µg/g. For quantitative analysis of 31 components, UPLC-QqQ-MS/MS was applied for quantitative analysis. Chromatographic conditions achieved using a Waters ACQUITY™ RP C_18_ column (2.1 mm × 100 mm, 1.7 µm) with a flow rate of 0.3 mL/min. The mobile phase consisted of A (water with 0.1% formic acid) and B (acetonitrile with 0.1% formic acid). The gradient program was: 0−1.0 min (2% B), 1.0−1.5 min (2%−5% B), 1.5−5.5 min (5%−30% B), 5.5−9.0 min (30%−40% B), 9.0−11.5 min (40%−100% B), 11.5−12.5 min (100% B), 12.5−13.0 min (100%−2% B), and 13.0−14.0 min (2% B). The MS conditions utilized an ESI source with voltages of -3.0 kV (negative) and 2.0 kV (positive), ion source temperature of 150 °C, and desolvation temperature of 600 °C. Data was acquired in MRM and SIM modes.

**Table S4** Linearity of 15 quantified compounds.

| Compounds | Regression equations | *R*² | Linear range (μg/mL) |
| --- | --- | --- | --- |
| Gallic acid | *y* = 33 892 *x* + 38 457 | 0.999 5 | 9.43−382.00 |
| Chlorogenic acid | *y* = 17 102 *x* + 1 651.9 | 0.999 6 | 1.26−50.95 |
| Albiflorin | *y* = 2 275.4 *x* + 6 138.8 | 0.999 6 | 33.55−1358.70 |
| Paeoniflorin | *y* = 2 642.6 *x* + 48 604 | 0.999 4 | 90.74−3674.46 |
| Schaftoside | *y* = 16 897 *x* – 6 458.3 | 0.999 8 | 4.55−184.20 |
| Ferulic acid | *y* = 6 392.3 *x* + 2 234.7 | 0.999 7 | 1.99−80.60 |
| Liquiritin apioside | *y* = 1 263.2 *x* + 1 483.7 | 0.999 8 | 7.61−308.32 |
| Liquiritin | *y* = 1 461.1 *x* + 2 228 | 0.999 8 | 13.63−551.80 |
| Pentagalloyl glucose | *y* = 36 393 *x* + 8 968.4 | 0.999 6 | 5.48−222.00 |
| Senkyunolide I | *y* = 68 883 *x* + 2 557.3 | 0.999 8 | 1.52−61.38 |
| Lactiflorin | *y* = 1 309.7 *x* − 1 959.6 | 0.999 7 | 20.09−813.44 |
| Senkyunolide H | *y* = 61 358 *x* + 3 245.8 | 0.999 8 | 0.72−29.22 |
| Ononin | *y* = 23 002 *x* + 986.26 | 0.999 7 | 1.70−68.880 |
| Liquiritigenin | *y* = 3 9617 *x* − 951.3 | 0.999 7 | 0.80−32.20 |
| Glycyrrhizic acid | *y* = 7 045.2 *x* + 2 6051 | 0.999 5 | 27.54−1115.20 |

**Table S5** Precision of 15 quantified compounds.

| Compounds | Intra-day (*n*=6) | | Inter-day (*n*=18) | |
| --- | --- | --- | --- | --- |
|  | Mean ± SD (μg/g) | RSD (%) | Mean ± SD (μg/g) | RSD (%) |
| Gallic acid | 225.73 ± 4.76 | 2.11 | 225.22 ± 3.09 | 1.37 |
| Chlorogenic acid | 52.24 ± 1.65 | 3.16 | 53.75 ± 3.22 | 5.99 |
| Albiflorin | 989.75 ± 17.45 | 1.76 | 978.17 ± 33.26 | 3.40 |
| Paeoniflorin | 2 503.47 ± 53.84 | 2.15 | 2 508.71 ± 56.98 | 2.27 |
| Schaftoside | 147.13 ± 2.58 | 1.76 | 148.25 ± 4.14 | 2.79 |
| Ferulic acid | 25.73 ± 0.24 | 0.92 | 25.62 ± 0.57 | 2.24 |
| Liquiritin apioside | 153.28 ± 2.55 | 1.67 | 150.98 ± 4.01 | 2.66 |
| Liquiritin | 253.31 ± 4.43 | 1.75 | 250.57 ± 4.97 | 1.98 |
| Pentagalloyl glucose | 163.84±3.05 | 1.86 | 160.36 ± 3.80 | 2.37 |
| Senkyunolide I | 38.41 ± 0.77 | 2.02 | 36.58 ± 1.58 | 4.33 |
| Lactiflorin | 482.57±15.87 | 3.29 | 499.84 ± 31.10 | 6.22 |
| Senkyunolide H | 14.06 ± 0.38 | 2.74 | 14.01 ± 0.50 | 3.59 |
| Ononin | 39.60 ± 1.86 | 4.69 | 35.98 ± 2.95 | 8.21 |
| Liquiritigenin | 24.03 ± 0.82 | 3.41 | 25.22 ± 1.76 | 7.00 |
| Glycyrrhizic acid | 743.89 ± 14.02 | 1.89 | 741.64 ± 9.33 | 1.26 |

**Table S6** Stability of 15 quantified compounds.

| Compounds | 0 h (μg/g) | 2 h (μg/g) | 4 h (μg/g) | 8 h (μg/g) | 12 h (μg/g) | 24 h (μg/g) | Mean (μg/g) | RSD (%) |
| --- | --- | --- | --- | --- | --- | --- | --- | --- |
| Gallic acid | 230.36 | 227.53 | 227.75 | 228.23 | 228.47 | 228.38 | 228.45 | 0.40 |
| Chlorogenic acid | 47.55 | 51.17 | 51.06 | 51.19 | 52.29 | 51.53 | 50.80 | 2.97 |
| Albiflorin | 987.19 | 980.36 | 984.81 | 982.53 | 990.49 | 986.13 | 985.25 | 0.33 |
| Paeoniflorin | 2 545.74 | 2 525.76 | 2 537.72 | 2 534.75 | 2 555.26 | 2 549.12 | 2 541.39 | 0.38 |
| Schaftoside | 149.31 | 149.25 | 151.54 | 150.33 | 151.90 | 151.16 | 150.58 | 0.69 |
| Ferulic acid | 26.16 | 25.29 | 25.72 | 25.45 | 25.29 | 25.57 | 25.58 | 1.17 |
| Liquiritin apioside | 152.46 | 151.82 | 152.70 | 152.69 | 152.76 | 153.35 | 152.63 | 0.30 |
| Liquiritin | 255.48 | 253.05 | 253.11 | 253.79 | 254.85 | 255.08 | 254.23 | 0.38 |
| Pentagalloyl glucose | 155.05 | 156.68 | 157.04 | 155.57 | 156.44 | 157.66 | 156.40 | 0.56 |
| Senkyunolide I | 36.70 | 35.59 | 35.75 | 35.74 | 35.67 | 35.94 | 35.90 | 1.04 |
| Lactiflorin | 489.77 | 475.35 | 479.57 | 483.17 | 489.31 | 490.87 | 484.67 | 1.19 |
| Senkyunolide H | 13.90 | 13.49 | 13.73 | 13.79 | 13.65 | 13.77 | 13.72 | 0.93 |
| Ononin | 35.18 | 33.11 | 33.66 | 33.68 | 33.75 | 33.73 | 33.85 | 1.86 |
| Liquiritigenin | 21.57 | 23.64 | 24.35 | 24.24 | 24.62 | 24.41 | 23.80 | 4.38 |
| Glycyrrhizic acid | 733.61 | 732.93 | 735.92 | 735.49 | 736.91 | 738.46 | 735.55 | 0.25 |

**Table S7** Recovery evaluation of 15 quantified compounds.

| Compounds | Endogenous (μg) | Spiked (μg) | Detected (μg) | Recovery (%) | Mean (%) | RSD (%) |
| --- | --- | --- | --- | --- | --- | --- |
| Gallic acid | 72.10 | 68.03 | 146.05 | 108.70 | 107.04 | 1.67 |
|  | 72.10 | 68.21 | 145.86 | 108.14 |  |  |
|  | 71.92 | 68.25 | 143.64 | 105.08 |  |  |
|  | 72.13 | 68.46 | 143.36 | 104.04 |  |  |
|  | 71.05 | 68.54 | 145.12 | 108.07 |  |  |
|  | 71.25 | 68.32 | 145.20 | 108.22 |  |  |
| Chlorogenic acid | 10.10 | 18.19 | 28.61 | 101.77 | 103.51 | 3.12 |
|  | 9.26 | 18.23 | 28.12 | 103.45 |  |  |
|  | 8.84 | 18.18 | 28.59 | 108.65 |  |  |
|  | 9.26 | 18.15 | 28.44 | 105.65 |  |  |
|  | 9.85 | 18.29 | 28.76 | 103.43 |  |  |
|  | 8.78 | 18.24 | 26.68 | 98.15 |  |  |
| Albiflorin | 292.60 | 300.89 | 594.52 | 100.34 | 95.20 | 2.55 |
|  | 289.04 | 302.27 | 568.83 | 92.56 |  |  |
|  | 285.74 | 303.55 | 571.64 | 94.18 |  |  |
|  | 284.74 | 304.05 | 572.54 | 94.65 |  |  |
|  | 307.20 | 306.28 | 596.66 | 94.51 |  |  |
|  | 310.36 | 303.83 | 598.79 | 94.93 |  |  |
| Paeoniflorin | 814.79 | 775.78 | 1578.43 | 98.43 | 96.26 | 2.58 |
|  | 814.19 | 777.25 | 1527.56 | 91.78 |  |  |
|  | 813.05 | 779.71 | 1555.85 | 95.27 |  |  |
|  | 806.18 | 780.18 | 1553.70 | 95.81 |  |  |
|  | 781.36 | 783.32 | 1561.35 | 99.57 |  |  |
|  | 779.70 | 779.90 | 1533.88 | 96.70 |  |  |
| Schaftoside | 47.12 | 42.07 | 86.17 | 92.80 | 95.65 | 3.92 |
|  | 46.98 | 41.94 | 86.34 | 93.83 |  |  |
|  | 47.04 | 42.08 | 86.52 | 93.82 |  |  |
|  | 46.95 | 42.10 | 86.49 | 93.91 |  |  |
|  | 47.08 | 42.19 | 87.47 | 95.73 |  |  |
|  | 46.45 | 42.20 | 90.25 | 103.81 |  |  |
| Ferulic acid | 11.69 | 14.01 | 25.95 | 101.72 | 102.50 | 3.73 |
|  | 11.55 | 14.03 | 25.28 | 97.86 |  |  |
|  | 11.41 | 14.05 | 25.44 | 99.82 |  |  |
|  | 11.25 | 14.04 | 25.41 | 100.78 |  |  |
|  | 10.79 | 14.09 | 25.65 | 105.48 |  |  |
|  | 10.29 | 14.05 | 25.65 | 109.31 |  |  |
| Liquiritin apioside | 50.63 | 58.33 | 113.87 | 108.42 | 104.87 | 1.87 |
|  | 50.44 | 58.50 | 110.53 | 102.72 |  |  |
|  | 50.00 | 58.48 | 110.78 | 103.93 |  |  |
|  | 49.80 | 58.46 | 110.05 | 103.06 |  |  |
|  | 49.39 | 58.72 | 111.00 | 104.91 |  |  |
|  | 48.91 | 58.54 | 111.06 | 106.18 |  |  |
| Liquiritin | 82.26 | 119.25 | 208.82 | 106.13 | 104.51 | 1.19 |
|  | 82.12 | 119.41 | 205.41 | 103.25 |  |  |
|  | 81.77 | 119.58 | 205.68 | 103.62 |  |  |
|  | 81.33 | 119.57 | 204.58 | 103.08 |  |  |
|  | 80.79 | 119.85 | 206.63 | 105.00 |  |  |
|  | 80.87 | 119.60 | 207.58 | 105.95 |  |  |
| Pentagalloyl glucose | 51.72 | 46.02 | 98.61 | 101.90 | 101.80 | 1.98 |
|  | 51.74 | 45.95 | 97.65 | 99.91 |  |  |
|  | 51.91 | 46.01 | 98.20 | 100.63 |  |  |
|  | 51.82 | 46.00 | 98.12 | 100.67 |  |  |
|  | 51.47 | 46.25 | 98.47 | 101.61 |  |  |
|  | 50.82 | 46.17 | 99.79 | 106.07 |  |  |
| Senkyunolide I | 11.82 | 10.05 | 21.75 | 98.80 | 96.24 | 3.00 |
|  | 11.68 | 10.09 | 21.74 | 99.70 |  |  |
|  | 11.77 | 10.09 | 21.20 | 93.46 |  |  |
|  | 11.81 | 10.09 | 21.22 | 93.26 |  |  |
|  | 11.80 | 10.13 | 21.26 | 93.40 |  |  |
|  | 11.64 | 10.11 | 21.64 | 98.84 |  |  |
| Lactiflorin | 134.23 | 107.48 | 248.51 | 106.32 | 101.55 | 3.26 |
|  | 109.86 | 107.77 | 212.99 | 95.70 |  |  |
|  | 111.05 | 107.99 | 218.94 | 99.90 |  |  |
|  | 108.74 | 107.80 | 220.07 | 103.28 |  |  |
|  | 106.54 | 108.29 | 218.23 | 103.14 |  |  |
|  | 145.52 | 107.91 | 254.47 | 100.96 |  |  |
| Senkyunolide H | 4.80 | 4.82 | 9.54 | 98.33 | 94.59 | 2.47 |
|  | 4.89 | 4.85 | 9.56 | 96.40 |  |  |
|  | 4.52 | 4.86 | 8.96 | 91.37 |  |  |
|  | 4.54 | 4.86 | 9.13 | 94.57 |  |  |
|  | 4.50 | 4.85 | 8.98 | 92.32 |  |  |
|  | 4.87 | 4.86 | 9.46 | 94.57 |  |  |
| Ononin | 12.37 | 10.91 | 22.78 | 95.47 | 92.08 | 3.10 |
|  | 12.39 | 11.03 | 22.79 | 94.39 |  |  |
|  | 12.52 | 11.09 | 22.35 | 88.58 |  |  |
|  | 12.56 | 11.06 | 22.37 | 88.68 |  |  |
|  | 12.40 | 11.03 | 22.40 | 90.74 |  |  |
|  | 12.33 | 10.97 | 22.71 | 94.64 |  |  |
| Liquiritigenin | 5.81 | 7.09 | 13.04 | 102.09 | 94.14 | 4.91 |
|  | 5.65 | 7.10 | 12.66 | 98.65 |  |  |
|  | 5.90 | 7.12 | 12.35 | 90.62 |  |  |
|  | 5.86 | 7.10 | 12.22 | 89.58 |  |  |
|  | 5.84 | 7.12 | 12.46 | 92.92 |  |  |
|  | 5.64 | 7.10 | 12.10 | 90.98 |  |  |
| Glycyrrhizic acid | 234.55 | 239.74 | 480.18 | 102.46 | 102.05 | 0.59 |
|  | 234.85 | 240.12 | 477.63 | 101.11 |  |  |
|  | 234.24 | 240.80 | 479.21 | 101.73 |  |  |
|  | 234.18 | 241.08 | 479.17 | 101.63 |  |  |
|  | 230.83 | 240.78 | 478.40 | 102.82 |  |  |
|  | 229.96 | 241.56 | 477.63 | 102.53 |  |  |

**Table S8** Quantitative analysis of three batches of XYHJ using external standard method (ESM) and QAMS methods.

| Sample ID | Lot NO. | Pentagalloyl  glucose (280 nm) | | Gallic acid (280 nm) | | | | | | Chlorogenic acid (250 nm) | | | | | | Albiflorin (250 nm) | | | | | | Paeoniflorin (250 nm) | | | | | |
| --- | --- | --- | --- | --- | --- | --- | --- | --- | --- | --- | --- | --- | --- | --- | --- | --- | --- | --- | --- | --- | --- | --- | --- | --- | --- | --- | --- |
|  |  | ESM | | ESM | | QAMS | | RE (%) | | ESM | | QAMS | | RE (%) | | ESM | | QAMS | | RE (%) | | ESM | | QAMS | | RE (%) | |
| S1 | 346210012 | 140.15 | | 210.51 | | 209.77 | | −0.35 | | 31.28 | | 31.01 | | −0.88 | | 889.22 | | 882.83 | | −0.72 | | 2301.75 | | 2288.41 | | −0.58 | |
| S2 | 346210032 | 136.04 | | 246.14 | | 244.62 | | −0.62 | | 29.53 | | 29.29 | | −0.84 | | 771.72 | | 767.20 | | −0.59 | | 2016.87 | | 2012.13 | | −0.24 | |
| S3 | 346210022 | 90.44 | | 211.25 | | 209.84 | | −0.68 | | 28.65 | | 28.34 | | −1.10 | | 871.99 | | 863.15 | | −1.02 | | 2179.54 | | 2163.16 | | −0.76 | |
| Sample ID | Lot NO. | Schaftoside (280 nm) | | | Ferulic acid (250 nm) | | | | | | Liquiritin apioside (250 nm) | | | | Liquiritin (250 nm) | | | | | | Senkyunolide I (280 nm) | | | | | |  |
|  |  | ESM | QAMS | RE (%) | ESM | | QAMS | | RE (%) | | ESM | | QAMS | RE (%) | ESM | | QAMS | | RE (%) | | ESM | | QAMS | | RE (%) | |  |
| S1 | 346210012 | 136.38 | 136.10 | −0.20 | 24.90 | | 25.47 | | 2.22 | | 142.78 | | 143.00 | 0.15 | 232.98 | | 234.47 | | 0.63 | | 35.75 | | 35.33 | | −1.16 | |  |
| S2 | 346210032 | 111.20 | 110.72 | −0.43 | 27.20 | | 27.63 | | 1.55 | | 156.85 | | 156.23 | −0.40 | 258.19 | | 258.50 | | 0.12 | | 38.30 | | 37.84 | | −1.21 | |  |
| S3 | 346210022 | 105.90 | 105.07 | −0.79 | 18.68 | | 18.61 | | −0.39 | | 109.08 | | 105.71 | −3.19 | 205.18 | | 198.77 | | −3.23 | | 25.68 | | 25.34 | | −1.35 | |  |
| Sample ID | Lot NO. | Lactiflorin (280 nm) | | | Senkyunolide H (280 nm) | | | | | | Ononin (280 nm) | | | | Liquiritigenin (280 nm) | | | | | | Glycyrrhizic acid (260 nm) | | | | | |  |
|  |  | ESM | QAMS | RE (%) | ESM | | QAMS | | RE (%) | | ESM | | QAMS | RE (%) | ESM | | QAMS | | RE (%) | | ESM | | QAMS | | RE (%) | |  |
| S1 | 346210012 | 540.86 | 536.60 | −0.79 | 12.96 | | 12.83 | | −0.98 | | 33.91 | | 33.72 | −0.56 | 20.73 | | 20.50 | | −1.10 | | 675.99 | | 679.07 | | 0.45 | |  |
| S2 | 346210032 | 625.89 | 621.64 | −0.68 | 14.95 | | 14.77 | | −1.17 | | 38.93 | | 38.69 | −0.63 | 28.78 | | 28.49 | | −1.01 | | 828.48 | | 829.39 | | 0.11 | |  |
| S3 | 346210022 | 307.02 | 301.51 | −1.83 | 7.81 | | 7.78 | | −0.41 | | 21.28 | | 21.15 | −0.63 | 11.50 | | 11.31 | | −1.74 | | 573.73 | | 576.34 | | 0.45 | |  |

Notes: The unit of content is µg/g; ESM represents external standard method; QAMS represents quantitative analysis of multiple components by single marker. RE represents relative deviation.

**Table S9** Identification of prototype components in XYHJ across various biosamples of rats.

| No. | t*_R_* (min) | Selected ion | Elemental composition | Measured mass | Mass error (× 10^−6^) | (ESI^+^) MS/MS fragmentation | (ESI^−^) MS/MS fragmentation | Identification | Class | Distribution |
| --- | --- | --- | --- | --- | --- | --- | --- | --- | --- | --- |
| P1 | 2.90 | [M−H]^−^ | C_23_H_27_O_13_S | 543.117 4 | 0.4 | − | 543.117 24, 213.020 2, 121.033 0 | Paeoniflorin sulfonate | T | F |
| P2 | 3.03 | [M+COOH]^−^ | C_24_H_29_O_13_ | 525.160 3 | −1.0 | − | 525.160 3, 479.153 4, 357.117 6, 195.065 0, 165.053 8, 121.028 9 | Albiflorin | T | UFB |
| P3 | 3.23 | [M+COOH]^−^ | C_24_H_29_O_13_ | 525.160 5 | −0.6 | − | 525.160 5, 479.155 1, 449.144 3, 357.118 4, 327.108 5, 165.055 0, 121.029 0 | Paeoniflorin | T | UFB |
| P4 | 3.38 | [M−H]^−^ | C_26_H_27_O_14_ | 563.139 6 | −1.4 | 565.156 1 | 563.139 6, 473.106 3, 443.095 6, 383.075 7, 353.064 5, 325.071 0 | Schaftoside | Fl | UF |
| P5 | 3.84 | [M−H]^−^ | C_26_H_29_O_13_ | 549.160 5 | −0.5 | − | 549.160 5, 417.117 5, 255.065 9, 135.008 2, 119.048 7 | Liquiritin apioside | Fl | PUF |
| P6 | 3.87 | [M−H]^−^ | C_21_H_21_O_9_ | 417.118 4 | −0.5 | − | 255.067 4, 135.011 0, 119.048 6 | Neoliquiritin | Fl | PUF |
| P7 | 3.87 | [M−H]^−^ | C_21_H_21_O_9_ | 417.118 9 | 0.7 | 257.082 1, 137.024 7, 109.030 4 | 417.118 9, 255.065 6, 135.008 7, 119.050 4 | Liquiritin | Fl | PUF |
| P8 | 3.93 | [M−H]^−^ | C_27_H_21_O_12_ | 537.104 4 | 2.0 | − | 537.104 4, 493.114 3, 295.061 0, 159.044 7, 135.008 9, 109.030 1 | Salvianolic acid J | OA | F |
| P9 | 4.09 | [M+H]^+^ | C_23_H_29_O_11_ | 481.170 5 | 1.0 | 319.118 2, 301.109 3, 197.083 0, 179.073 0, 161.062 5, 151.078 3, 105.035 4 | 525.160 7, 357.112 6, 327.089 9, 121.027 9 | Albiflorin R1 | T | FB |
| P10 | 4.20 | [M+Na]^+^ | C_12_H_16_O_4_Na | 247.095 8 | 4.9 | 207.103 0, 189.091 9, 179.108 4, 161.097 2 | − | Senkyunolide I | L | P |
| P11 | 4.69 | [M−H]^−^ | C_21_H_21_O_10_ | 433.114 1 | 1.4 | 273.076 4 | 271.090 5, 151.040 0 | 5-Hydroxyl liquiritin | Fl | P |
| P12 | 4.85 | [M−H]^−^ | C_21_H_21_O_9_ | 417.118 1 | −1.2 | − | 255.066 3, 135.008 4, 119.049 5 | Isoliquiritin | Fl | P |
| P13 | 5.19 | [M−H]^−^ | C_15_H_11_O_4_ | 255.065 5 | −0.8 |  | 135.007 9, 119.051 4, 91.003 7 | Liquiritigenin | Fl | PU |
| P14 | 5.53 | [M−H]^−^ | C_42_H_61_O_17_ | 837.390 1 | −1.0 | 663.371 6, 487.343 1, 469.333 4, 451.320 0 | 351.055 8, 193.035 0, 175.026 9, 113.023 2 | Macedonoside A | Tr | F |
| P15 | 5.65 | [M−H]^−^ | C_44_H_63_O_18_ | 879.401 1 | −0.3 | 705.384 6, 529.352 9, 511.343 7, 493.335 8, 451.322 2, 469.335 0, 433.297 5, 405.264 7 | 351.056 6, 193.034 2, 175.023 5, 113.024 8 | Acetoxyl-glycyrrhizin | Tr | F |
| P16 | 5.82 | [M+H]^+^ | C_12_H_13_O_2_ | 189.092 3 | 3.7 | 171.084 6, 147.044 6, 133.029 6 | − | Ligusticum lactone | L | PUFB |
| P17 | 6.05 | [M−H]^−^ | C_42_H_61_O_17_ | 837.390 8 | −0.1 | 663.374 0, 645.363 3, 627.360 0, 487.342 3, 469.333 2, 451.320 3, 423.335 1, 405.319 5 | 351.056 5, 193.032 5, 175.029 0, 113.023 4 | Licoricesaponin G2 | Tr | UF |
| P18 | 6.11 | [M−H]^−^ | C_42_H_61_O_17_ | 837.391 4 | 0.6 | 663.374 3, 645.360 1, 627.358 3, 487.343 5, 469.333 0, 451.320 9, 433.317 0, 405.319 5 | 351.056 3, 193.033 9, 175.027 8, 113.023 2 | Uralsaponin U/N | Tr | F |
| P19 | 6.14 | [M+H]^+^ | C_17_H_15_O_5_ | 299.092 9 | 3.3 | 271.061 6, 167.038 7, 153.056 5 | 269.047 8, 151.039 8 | Castanin | Fl | U |
| P20 | 6.18 | [M+H]^+^ | C_16_H_13_O_6_ | 301.072 2 | 3.3 | 285.042 8, 153.021 0 | 283.027 7, 151.039 5 | Hispidulin | Fl | U |
| P21 | 6.22 | [M+H]^+^ | C_17_H_15_O_5_ | 299.093 0 | 3.7 | 271.061 6, 167.032 2, 153.057 3 | 269.046 6 | Isocastanin | Fl | U |
| P22 | 6.28 | [M+H]^+^ | C_15_H_11_O_5_ | 271.061 6 | 3.7 | 253.051 2, 225.055 9, 153.018 7 | 269.045 3, 241.085 6 | Aloe-emodin | O | U |
| P23 | 6.32 | [M+H]^+^ | C_16_H_13_O_4_ | 269.082 7 | 4.8 | 254.058 2, 253.051 5, 226.063 5, 225.057 3, 197.061 6, 169.067 2 | − | Formonentin | Fl | UFB |
| P24 | 6.36 | [M−H]^−^ | C_42_H_61_O_16_ | 821.395 9 | −0.1 | 647.383 2, 471.353 5, 453.337 7 | 351.056 2, 193.034 7, 175.024 9, 113.024 2 | 18*β*-Glycyrrhizin | Tr | UF |
| P25 | 6.45 | [M+H]^+^ | C_21_H_21_O_6_ | 369.134 9 | 3.0 | 313.072 7, 298.047 2, 285.045 6, 271.061 7, 243.061 5 137.060 8 | 367.117 4, 337.037 9, 283.027 1 | Glicoricone | Fl | UF |
| P26 | 6.69 | [M−H]^−^ | C_15_H_11_O_4_ | 255.066 7 | 3.9 | 147.046 0, 137.025 9, 119.051 5 | 135.008 3, 119.050 6, 91.019 0 | Isoliquiritigenin | Fl | U |
| P27 | 6.83 | [M−H]^−^ | C_12_H_11_O_3_ | 203.071 4 | 3.0 | 187.078 3, 149.025 7 | 151.076 6 | Senkyunolide C or 3-Butylidene-7-hydroxyphthalide | L | U |
| P28 | 7.07 | [M+H]^+^ | C_21_H_21_O_6_ | 369.134 6 | 2.2 | 313.072 3, 351.125 9, 298.051 5, 285.078 3, 271.061 6, 270.055 5, 255.067 5, 243.062 9, 123.044 8 | 367.118 4, 287.610 8 | Glycycoumarin | C | F |
| P29 | 7.21 | [M+H]^+^ | C_16_H_13_O_5_ | 285.077 4 | 3.9 | 270.054 7, 242.059 0, 153.019 9 | 283.061 1, 211.040 7 | Acacetin | Fl | UF |
| P30 | 7.22 | [M−H]^−^ | C_36_H_53_O_10_ | 645.361 9 | −3.1 | 471.347 8, 453.337 3, 435.325 4, 407.280 5 | 469.329 6 | Starrhizin | Tr | PBF |
| P31 | 7.25 | [M−H]^−^ | C_12_H_11_O_3_ | 203.071 4 | 3.0 | 187.076 1, 149.025 1 | 203.071 4, 161.097 3 | Senkyunolide C or 3-Butylidene-7-hydroxyphthalide | L | U |
| P32 | 7.38 | [M+H]^+^ | C_22_H_23_O_6_ | 383.150 1 | 1.6 | 355.264 9, 327.089 4, 315.088 7, 189.092 7, | 381.134 3, 355.118 8 | Licoricone | Fl | / |
| P33 | 7.44 | [M+H]^+^ | C_12_H_15_O_2_ | 191.107 3 | 0.5 | 173.097 9, 163.113 1, 145.104 0, 117.074 0 |  | Ligustilide | L | FB |
| P34 | 7.49 | [M+H]^+^ | C_20_H_17_O_6_ | 353.103 7 | 3.4 | 311.057 8, 299.057 4, 283.065 3, 153. 0201 | 351.088 5 | Licoisoflavone B or Semilicoisoflavone B or Allolicoisoflavone B | Fl | UF |
| P35 | 7.95 | [M+H]^+^ | C_20_H_19_O_6_ | 355.118 2 | 3.1 | 299.055 7, 271.095 1, 121.030 8 | 201.089 9 | Isolicoflavonol | Fl | / |
| P36 | 8.29 | [M+H]^+^ | C_21_H_19_O_6_ | 367.119 9 | 4.6 | 353.104 1, 313.069 5, 311.057 3, 297.078 2, 153.021 8 | 351.085 9 | Glycyrrhiza isoflavone B | Fl | F |
| P37 | 8.79 | [M+H]^+^ | C_26_H_33_O_5_ | 325.234 6 | 4.2 | 369.170 5, 351.127 4, 221.119 6, 189.092 5, 123.039 1 | 423.188 7 | Licoricidin | Fl | F |
| P38 | 9.35 | [M−H]^−^ | C_25_H_25_O_6_ | 421.165 6 | 1.2 | 367.118 3, 311.056 3, 191.107 5 | 421.164 9 | Angustone A | Fl | / |

Notes: Fl, T, Tr, OA, L, C, O represent flavonoids, terpenoids, triterpenoids, organic acids, lactones, coumarins, and other components, respectively; P, U, B, F meant plasma, urine, bile and feces, respectively. Qualitative analysis was conducted using UPLC-Q/TOF-MS with an ACQUITY UPLC RP C_18_ Column (50 mm × 2.1 mm, 1.7 µm). The mobile phase consisted of 0.1% formic acid in water (A) and 0.1% formic acid in acetonitrile (B). The column temperature was maintained at 40 °C with a flow rate of 0.3 mL/min and an injection volume of 2 µL. The elution gradient was optimized as follows: 0–0.5 min (5% B), 0.5–10 min (5%–80% B), 10–12 min (80%–100% B), 12–13 min (100% B), 13–14 min (10%–5% B), and 14–15 min (5% B). The mass spectrometry conditions included an ESI source with a capillary voltage of 3.0 kV (ESI^+^) or –2.5 kV (ESI^–^), cone voltage of 30 V, desolvation gas temperature of 300 °C, and source temperature of 100 °C. The mass accuracy correction was performed using sodium formate and leucine-enkephalin.

**Table S10** Identification of metabolites derived from XYHJ in different biosamples of rats.

| No. | *t*_R_ (min) | Selected ion | Elemental composition | Measured mass | Mass error (× 10^−6^) | (ESI^+^) MS/MS fragmentation | (ESI^−^) MS/MS fragmentation | Identification | Class | Distribution |
| --- | --- | --- | --- | --- | --- | --- | --- | --- | --- | --- |
| M1 | 2.74 | [M−H]^−^ | C_23_H_27_O_12_ | 495.150 5 | 0.4 | − | 495.150 5, 465.139 8, 181.050 2, 137.061 4 | Paeoniflorin + O | T | UFB |
| M2 | 2.84 | [M−H]^−^ | C_6_H_5_O_6_S | 204.981 0 | 1.5 | − | 125.024 8 | Benzaldehyde → Phenol + 2O + SO_3_ | T | U |
| M3 | 3.62 | [M−H] ^−^ | C_27_H_29_O_14_ | 577.154 7 | −1.7 | 579.170 3 | 563.136 4, 443.172 3, 383.079 2, 353.065 9 | Schaftoside + O | FI | F |
| M4 | 3.81 | [M−H] ^−^ | C_21_H_19_O_10_ | 431.098 1 | 0.7 | 433.113 7, 257.081 7, 147.045 1, 137.025 4 | 431.098 1, 255.065 9, 135.009 0, 119.048 8 | Isoliquiritigenin or Liquiritigenin+GluA | Fl | B |
| M5 | 3.87 | [M−H]^−^ | C_21_H_19_O_10_ | 431.097 6 | −0.5 | 257.082 3, 137.024 7, 119.050 5 | 255.066 3, 135.008 4 | Isoliquiritigenin or Liquiritigenin+GluA | Fl | PU |
| M6 | 3.87 | [M−H]^−^ | C_18_H_23_O_9_ | 383.134 8 | 1.6 | − | 207.029 3, 179.038 7 | Ligustilide + 2H + O + GluA | L | / |
| M7 | 3.88 | [M+H]^+^ | C_15_H_22_NO_5_S | 328.122 4 | 1.5 | 207.102 5, 189.094 1, 161.097 4, 133.067 3 | − | Ligustilide + O + 2H + Cys | L | PUF |
| M8 | 3.90 | [M−H] ^−^ | C_21_H_21_O_13_S | 513.070 4 | 0.2 | − | 337.037 4, 255.066 0, 137.023 9, 119.050 8 | Isoliquiritigenin or Liquiritigenin + 2H + GluA + SO_3_ | Fl | B |
| M9 | 3.94 | [M−H]^−^ | C_18_H_23_O_10_ | 399.129 2 | 0.3 | 225.115 6, 207.104 5, 133.027 8 | 223.082 8, 205.086 2 | Senkyunolide I or Senkyunolide H + GluA | L | U |
| M10 | 3.99 | [M−H]^−^ | C_21_H_21_O_10_ | 433.112 4 | −2.5 | 259.097 5, 137.025 0, 121.066 4 | 257.080 5, 175.026 5 | Isoliquiritigenin or Liquiritigenin + 2H + GluA | Fl | PU |
| M11 | 4.01 | [M−H]^−^ | C_21_H_21_O_10_ | 433.112 1 | −3.2 | 259.096 2, 137.025 8, 121.068 7 | 257.081 7, 175.026 7 | Isoliquiritigenin or Liquiritigenin + 2H + GluA | Fl | PU |
| M12 | 4.04 | [M−H]^−^ | C_21_H_19_O_10_ | 431.098 8 | 2.3 | 257.082 5, 137.025 7, 119.051 6 | 255.065 3, 135.009 8 | Isoliquiritigenin or Liquiritigenin + GluA | Fl | PU |
| M13 | 4.08 | [M+H]^+^ | C_12_H_15_O_3_ | 207.102 7 | 2.9 | 189.091 7, 171.082 6, 153.071 6, 143.086 9, 128.062 3, 91.056 4 | − | Ligusticum lactone + H_2_O | L | U |
| M14 | 4.20 | [M−H]^−^ | C_12_H_15_O_7_S | 303.053 0 | −2.6 | 207.103 4, 189.092 6, 161.096 4 | 223.092 3 | Senkyunolide I or Senkyunolide H + SO_3_ | L | / |
| M15 | 4.20 | [M+H]^+^ | C_12_H_15_O_3_ | 207.103 0 | 4.3 | 189.091 6, 171.082 2, 153.075 1, 143.087 7 | − | Ligusticum lactone + H_2_O | L | U |
| M16 | 4.47 | [M−H] ^−^ | C_15_H_13_O_7_S | 337.038 9 | 2.1 | 137.022 4 | 257.082 0, 151.039 7, 137.024 7, 119.050 9, 79.956 6 | Isoliquiritigenin or Liquiritigenin + 2H + SO_3_ | Fl | PU |
| M17 | 4.68 | [M−H]^−^ | C_18_H_23_O_9_ | 383.133 4 | −2.1 | − | 207.029 7, 189.055 8, 179.034 2, 175.024 7 | Ligustilide + 2H + O + GluA | L | U |
| M18 | 4.69 | [M−H]^−^ | C_22_H_21_O_12_ | 475.088 4 | 1.5 | 301.071 4, 285.032 9, 153.019 7 | 299.054 0, 255.069 0, 237.077 1, 225.063 6, 121.031 4 | Hispidulin + GluA | FI | U |
| M19 | 4.75 | [M+H]^+^ | C_12_H_17_O_3_ | 209.118 7 | 4.3 | 193.052 3, 181.086 2, 163.113 913 5.0824 | − | Ligustilide + O + 2H | L | PU |
| M20 | 4.80 | [M−H]^−^ | C_21_H_21_O_13_S | 513.069 0 | −2.5 | − | 433.112 5, 257.080 3, 175.026 1 | Isoliquiritigenin or Liquiritigenin + 2H + GluA + SO_3_ | Fl | P |
| M21 | 4.80 | [M−H]^−^ | C_21_H_21_O_10_ | 433.113 7 | 0.5 | 259.098 5, 137.028 0, 121.065 7 | 257.082 4, 175.025 3 | Isoliquiritigenin or Liquiritigenin+ 2H + GluA | Fl | PU |
| M22 | 4.85 | [M+H]^+^ | C_12_H_17_O_3_ | 209.117 8 | 0.0 | 193.052 9, 181.088 0, 163.113 613 5.0815 | − | Ligustilide + O + 2H | L | PU |
| M23 | 5.05 | [M−H]^−^ | C_21_H_21_O_13_S | 513.079 7 | −0.6 | − | 433.113 0, 257.080 2, 175.026 0 | Isoliquiritigenin or Liquiritigenin + 2H + GluA + SO_3_ | Fl | P |
| M24 | 5.05 | [M−H]^−^ | C_21_H_19_O_10_ | 431.098 1 | 0.7 | 257.082 3, 137.025 2, 119.051 0 | 255.066 4, 135.009 1, 91.024 0 | Isoliquiritigenin or Liquiritigenin+GluA | Fl | PU |
| M25 | 5.07 | [M−H]^−^ | C_12_H_13_O_4_ | 221.081 4 | 0.0 | − | 203.071 2, 193.033 9, 175.024 9, 147.045 2, 135.046 1 | Ligustilide + 2O − 2H | L | U |
| M26 | 5.10 | [M+H]^+^ | C_21_H_19_O_11_ | 447.091 9 | −1.8 | 271.061 1 | 269.045 9 | Aloe emodin + GluA | O | U |
| M27 | 5.10 | [M+H]^+^ | C_17_H_24_NO_6_S | 370.132 9 | 1.4 | 352.122 7, 207.103 0, 189.093 2, 179.017 4, 161.097 5 | − | Ligustilide + O + 2H + NAC | L | PU |
| M28 | 5.21 | [M−H]^−^ | C_15_H_9_O_4_ | 253.050 0 | −0.4 | 255.066 9 | 135.008 6, 119.050 6, 117.033 7 | Isoliquiritigenin or Liquiritigenin−2H | Fl | U |
| M29 | 5.23 | [M+H]^+^ | C_16_H_13_O_5_ | 285.077 1 | 2.8 | 270.053 8, 242.059 4, 241.050 4, 225.056 9, 213.056 0 | 283.061 3 | Formonentin + O | FI | U |
| M30 | 5.33 | [M+H]^+^ | C_27_H_29_O_12_ | 545.165 4 | −0.9 | 369.134 5, 227.181 4 | 543.147 0, 367.118 0 | Glicoricone + GluA | Fl | / |
| M31 | 5.46 | [M−H]^−^ | C_15_H_11_O_7_S | 335.021 5 | −3.0 | 337.039 6, 257.084 4, 147.045 8, 137.026 3 | 255.064 9, 135.009 0, 119.050 4 | Isoliquiritigenin or Liquiritigenin+SO_3_ | Fl | PU |
| M32 | 5.50 | [M−H]^−^ | C_15_H_9_O_7_S | 333.007 7 | 2.4 | 335.021 9, 255.066 8, 137.025 3 | 253.050 3, 135.010 0, 119.050 4, 117.034 1 | Isoliquiritigenin or Liquiritigenin − 2H + SO_3_ | Fl | U |
| M33 | 5.53 | [M−H]^−^ | C_15_H_11_O_7_S | 335.022 0 | −1.5 | 337.038 6, 257.083 1, 147.047 4, 137.025 7 | 255.065 3, 135.008 4, 119.050 1 | Isoliquiritigenin or Liquiritigenin + SO_3_ | Fl | PU |
| M34 | 5.56 | [M−H]^−^ | C_12_H_11_O_7_S | 299.023 1 | 2.0 |  | 219.066 1, 79.957 9 | Senkyunolide I or Senkyunolide H + SO_3_ −4H | L | / |
| M35 | 5.60 | [M−H]^−^ | C_26_H_23_O_12_ | 527.119 1 | 0.2 | 353.099 | 351.087 | Licoisoflavone B or Semilicoisoflavone B or Allolicoisoflavone B + GluA | Fl | / |
| M36 | 5.65 | [M−H]^−^ | C_44_H_63_O_18_ | 879.401 5 | 0.1 | 881.414 8 | 838.392 5, 661.338 4, 485.220 2, 351.054 4, 193.032 0 | Licoricesaponin G2 + C_2_H_2_O | Tr | F |
| M37 | 5.68 | [M−H]^−^ | C_15_H_11_O_7_S | 335.022 5 | 0.0 | 337.038 8, 257.083 1, 147.045 0, 137.025 3 | 255.065 8, 135.008 7, 119.050 3 | Isoliquiritigenin or Liquiritigenin + SO_3_ | Fl | PU |
| M38 | 5.71 | [M+H]^+^ | C_17_H_22_NO_5_S | 352.122 5 | 1.7 | 189.092 8, 143.097 1 | − | Ligustilide + NAC | L | U |
| M39 | 5.83 | [M+H]^+^ | C_15_H_13_O_5_ | 273.077 6 | 4.8 | 255.070 4, 225.057 8, 153.020 8 | 271.060 3, 243.032 4 | Aloe emodin + 2H | O | U |
| M40 | 5.89 | [M−H]^−^ | C_26_H_23_O_12_ | 527.119 3 | 0.6 | 353.102 9 | 351.086 1 | Licoisoflavone B or Semilicoisoflavone B or Allolicoisoflavone B + GluA | Fl | / |
| M41 | 5.98 | [M−H]^−^ | C_12_H_13_O_6_S | 285.042 9 | −1.4 | 287.060 3, 207.103 3, | 205.087 1, 187.076 6, 177.094 2, 159.082 2 | Ligustilide + O + SO_3_ | L | U |
| M42 | 6.14 | [M+H]^+^ | C_17_H_15_O_5_ | 299.091 9 | 5.0 | 299.093 4, 284.070 1, 256.074 0, 213.056 7 | 297.076 5 | Formonentin + O + CH_2_ | FI | U |
| M43 | 6.17 | [M+H]^+^ | C_16_H_13_O_6_ | 301.072 5 | 4.3 | 286.049 9, 258.053 4, 257.046 8, 229.054 6 | 299.056 2 | Formonentin+2O | FI | U |
| M44 | 6.17 | [M−H]^−^ | C_12_H_13_O_7_S | 301.037 6 | −2.0 | 285.044 6 | 221.082 | Senkyunolide I or Senkyunolide H + SO_3_ − 2H | L | P |
| M45 | 6.22 | [M+H]^+^ | C_17_H_15_O_5_ | 299.093 0 | 1.1 | 299.093 0, 284.069 2, 256.074 9, 213.056 9 | 297.077 2 | Formonentin + O + CH_2_ | FI | FU |
| M46 | 6.54 | [M+H]^+^ | C_32_H_49_O_7_ | 545.348 8 | 1.8 | 545.348 8 | − | Glycyrrhetinic acid + C_2_H_2_O + 2O | Tr | F |
| M47 | 6.60 | [M−H]^−^ | C_12_H_11_O_6_S | 283.027 9 | 1.1 | 285.045 8, 205.088 4 | 203.071 8, 175.075 1, 147.081 1, 157.063 9 | Ligustilide + O + SO_3_ −2H | L | P |
| M48 | 6.71 | [M−H]^−^ | C_15_H_13_O_7_S | 337.038 3 | 0.3 | 137.025 6 | 257.081 1, 151.040 1, 137.024 1, 119.050 2, 79.963 4 | Isoliquiritigenin or Liquiritigenin + 2H + SO_3_ | Fl | PU |
| M49 | 6.80 | [M+H]^+^ | C_30_H_47_O_5_ | 487.341 8 | −1.0 | 469.331 4, 451.322 2, 437.341 1, 405.299 3, 315.092 8 | − | Glycyrrhetinic acid + O | Tr | PFB |
| M50 | 6.82 | [M+H]^+^ | C_12_H_13_O_3_ | 205.086 7 | 1.0 | 187.077 7, 177.082 9, 159.082 3, 149.025 7 | − | Ligustilide + O − 2H | L | U |
| M51 | 6.82 | [M−H]^−^ | C_9_H_11_O_5_S | 231.033 0 | 1.3 | − | 151.076 1 | C_9_H_12_O_2_ + SO_3_ | T | U |
| M52 | 6.95 | [M+H]^+^ | C_30_H_47_O_5_ | 487.343 3 | 2.1 | 469.335 0, 451.319 0, 441.337 1, 423.328 5, 407.322 0, 315.193 4 | − | Glycyrrhetinic acid + O | Tr | PFB |
| M53 | 7.09 | [M−H]^−^ | C_15_H_13_O_7_S | 337.038 3 | 0.3 | 137.027 9 | 257.081 5, 151.039 9, 137.024 2, 79.958 2 | Isoliquiritigenin or Liquiritigenin + 2H + SO_3_ | Fl | PU |
| M54 | 7.22 | [M+H]^+^ | C_12_H_13_O_3_ | 205.087 1 | 2.9 | 187.076 6, 177.089 8, 149.025 8 | − | Ligustilide + O − 2H | L | / |
| M55 | 7.35 | [M+H]^+^ | C_30_H_47_O_5_ | 487.344 0 | 3.5 | 469.326 8, 451.260 3, 441.339 8, 423.180 5, 405.311 9, | − | Glycyrrhetinic acid + O | Tr | PFB |
| M56 | 7.60 | [M+H]^+^ | C_30_H_47_O_5_ | 487.341 8 | −1.0 | 469.331 7, 451.323 5, 441.281 0, 423.268 7, 315.198 1 | − | Glycyrrhetinic acid + O | Tr | PFB |
| M57 | 7.64 | [M−H]^−^ | C_15_H_11_O_7_S | 335.022 7 | 0.6 | 337.040 2, 257.083 3, 137.026 1 | 255.065 4, 135.008 2, 119.050 2 | Isoliquiritigenin or Liquiritigenin + SO_3_ | Fl | PU |
| M58 | 7.65 | [M+H]^+^ | C_30_H_45_O_6_ | 501.322 4 | 1.6 | 501.322 4 | − | Glycyrrhetinic acid + 2O −2H | Tr | FB |
| M59 | 7.70 | [M+H]^+^ | C_30_H_47_O_5_ | 487.342 8 | 1.0 | 469.331 9, 451., 3 216 441.278 8, 423.329 8, 405.312 7, 317.209 1 | − | Glycyrrhetinic acid + O | Tr | PFB |
| M60 | 7.78 | [M+H]^+^ | C_30_H_45_O_6_ | 501.323 0 | 2.8 | 501.323 | − | Glycyrrhetinic acid + 2O −2H | Tr | FB |
| M61 | 7.81 | [M+H]^+^ | C_30_H_47_O_5_ | 487.344 5 | 4.5 | 469.333 3, 451.324 2, 441.284 2, 423.326 4, 317.190 0 | − | Glycyrrhetinic acid + O | Tr | PFB |
| M62 | 7.96 | [M+H]^+^ | C_32_H_49_O_6_ | 529.353 6 | 1.3 | 487.342 8, 469.332 8, 451.320 3, 441.337 2, 423.329 5, 405.316 0, 315.196 6 | − | Glycyrrhetinic acid + C_2_H_2_O + O | Tr | PF |
| M63 | 8.07 | [M+H]^+^ | C_30_H_45_O_5_ | 485.326 1 | −1.2 | 485.326 1 | − | Glycyrrhetinic acid + O −2H | Tr | F |
| M64 | 8.08 | [M+H]^+^ | C_30_H_47_O_5_ | 487.342 2 | −0.2 | 469.334 6, 451.324 5, 423.325 7, 405.318 4, 315.192 6 | − | Glabrolide + 2H + O | Tr | F |
| M65 | 8.25 | [M+H]^+^ | C_30_H_45_O_4_ | 469.333 1 | 2.8 | 451.323 1, 437.283 4, 435.326 7, 423.328 5, 405.352 2 | − | Glabrolide | Tr | F |
| M66 | 8.95 | [M−H]^−^ | C_30_H_45_O_4_ | 469.332 4 | 1.3 | 471.348 9, 453.339 1,, 407.334 4 | 469.332 4, 453.301 8, 425.342 2 | 18*α* or 18*β*−Glycyrrhetinic acid | Tr | PFB |

Notes: Fl, T, Tr, OA, L, C, O represent flavonoids, terpenoids, triterpenoids, organic acids, lactones, coumarins, and other components, respectively; P, U, B, F meant plasma, urine, bile and feces, respectively.

**Table S1****1** Pharmacokinetic parameters of nine components in rats after administration of XYHJ.

| Analytes | *C*_max_ (ng/mL) | *T*_1/2_ (h) | *T*_max_ (h) | *MRT*_0-t_ (h) | *MRT*_0-∞_(h) | *AUC*_0-t_ (ng·h/mL) | *AUC*_0-∞_ (ng·h/mL) |
| --- | --- | --- | --- | --- | --- | --- | --- |
| Liquiritigenin | 4.40±2.31 | 56.25±30.41 | 5.83±3.92 | 8.79±3.83 | 80.61±29.74 | 37.97±19.12 | 130.80±44.32 |
| Isoliquiritigenin | 1.43±0.31 | 32.89±18.15 | 14.13±9.12 | 15.74±8.45 | 48.03±25.65 | 23.48±14.58 | 30.70±21.80 |
| Liquiritin apioside | 5.05±1.94 | 10.75±5.61 | 1.00±0.55 | 4.90±0.58 | 15.96±6.29 | 30.70±14.32 | 52.53±25.74 |
| Neoliquiritin | 0.79±0.30 | 7.68±4.78 | 0.79±0.64 | 2.55±0.24 | 10.98±6.78 | 2.50±0.72 | 5.05±2.51 |
| Senkyunolide I | 32.41±17.18 | 1.96±0.90 | 0.46±0.29 | 2.03±0.55 | 2.71±1.22 | 59.26±28.03 | 62.63±27.72 |
| Ligustilide | 21.29±5.87 | 0.95±0.04 | 0.54±0.25 | 1.03±0.38 | 1.51±0.05 | 27.18±13.20 | 31.17±8.61 |
| Atractylenolide III | 3.91±1.10 | 7.51±4.00 | 0.50±0.27 | 4.04±1.34 | 10.27±5.95 | 12.26±3.00 | 18.47±8.68 |
| Glycyrrhetinic acid | 1 020.68±349.22 | 29.25±16.13 | 36.00±18.59 | 37.43±5.13 | 55.86±30.80 | 27 328.54±9 629.95 | 39 036.56±7 635.04 |
| Paeoniflorin | 189.27±35.26 | 8.11±2.63 | 4.92±2.52 | 11.02±3.49 | 11.04±4.13 | 2 259.92±940.79 | 2 048.18±964.31 |

Notes: For pharmacokinetics study, chromatographic conditions were achieved using a Waters ACQUITY™ RP C_18_ column (2.1 mm×100 mm, 1.7 µm). The mobile phase consisted of A (water with 0.1% formic acid) and B (acetonitrile with 0.1% formic acid). The gradient elution program was set as follows: 5% B from 0 to 0.5 min, 5%−30% B from 0.5 to 4 min, 30%−40% B from 4 to 7.5 min, 40%−100% B from 7.5 to 10 min, 100% B from 10 to 11 min, 100%−5% B from 11 to 11.5 min, and 5% B from 11.5 to 12 min. The column temperature was maintained at 40 °C, with an injection volume of 2 μL and a flow rate of 0.3 mL/min. The MS conditions employed an ESI source with the ion source temperature kept at 150 °C. Nitrogen was used as the desolvation gas and cone gas. The cone gas flow rate was controlled at 150 L/hr, the desolvation gas flow rate at 1 000 L/hr, and the desolvation gas temperature was set at 600 °C. The capillary voltage was set at 2.0 kV in ESI + mode. Data acquisition was performed in MRM or SIR mode.

**Table S12** Score of Q-marker calculated by multi-dimensional weighted radar chart model integrating five key factors.

| Compounds | Compatibility environment | Specificity | Effectiveness | Traceability | Measurability |
| --- | --- | --- | --- | --- | --- |
| Gallic acid | 3.454 125 | 10.025 811 62 | 3.917 725 | 6.990 209 6 | 5.192 097 4 |
| Chlorogenic acid | 4.605 5 | 6.326 037 178 | 3.917 725 | 2.712 584 6 | 4.104 077 |
| Albiflorin | 3.454 125 | 12.957 399 33 | 7.835 45 | 2.281 4 | 8.860 166 1 |
| Paeoniflorin | 3.454 125 | 15.597 7 | 12.193 919 06 | 3.814 500 8 | 16.000 3 |
| Schaftoside | 2.302 75 | 4.719 864 02 | 7.835 45 | 6.387 92 | 4.692 088 |
| Ferulic acid | 3.454 125 | 5.285 280 645 | 3.917 725 | 3.627 426 | 4.064 076 2 |
| Liquiritin apioside | 2.302 75 | 8.274 969 793 | 7.835 45 | 3.506 511 8 | 4.784 089 7 |
| Liquiritin | 2.302 75 | 7.592 960 36 | 7.835 45 | 4.040 359 4 | 5.276 098 9 |
| Pentagalloyl glucose | 3.454 125 | 8.638 006 26 | 3.917 725 | 3.433 507 | 4.736 088 8 |
| Senkyunolide I | 3.454 125 | 4.608 340 465 | 9.824 870 755 | 4.145 303 8 | 4.152 077 9 |
| Lactiflorin | 3.454 125 | 6.825 553 52 | 3.917 725 | 3.689 023 8 | 6.748 126 5 |
| Senkyunolide H | 3.454 125 | 3.899 425 | 3.917 725 | 9.125 6 | 4.000 075 |
| Ononin | 2.302 75 | 4.660 202 818 | 3.917 725 | 3.235 025 2 | 4.128 077 4 |
| Liquiritigenin | 2.302 75 | 4.457 042 775 | 9.802 147 95 | 4.412 227 6 | 4.036 075 7 |
| Glycyrrhizic acid | 2.302 75 | 10.778 400 64 | 15.670 9 | 7.391 736 | 7.668 143 8 |
| Average | 3.070 333 333 | 7.643 132 962 | 7.083 847 518 | 4.586 222 373 | 5.896 110 55 |

**References**

Chen, Y. Q., Fan, X. S., Zhou, L. P., Hu, F. Y., & Pengli, W. (2022). Screening and evaluation of quality markers from Shuangshen Pingfei formula for idiopathic pulmonary fibrosis using network pharmacology and pharmacodynamic, phytochemical, and pharmacokinetic analyses. *Phytomedicine*, *100*, 154040.

Gu, X., Li, Z., Feng, Y., Hu, Y., Xie, K., Shang, J., et al. (2024). A mathematical multiple-dimensional strategy for Q-markers identification based on “five principles”: Tianshu Capsule for migraine treatment as an example. *Journal of Ethnopharmacology*, *333*, 118428.

He, L., Liu, Y., Yang, K., Zou, Z., Fan, C., Yao, Z., et al. (2021). The discovery of Q-markers of Qiliqiangxin Capsule, a traditional Chinese medicine prescription in the treatment of chronic heart failure, based on a novel strategy of multi-dimensional “radar chart” mode evaluation. *Phytomedicine*, *82*, 153443.

Hu, L., Chen, G., Chen, J., Zou, Z., Qiu, Y., Du, J., T., et al. (2024). Quantitative ternary network-oriented discovery of Q-markers from traditional Chinese medicine prescriptions: Bu-Zhong-Yi-Qi-Tang as a case study. *Phytomedicine*, *124*, 155332.

Huang, L., Xu, D. Q., Yue, S., Chen, Y. Y., Fu, R. J., Lu, Z., et al. (2021a). Research on Q-markers of *Eupatorium lindleyanum* based on analytic hierarchy process-entropy weight method and network pharmacology. *China Journal of Chinese Materia Medica*, *46*(21), 5523–5531.

Huang, L., Xu, D. Q., Yue, S., Chen, Y. Y., Fu, R. J., Lu, Z., et al. (2021b). Research on Q-markers of Gangban Gui based on analytic hierarchy process-entropy weight method and fingerprint. *China Journal of Chinese Materia Medica*, *46*(21), 5523–5531.

Huang, L., Xu, D. Q., Yue, S., Chen, Y. Y., Fu, R. J., Lu, Z., et al. (2021c). Research on Q-markers of Lanzhou Buzheng based on analytic hierarchy process-entropy weight method and network pharmacology. *China Journal of Chinese Materia Medica*, *46*(21), 5523–5531.

Huang, L., Xu, D. Q., Yue, S., Chen, Y. Y., Fu, R. J., Lu, Z., et al. (2021d). Research on Q-markers of *Schisandra chinensis* for treating bronchial asthma based on analytic hierarchy process-entropy weight method, fingerprint, and network pharmacology. *China Journal of Chinese Materia Medica*, *46*(21), 5523–5531.

Liu, L. X., Li, H. B., Zhang, J. Y., Shi, D. F., Wang, Z. Z., Yao, X. S., et al. (2022). Identification of Q-markers from Hedan Tablet by employing “spider-web” mode and taking compounds’ hepatotoxicity into account. *Chinese Journal of Natural Medicines*, *20*(10), 792–800.

Liu, L. X., Li, H. B., Zhang, J. Y., Shi, D. F., Wang, Z. Z., Yao, X. S., et al. (2023). Quality markers screening of traditional Chinese medicine prescriptions based on the multi-factor analysis strategy: Jin-Zhen Oral Liquid as a case. *Arabian Journal of Chemistry*, *16*(8), 104935.

Tang, X. Y., Wang, X. X., Tan, Z. X., Xie, L. Q., Tang, Z. L., Li, Z. T., et al. (2025). Excavation of Q-markers from Ba-Zi-Bu-Shen capsule through efficacy evaluation, chemical profile, three-dimensions-overall analysis and anti-osteoporosis bioactivity based on combinatorial algorithm. *Journal of Ethnopharmacology*, *337*, 118850.

Wang, Q., Chen, G. T., Chen, X. T., Liu, Y. H., Qin, Z. F., Lin, P., et al. (2023). Development of a three-step-based novel strategy integrating DMPK with network pharmacology and bioactivity evaluation for the discovery of Q-markers of traditional Chinese medicine prescriptions: Danlou tablet as an example. *Phytomedicine*, *108*, 154511.

Zeng, X. T., Chen, Y. Y., Yue, S. J., Xu, D. Q., Fu, R. J., Yang, J., et al. (2022). A three-dimensional integration strategy for Q-markers identification: Taken *Euphorbia Pekinensis Radix* as an example. *Journal of Pharmaceutical and Biomedical Analysis*, *221*, 115046.

Zhang, T. J., Bai, G., Chen, C. Q., Xu, J., Han, Y. Q., Gong, S. X., et al. (2018). Based on “five principles” combined with spider web model to identify quality markers in Shujin Huoxue capsule. *Chinese Traditional and Herbal Drugs*, *49*(1), 1–13.

.
